# Anomerization of *N*-Acetylglucosamine Glycosides Promoted by Dibromomethane and Dimethylformamide

**DOI:** 10.3390/molecules30071483

**Published:** 2025-03-27

**Authors:** Natalie B. Condino, Doriane Rousseau, Esperance Mutoni, Jeffrey Davidson, Lara K. Watanabe, France-Isabelle Auzanneau

**Affiliations:** 1 Department of Chemistry, University of Guelph, Guelph, ON N1G 2W1, Canada; ncondino@uoguelph.ca (N.B.C.); lwatanab@uoguelph.ca (L.K.W.); 2 Fareva Valdepharm, Parc Industriel d’Incarville CS 10606, 27106 Val de Reuil, France; doriane.rousseau04@gmail.com; 3 Sun Pharma Canada Inc., 126 East Drive, Brampton, ON L6T 1C1, Canada; esperance_mutoni@outlook.com; 4 Toronto Research Chemicals, 20 Martin Ross Avenue, Toronto, ON M3J 2K8, Canada; jeffrey.davidson@lgcgroup.com

**Keywords:** anomerization, *N*-acetylglucosamine, nucleophilic substitution

## Abstract

In previous quests to synthesize fragments of tumor-associated carbohydrate antigens (TACAs), we determined that bromoalkyl β glycosides of *N*-acetylglucosamine were labile and incompatible with some of the synthetic conditions required for the preparation of oligosaccharides. While *N*-acetylglucosamine chloroalkyl β glycosides are common intermediates for oligosaccharide synthesis, they exhibit poor yields upon subsequent reactions used to introduce the oxyamine required for further conjugation. Thus, we looked to synthesize these TACAs using chloroalkyl β glycosides and substitute the chlorine for bromine at a later synthetic stage. Upon substitution of the bromine for chlorine using sodium bromide in a dibromomethane (DBM) dimethylformamide (DMF) mixture, we observed the unexpected anomerization of the *N*-acetylglucosamine β glycosides, yielding up to 90% of the α glycosides. We describe our studies of this unexpected anomerization and report on how the anomeric ratios can be controlled experimentally. Interestingly, we also report the anomerization of alkyl β glycosides of *N*-acetylglucosamine in a mixture of DBM and DMF without sodium bromide. Further studies are being conducted to determine the mechanism of this anomerization and the scope of this reaction.

## 1. Introduction

Glucosamines are a class of monosaccharides that are a major building block of many biological molecules [1]. Specifically, *N-*acetyl-d-glucosamine (Glc*N*Ac) is commonly found at the reducing end of oligosaccharides, especially in cell-surface antigens such as tumor-associated carbohydrate antigens (TACAs) [2]. It has been well established by the Crich group and others that the *N*-acetyl group in Glc*N*Ac has a major impact on the reactivity and function of the saccharide in comparison to its glucose analog [3,4,5,6,7,8,9]. It is important to consider these effects during oligosaccharide synthesis. We are interested in conjugating different fragments of TACAs to carrier molecules through these terminal β Glc*N*Ac residues to allow for immunochemical testing. In particular, we intended to conjugate TACAs (Lewis B and Lewis A) to the immunostimulant PS A1 zwitterionic polysaccharide using an oxime linkage as described by Andreana and co-workers [10,11,12,13]. Our strategy involved the synthesis of the TACAs bearing a 9-bromononyl glycoside at the reducing-end Glc*N*Ac residue with the intent to displace the bromide with *N-*hydroxyphthalimide in mild conditions [14], which would, after deprotection, yield the aminooxy functional group needed for conjugation to PS A1. However, we observed that 9-bromononyl glycosides were easily reduced to their nonyl derivatives in the reaction conditions required in our multistep synthetic scheme to obtain the desired TACAs [15]. Thus, our synthetic strategy was revised, and we converted the 9-bromononyl Glc*N*Ac glycoside to the 9-chlorononyl glycoside analog, which we knew would be stable throughout our synthetic scheme [15]. In turn, we investigated the displacement of the chlorine atom by bromide to further allow for its displacement by *N-*hydroxyphthalimide in mild conditions. Thus, we report below our attempts to displace the chloride of chloroalkyl β Gc*N*Ac glycosides using sodium bromide in a mixture of dimethylformamide (DMF) and dibromomethane (DBM) and our observation that these conditions promoted anomerization of the β glycosides to the α anomers.

## 2. Results

### 2.1. Substitution and Anomerization of Chloroalkyl β Glycosides

**Scheme 1:**
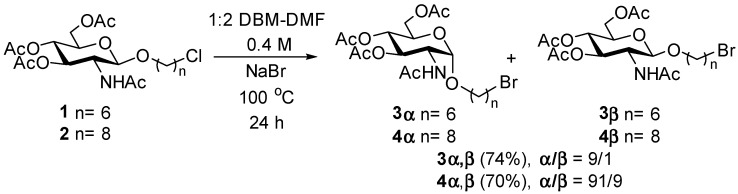
Displacement of chloride for bromide in chloroalkyl β glycosides **1** and **2**.

Our investigation into the phenomena began with studying the substitution of bromide for chloride in chloroalkyl glycosides with six- and eight-membered chains (Scheme 1). We first prepared (Supplementary Material) the known [16] 6-chlorohexyl β glycoside **1** and the 8-chlorohexyl β glycoside **2** from known [17] Horton’s chloride. The nucleophilic displacement of the chlorine atoms in **1** and **2** was then attempted using the conditions established by Babler and Spina [18]. In these conditions, the chloride displacement with NaBr is carried out in a 1:2 (*v*/*v*) ratio of DBM-DMF, in which the DBM facilitates the displacement by acting as a trap for chloride ions [18]. Thus, β glycosides **1** and **2** at a 0.4 M concentration were reacted in sealed flasks with NaBr (10 eq) in a 1:2 mixture of DBM and DMF at 100 °C for 24 h (Scheme 1).

Under these conditions, the displacement of the chloride by bromide was complete for both compounds **1** and **2,** giving, after work-up, the bromoalkyl glycosides **3** and **4** in 74 and 70% yields, respectively. This was confirmed by the shift of the methylene triplets from ~3.5 ppm in the ^1^H NMR spectra correlating to ~45 ppm in the ^13^C NMR spectra for the CH_2_Cl in compounds **1** and **2** to ~3.4 ppm in the ^1^H NMR spectra correlating at ~34 ppm in the ^13^C NMR spectra for the CH_2_Br in compounds **3** and **4**. However, careful examination of the Glc*N*Ac ring signals revealed the presence of two glycosides in both compounds **3** and **4**, which were faintly separated on TLC. In both compounds, we found that the α anomers **3α** and **4α** were the major products (α/β ≈ 9:1), characterized by anomeric signals appearing as doublets at ~4.8 ppm with the characteristic coupling constants ^3^*J*_H-1,H-2_ = 3.7 Hz in the ^1^H NMR spectra and that correlated to anomeric signals at 97.1 ppm in the ^13^C NMR spectra. In contrast, the minor β anomers **3β** and **4β** were characterized by anomeric signals appearing as doublets at ~4.6 ppm in the ^1^H NMR spectra with the characteristic coupling constants ^3^*J*_H-1,H-2_ = 8.3 Hz and that correlated to anomeric signals at 100.7 ppm in the ^13^C NMR spectra. After careful chromatography, analytical samples were obtained for all compounds, and their NMR spectra are given in the Supplementary Material.

This unexpected anomerization became the focus of our study. We examined the effect of the concentration and presence of sodium bromide in the reaction mixture at 100 °C and the effect of temperature on the substitution and anomerization of compound **1**. All reactions were allowed to proceed for 24 h in sealed flasks, and the results are listed in Table 1.

The degree of substitution of chloride by bromide and the anomeric ratios were estimated from the ^1^H NMR spectra of the crude products through the relative integrations of the methylene signals and the anomeric protons, respectively. As can be seen in Table 1, diluting the reaction from 0.4 M to 0.04 M had no impact on the substitution or anomerization reactions; we observed full displacement of the chloride and a 9/1 ratio of α/β anomers in both reactions (Table 1, entries 1 and 2). Interestingly, omitting sodium bromide from the reaction mixture had no impact on the degree of substitution and only a negligible effect on the degree of anomerization. Indeed, at 100 °C, the chlorine atom was again fully displaced, and the α/β ratio was 85/15 (Table 1, entry 3). Lowering the temperature only had a moderate impact on the degree of substitution of chloride by bromide; it was complete at 100 and 90 °C (entries 1 and 2) and only dropped down to a ratio 82/18 in favor of the bromoalkyl glycoside at 70 °C (entry 6). In contrast, lowering the temperature had a major impact on the degree of anomerization. At 90 °C, the anomerization occurred only for half of the β glycoside, leading to an α/β ratio of 1/1 (entry 4). At 80 °C, the glycoside remained mainly β, with a 15/85 α/β ratio, while at 70 °C, very little β glycoside anomerized to the α configuration, leading to an α/β ratio of 6/94. It is important to point out that given the remote position of the halogen from the ring signals, NMR does not allow for assigning chlorine or bromine substitution to a specific anomer; thus, we were unable at this point to conclude whether any of the chloroalkyl or bromoalkyl glycosides were anomerizing preferentially.

**Figure 1:**
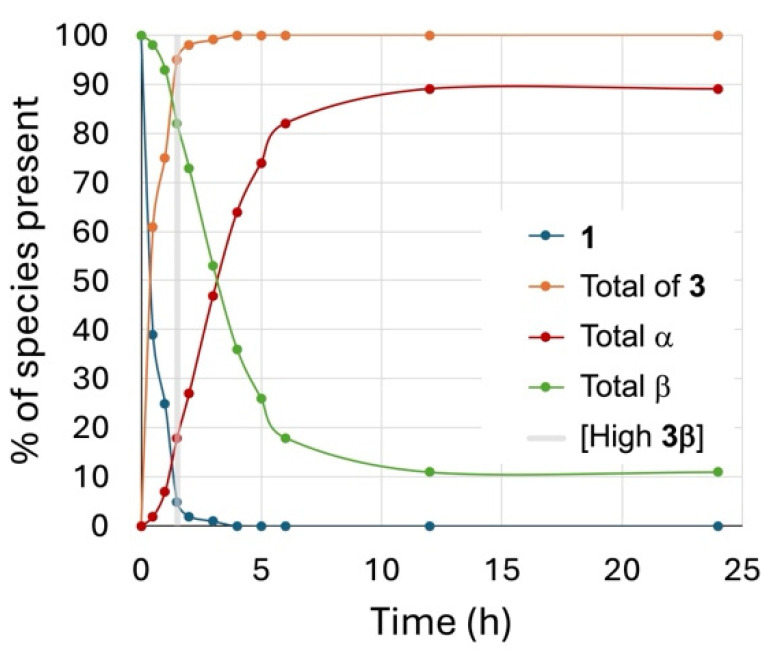
Tracked reaction of the conversion from **1** to **3** at 100 °C, 0.04 M in a 1:2 DBM-DMF mixture containing NaBr (10 equiv) in a sealed flask.

To observe the kinetics of product formation during the reaction, a time trial was conducted by reacting the 6-chlorohexyl β glycoside **1** (0.04 M) in a sealed flask at 100 °C in a 1:2 DBM-DMF mixture with NaBr (10 equiv). Samples were taken every 30 min for the first 2 h and then hourly until reaching 6 h of reaction and then at 12 h, and finally, the reaction was stopped after 24 h. These samples were worked-up, the ratios were determined using ^1^H NMR as described above, and the results plotted against time are shown in Figure 1. From the graph, it is clear that the chloride displacement happened quickly, i.e., within the first 2 h of reaction, to give the 6-bromoalkyl glycoside. In contrast, the anomerization happened slowly in the first hour of reaction and then faster in the next 6 h to reach a plateau after 12 h of reaction. As can be seen in this plot, it appeared that anomerization happened preferentially from the 6-bromohexyl β glycoside. Furthermore, this tracked reaction showed an optimal time to isolate the 6-bromohexyl β glycoside (**3β**) in high yields prior to anomerization.

### 2.2. Anomerization of Alkyl β Glycosides

**Scheme 2:**
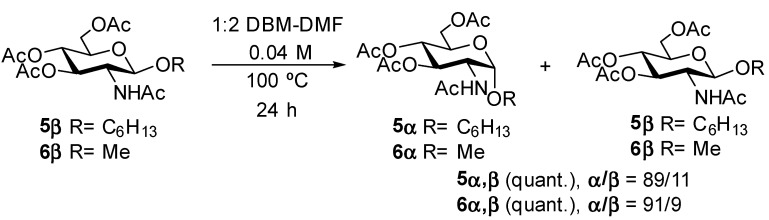
Anomerization of alkyl glycosides without halide displacement.

To assess whether the bromine atom in compounds **2** and **4** was involved in the anomerization reaction, we synthesized known [19] hexyl Glc*N*Ac β glycoside **5β** and known [20] methyl Glc*N*Ac β glycoside **6β** from known [17] Horton’s chloride (Supplementary Material). The β glycosides **5β** and **6β** were then submitted to the reaction conditions described above (1:2, DBM-DMF, 100 °C, sealed flasks, 24 h) omitting the sodium bromide (Scheme 2), which did not significantly impact anomerization.

After work-up, glycosides **5** and **6** were recovered in quantitative yields, and they were analyzed by NMR. To our surprise, we found for both compounds that the known α anomers **5α** [21] and **6α** [22] were again the major products of the reactions, with α/β ratios of ~9/1. Full assignment of the ^1^H and ^13^C NMR signals for the glycosides **5α** and **6α** in the mixtures were obtained by irradiating the anomeric signals in 1D TOCSY experiments and correlating these signals to carbon signals using an HSQC experiment (Supplementary Material). Thus, these α glycosides were characterized by anomeric signals appearing as doublets at 4.80 and 4.70 ppm with characteristic coupling constants ^3^*J*_H-1,H-2_ = 3.7 and 3.6 Hz in the ^1^H NMR spectra and that correlated to anomeric signals at 97.1 and 98.3 ppm in the ^13^C NMR spectra for compounds **5α** and **6α**, respectively. In contrast, the minor β glycosides were characterized by anomeric signals appearing as doublets at 4.66 and 4.56 ppm in the ^1^H NMR spectra with the characteristic coupling constants ^3^*J*_H-1,H-2_ = 8.3 Hz and that correlated to anomeric signals at 100.7 and 101.6 ppm in the ^13^C NMR spectra for compounds **5β** and **6β**, respectively. Attempts to anomerize the β glycosides **5β** and **6β** in either DBM or DMF alone at 100 °C for 24 h were unsuccessful. Adding sodium bromide to solutions of glycosides **5β** and **6β** in DBM or DMF at 100 °C did not lead either to anomerization. Thus, it appeared that the anomerization reaction only proceeded in a mixture of DBM and DMF.

**Figure 2:**
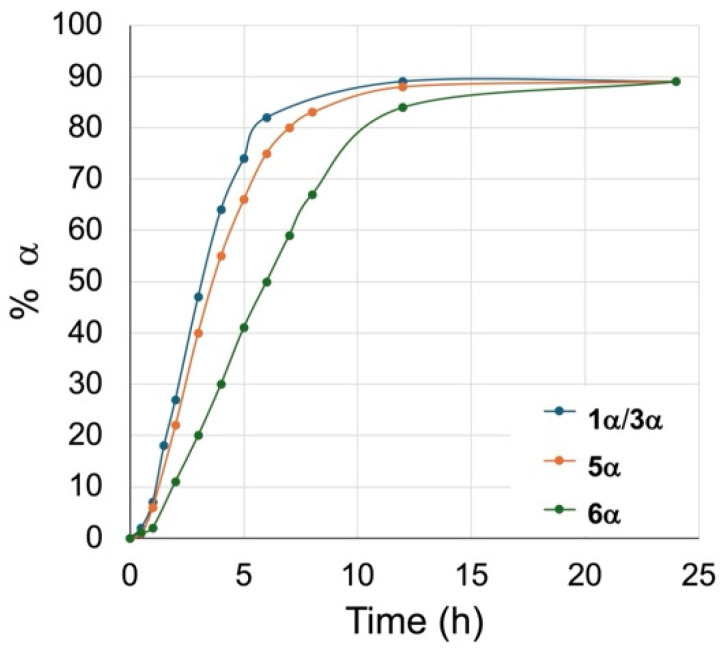
Comparison of reaction rates for the anomerization for compounds **1**/**3**, **5**, and **6,** 100 °C, 0.04 M, DBM-DMF (1:2). Assessed by ^1^H NMR.

Time trials were completed with the β glycosides **5β** and **6β** to track the formation of the α anomer over time. The % amount of α anomers **5α** and **6α** formed was assessed by relative integration of the anomeric signals in the ^1^H NMR. The results of these time trials are plotted against time in Figure 2 and compared to the anomerization of the 6-chlorohexyl β glycoside **1,** giving the 6-bromo/6-chlorohexyl glycosides **1/3α** shown in Figure 1.

All β glycosides anomerized to 90% over 24 h. Notably, the hexyl glycosides **1**/**3β** and **5β** anomerized faster than the methyl glycoside **6β**, with 74% and 66% of **1**/**3α** and **5α**, respectively, formed after 5 h, while the α methyl glycoside only reached 41% **6α** after the same length of time. Similarly to the anomerization of the 6-chlorohexyl β glycoside **1**, we also observed that anomerization of the β alkyl glycosides was slower in the first hour of reaction. In our study of the anomerization of the 6-chlorohexyl β glycoside (see above), we attributed this delay in the kinetics of anomerization to the initial displacement of the chloride by bromide, postulating that it was preferentially the 6-bromohexyl β glycoside **3β** that underwent anomerization. However, given that this delay was also observed with the alkyl β glycosides, the bromine in the 6-bromohexyl β glycoside **3β** had no significant impact on the anomerization.

**Figure 3:**
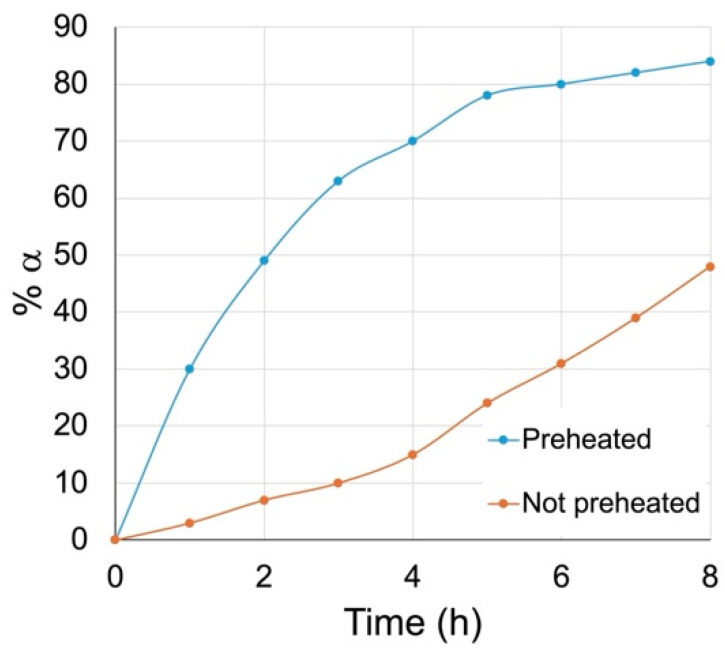
Anomerization of methyl β glycoside **6β** in DBM-DMF with (blue curve) or without (orange curve) preheating of the DBM-DMF.

We attempted two time trials in parallel (Figure 3). In one reaction, the 1:2 DBM-DMF mixture was preheated at 100 °C for 24 h in a sealed flask before the addition of the methyl β glycoside **6β** dissolved in a minimum amount of fresh solvent. The other reaction was carried out as usual; the methyl β glycoside **6β** was dissolved in DBM-DMF (1:2), and the solution was placed in a sealed flask at 100 °C. The amount of anomerization in each reaction was followed at regular intervals by taking aliquots, working them up, running ^1^H NMR, and integrating the anomeric signals. The percentage formation of the **6α** anomer in these reactions was plotted against time, and the results are shown in Figure 3. From this graph, it is clear that when the DBM-DMF mixture was preheated, the anomerization occurred readily, while it was slower when the solvents had not been preheated.

To assess the reactivity of DBM with DMF, the two solvents were stirred at a ratio 1:2 in a sealed flask at 100 °C in an oil bath for 24 h. After cooling the reaction mixture to room temperature, we observed the formation of a crystalline solid suggesting that the two solvents had reacted together. This hygroscopic solid was isolated by centrifugation, washed with diethyl ether, dried and added to a solution of methyl β glycoside **6β** in pure DMF. Heating the solution to 100 °C led to 4% anomerization after 24 h and 7% after 48 h. Although anomerization did not reach the levels observed in the DBM-DMF mixture, this was a significant indication that the solid was able to promote anomerization, since heating the β glycoside **6β** in pure DMF did not lead to any anomerization.

**Figure 4:**
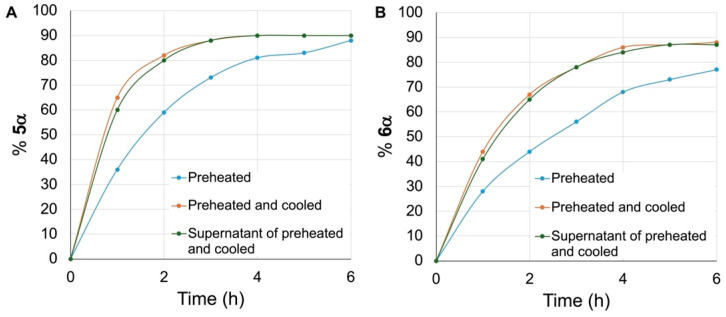
Anomerization of hexyl (**A**) and methyl (**B**) glycosides **5β** and **6β**. Blue curves: **5β** and **6β** were added at 100 °C to preheated (24 h) mixtures of DBM-DMF. Orange curves: **5β** and **6β** were added at room temperature to preheated (100 °C, 24 h)-then-cooled mixtures of DBM-DMF, and the reactions were then placed at 100 °C. Green curves: **5β** and **6β** were added at room temperature to the supernatant of a preheated (100 °C, 24 h)-then-cooled mixture of DBM-DMF, and the reactions were then placed at 100 °C.

Six additional time trials were conducted in parallel: three with the β hexyl glycoside **5β** (Figure 4A) and three with the β methyl glycoside **6β** (Figure 4B). For two of these, the mixtures of DBM-DMF (1:2) were heated at 100 °C for 24 h, and the glycosides were then added to the mixture without delay (Figure 4, blue curves) while the temperature was maintained at 100 °C. For two others, the mixtures of DBM-DMF (1:2) were heated at 100 °C for 24 h, and then cooled to room temperature as we observed the solid forming. The glycosides were then added to the cold mixtures, which were then placed again at 100 °C (Figure 4, orange curves). For the last two experiments, a mixture of DBM-DMF (1:2) was heated at 100 °C for 24 h, then cooled to room temperature, the solid was decanted, and the supernatant was retrieved and added to the glycosides. The mixtures were then placed at 100 °C (Figure 4, green curves). Aliquots of all reactions were taken at 1 h intervals, they were worked-up, the % anomerization giving **5α** and **6α** was assessed by NMR, and the results are given in Figure 4A and 4B, respectively. As can be seen from Figure 4, anomerization began without delay in all cases. Anomerization of the methyl glycoside **6β** was confirmed to be slower than that of the hexyl glycoside **5β** in all cases. For instance, after 2 h of reaction, the relative ratios of **5α** to **6α** were 60 to 44 (blue curve) and 80 to 66 (orange and green curves). Interestingly, heating the DBM-DMF mixtures (100 °C, 24 h) and then cooling them to room temperature before adding the glycosides and then placing the reactions at 100 °C led to faster anomerization of **5β** and **6β** (Figure 4, orange curves). Similarly, decanting the cooled supernatant of a preheated mixture of DBM-DMF (100 °C, 24 h) and adding it to the glycosides and then placing the mixtures at 100 °C also increased the kinetics of anomerization for both **5β** and **6β** (green curves), albeit a bit more slowly than when the β glycosides were added to the heated-then-cooled mixtures of DBM-DMF.

## 3. Discussion

We have discovered simple and cost-effective conditions to promote the anomerization of *N*-acetylglucosamine β glycosides to their α anomers with up to 90% conversion. These conditions involve dissolving the β glycosides in DBM-DMF (1:2) and placing the solutions at 100 °C for 24 h in a sealed flask. Our study suggests that heating the DBM-DMF mixture at 100 °C leads to the formation of a compound that promotes this anomerization. Upon cooling of a DBM-DMF (1:2) mixture heated at 100 °C for 24 h, we observed the formation of a crystalline solid. We have shown that this hygroscopic solid promoted the anomerization of methyl β glycoside **6β** in pure DMF to 4% after 24 h and 7% after 48 h. Since heating the β glycoside in pure DMF did not lead to any anomerization, this result suggests that this solid could have promoted the anomerization. We have shown that the anomerization was faster when the hexyl and methyl β glycosides (**5β** and **6β**) were stirred at 100 °C in the supernatant of a DBM-DMF mixture preheated at 100 °C for 24 h and then cooled to room temperature and decanted before being added to the β glycosides. Interestingly, cooling preheated (24 h, 100 °C) mixtures of DBM-DMF and then adding the β glycosides at room temperature before placing the mixtures at 100 °C further increased the kinetics of anomerization for both hexyl and methyl glycosides **5β** and **6β**. We propose that the formation of the crystalline solid during the heating/cooling cycle of the mixture prior to the addition of the β glycosides increased the concentration of the active species in solution, thereby enhancing the kinetics of anomerization.

**Scheme 3:**
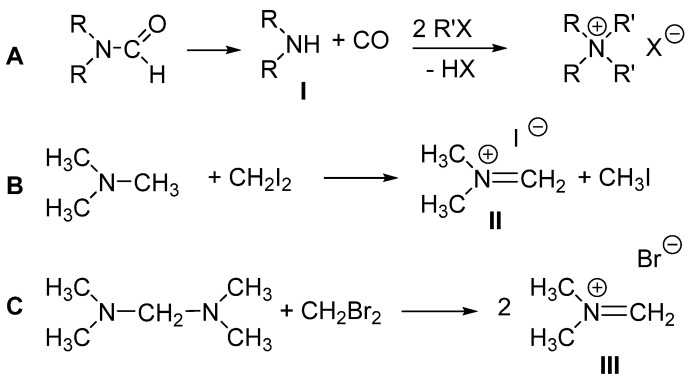
(**A**) Known [23,24] reaction of *N*,*N*-disubstituted amides with alkyl halides; (**B**) known [25] reaction of trimethylamine with diiodomethane; (**C**) known [26] reaction of TMMD with dibromomethane.

It is known [23] that DMF and methyl bromide heated to 80 °C in a sealed vessel lead to the formation tetramethylammonium bromide. Indeed, the reaction of *N*,*N*-disubstituted amides with alkyl halides is a known way to access various quaternary ammonium halide salts [24]. In these reactions (Scheme 3A), the amides first degrade to give carbon monoxide and the corresponding dialkylamines (**I**), which then undergo two successive nucleophilic displacements by alkyl halides, yielding the quaternary ammonium salts [24]. It is also known that the reaction of dihalomethane (CH_2_I_2_ or CH_2_Br_2_) with trimethylamine (Scheme 3B, [25]) or *N*,*N*,*N*′,*N*′-tetramethylmethylenediamine (TMMD) (Scheme 3C, [26]) produces dimethymethyleniminium salts (**II** and **III**).

**Scheme 4:**
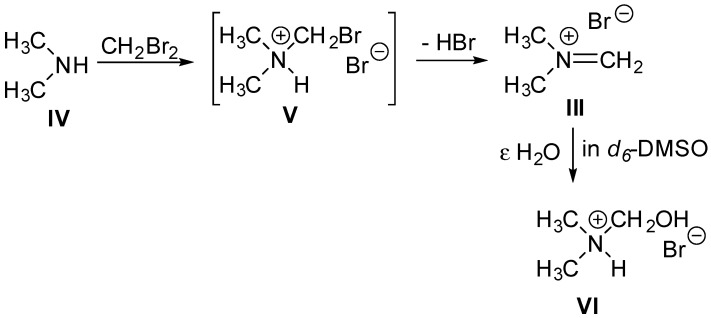
Proposed mechanism of formation of iminium salt **III** and hydroxymethyl(dimethyl)ammonium bromide **VI** observed in “wet” *d*_6_-DMSO.

We observed constant gas evolution while heating the sealed DBM-DMF reaction mixtures, which strongly suggested that DMF dissociated into dimethylamine and carbon monoxide. The ^1^H NMR (*d_6_*-DMSO) spectrum of the dried solid showed four products (Supplementary Material). The major compound was characterized by two signals of a 3-to-1 relative integration at 3.65 ppm and 8.18 ppm, correlating in the HSQC to methyl and methylene carbons at 48 and 168 ppm, respectively. These signals are in agreement with those reported for the iminium iodide salt (**II**, Scheme 3) described by Schreiber et al. [25] at 3.67 and 8.18 ppm, respectively. Further examination of the ^1^H NMR spectrum of the solid also revealed two signals of a 3-to-1 relative integration that, depending on the sample, appeared as doublets or broad singlets at 2.66 ppm and 4.44 ppm, both correlating in the COSY spectrum to a broad NH signal at 9.5 ppm. In the HSQC, these signals correlated to methyl and methylene carbons at 38 and 79 ppm, respectively. We propose that these signals correspond respectively to the two methyl and the hydroxymethyl substituents in hydroxymethyl(dimethyl)ammonium bromide (Scheme 4, **VI**), which was likely formed by the reaction of the iminium salt (**III**) with water contained in the NMR solvent. Finally, NMR of the solid in *d*_6_-DMSO also showed two unrelated signals: a doublet at 2.77 ppm and a triplet at 2.54 ppm, which correlated in the COSY spectrum to broad NH signals at 9.6 and 8.4 ppm, respectively. In the HSQC, these signals correlated to methyl carbons at 44 and 34 ppm, respectively. We propose that these signals correspond to dimethylamine (Scheme 4, **IV**) and dimethylammonium bromide, respectively.

**Figure 5:**
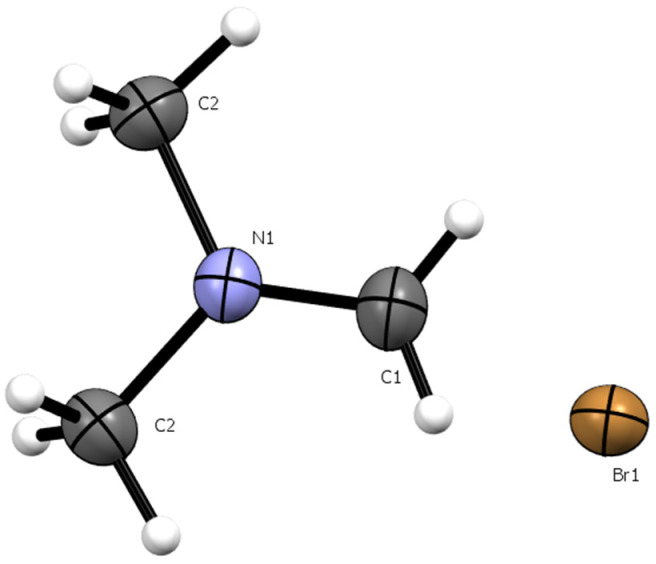
Crystal structure of dimethylmethyleniminium bromide salt **III** with thermal ellipsoids shown at 50% probability with non-H atoms labelled.

X-ray crystallography of the crystalline solid confirmed that it was indeed the dimethylmethyleniminium bromide salt **III** (Figure 5). Crystallographic data and experimental details are given in the Supplementary Material. The structure obtained was deposited at the Cambridge Crystallographic Data Centre (CCDC) under the number 2425977 and is in complete agreement with that previously reported by Clark et al. [26] for the same compound.

Concentration of the supernatant and ^1^H NMR (*d_6_*-DMSO) analysis (Supplementary Material) of the residue showed the presence of what we assume to be dimethylamine (doublet at 2.77 ppm) and dimethylammonium bromide (triplet at 2.54 ppm) as major compounds. We also observed the presence of what we proposed to be hydroxymethyl(dimethyl)ammonium bromide (**VI**), resulting from the hydration of the dimethylmethyleniminium bromide salt by water contained in DMSO and characterized by the two doublets mentioned above (2.66 ppm and 4.44 ppm).

We propose (Scheme 4) that the main reaction taking place between DMF and DBM results from the nucleophilic displacement of bromide in dibromomethane by the dimethylamine **IV** formed after decomposition of the DMF. This reaction leads to the bromomethyl(dimethyl)ammonium bromide intermediate **V,** which promptly loses HBr to give dimethylmethyleniminium bromide **III**. Thus, we conclude that anomerization could be promoted either by the HBr released during the formation of the dimethylmethyleniminium bromide **III** or by the highly reactive dimethylmethyleniminium bromide salt **III** itself. Without attempting to optimize the reaction, we observed up to 7% anomerization when adding dry dimethylmethyleniminium bromide **III** salt to a solution of the β glycoside **6β** in pure DMF and heating the solution at 100 °C for 48 h. Since stirring the β glycoside **6β** in pure DMF at 100 °C did not lead to any anomerization, this initial result strongly suggests that the highly reactive Mannich salt **III** is indeed capable of promoting anomerization. Further experiments are ongoing to confirm this hypothesis and clarify if the HBr formed is involved in the anomerization.

It has been known since the late 1920s that Lewis acids can promote the anomerization of β glucosides, as Pacsu [27,28,29] described the anomerization of methyl, hexyl, and cyclohexyl tetraacetyl β-d-glucopyranosides using SnCl_4_ or TiCl_4_. Subsequently, Ikemoto et al. [30] described the anomerization of various acylated β glycosides using FeCl_3_ at room temperature and reported that acetylated and benzoylated β glycosides yielded the best results. Interestingly when attempting the anomerization of peracetylated β methyl *N*-acetylglucosamine glycoside **6β**, they observed the formation of the oxazoline in a 32% yield, which suggested an exocyclic cleavage of the glycosidic bond. Expanding on these early results, the Murphy group studied the TiCl_4_- and SnCl_4_-promoted anomerization of various β glycosides [31,32,33,34]. These studies confirmed the early work by Pacsu that showed that TiCl_4_ was more efficient at promoting anomerization than SnCl_4_ [32]. The Murphy group discovered that β glucuronic acids were more easily anomerized than glycosides, 6-deoxy glycosides, or xylopyranosides and that fuco-, xylo-, arabino-, gluco-, and galactopyranosides, including β glycosides of *N*-acetylglucosamine, could be anomerized in these conditions [31,32,34]. Furthermore, they also studied the impact of the O-2 protecting group, showing that 2-*O*-acetylated β glycosides were less reactive toward anomerization than their 2-*O*-benzoylated analogues [33]. Interestingly, Crich and Vinod made the serendipitous discovery that a β methyl glycoside of 2-*N*-acetyl-2-*N*,3-*O*-oxazolidinone of glucosamine underwent anomerization to the α anomer when treated with NaCNBH_3_ and HCl in diethyl ether [35]. The Oscarson and Ito groups also independently reported the anomerization of *N*-acetyl or *N*-benzyl 2,3-*trans*-oxazolidinones of glucosamine promoted by AgOTf or BF_3_•Et_2_O, respectively [36,37]. Crich [35] suggested that the anomerization of these 2,3-oxazolidinones was assisted by the strain created by the 2,3-*trans*-fused ring, which facilitated a O5-C1 *endo* cleavage, eventually leading to the more stable α anomer. Indeed, subsequent calculations by the Ito group showed that the *N*-benzyl-2,3-*trans*-oxazolidinone group gave lower energies for the transition state and *endo* O5-C1 cleavage than pyranosides not carrying this 2,3-*trans*-fused ring [38]. More recently, Frem et al. [39] also reported anomerization of the n-butyl β glycoside of peracetylated Glc*N*Ac promoted by Cu(OTf)_2_ or triflic acid at 130 °C in 1,2-dichloroethane. In the latter case, they observed considerable degradation of the glycoside. They also reported transglycosylation of the n-butyl β glycoside to the methyl β glycoside when adding excess methanol to the reaction mixture. Interestingly, they observed limited anomerization of the β methyl glycoside, only reaching an α/β ratio of 1/6 in this reaction.

**Scheme 5:**
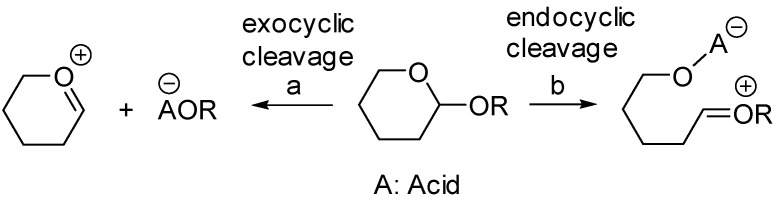
Possible path of glycosidic bond cleavage: a. exocyclic cleavage, b. endocyclic cleavage.

Glycosidic bonds can be cleaved either via an exocyclic C1-O1 or endocyclic O5-C1 cleavage (Scheme 5 (a and b)).

**Scheme 6:**
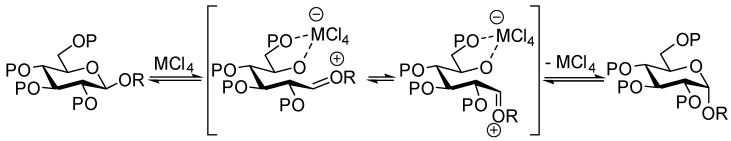
Proposed mechanism by Murphy [33] for the anomerization of β glucosides catalyzed by Lewis acids.

It is generally accepted that when submitted to solvolysis in protic conditions, α glycosides undergo exocyclic C1-O1 cleavage assisted by the stereoelectronic effect of the ring oxygen O-5 [40,41,42]. In contrast, the path of cleavage for β glycosides in protic acid has been shown to occur through both *exo* (C1-O1) and *endo* (O5-C1) cleavage [40,41,42]. Furthermore, the ring opening of β glycosides under Lewis acid catalysis such as Me_2_BBr [43], AlMe_3_ [44], and AlMe_3_/MeTiCl_3_ [45] was shown to proceed mostly through *endo* cleavage. In his studies, Murphy [33] proposed that anomerization of β glycosides promoted by TiCl_4_ or SnCl_4_ occurred via an endocyclic O5-C1 cleavage induced by the chelation of the O5 and O6 oxygens by the metal catalyst, as shown in Scheme 6.

We do not have unquestionable evidence that the anomerizations described here follow endocyclic opening at the O5-C1 bond. However, the fact that we do not observe either hemiacetal formation (even when adding traces of water to our reactions) or the formation of oxazoline as reported by Ikemoto et al. [30] strongly suggests that the glycosidic C1-O1 bond is not cleaved during the anomerization. Thus, our results suggest that the anomerizations follow an endocyclic mechanism.

As we have described above, while all β glycosides **1**, **2**, **5,** and **6** reached a 9/1 α/β ratio after 24 h of reaction, the β methyl glycoside **6β** was slower to anomerize that the β hexyl glycoside **5β** (Figure 2 and Figure 4), suggesting that the size of the glycoside impacts the reaction. These results are in agreement with the results reported by Pacsu [28], who observed faster anomerization of the peracetylated β hexyl glucopyranoside than that of its methyl glucoside analog under TiCl_4_ activation.

Further experiments are ongoing in our laboratory to confirm whether dimethylmethyleniminium bromide **III**, HBr, or the combination of both promotes anomerization. Furthermore, we are currently investigating the scope of this reaction and its applicability to other glycosides, especially those that do not carry an acetamido group at C-2. Preliminary results have shown that the presence of a more electron-withdrawing *N*-Troc, *N*-trichloroacetyl (*N*-TCA), or *O*-Ac had a significant impact on the degree of anomerization. These results will be reported in due course.

## 4. Materials and Methods

### 4.1. General Experimental

All chemicals were purchased from Fisher Scientific (Ottawa, ON, Canada) or Sigma Aldrich (Oakville, ON, Canada) and were used without purification. Anhydrous dichloromethane was obtained by distillation from phosphorus pentoxide. ^1^H NMR and ^13^C NMR spectra were recorded at 298 K on Bruker Avance-400 or 600 spectrometers (Bruker Biospin, Boston, MA, USA) for solutions in CDCl_3_ (calibrated at δ_C_ 77.0 ppm, using residual CHCl_3_ at δ_H_ 7.24 ppm) or *d_6_*-DMSO (calibrated at ^13^C δ_C_ 39.5 ppm, using residual CHD_2_SOCD_3_ at δ_H_ 2.50 ppm). All chemical shifts are reported in parts per million (ppm). All coupling constants (*J*) are reported in hertz (Hz) and were obtained from analysis of ^1^H NMR spectra. Structural assignments were made with additional information from gCOSY, gHSQC. 1D TOCSY experiments were acquired with mixing times of 150 ms and pre-scan delays of 2 s. Multiplicities are abbreviated as singlet (s), broad singlet (bs), doublet (d), doublet of a doublet (dd), triplet (t), and multiplet (m). Organic solutions were dried over Na_2_SO_4_ and concentrated under reduced pressure. Thin layer chromatography (TLC) was performed on aluminum plates coated with silica gel and charred with 20% H_2_SO_4_ in EtOH. Compounds were purified by flash column chromatography using Silica Gel 60 (230–400 mesh). Mass spectra were obtained under electrospray ionization (ESI) on a Thermo Scientific Orbitrap Velos Pro spectrometer equipped with a TOF analyzer at the Queen’s Mass Spectrometry Facility (Kingston, ON, Canada). The reported masses are for ^35^Cl isotope of chlorine and the ^79^Br isotope for bromine. Optical rotations for new compounds were measured on a Autopol^®^ IV automatic polarimeter by Rudolph Research Analytical (Hackettstown, NJ, USA).

### 4.2. General Method for the Anomerization Reactions

Glycosides **1**, **2**, **5β**, and **6β** (10–100 mg) were dissolved in a 1:2 volumetric ratio of DBM-DMF at concentrations of 0.04 M or 0.4 M. For glycosides **1** and **2**, 10 equiv of NaBr was added in all reactions except for that described in Table 1, entry 3. The reaction mixtures were sealed and placed in an oil bath preheated to the desired temperature (70–100 °C), and the reactions were left to proceed for 24 h. The solutions were diluted in 5 mL of DCM, washed with water (2 × 5 mL), and the water phases were re-extracted with DCM (3 × 5 mL). The combined organic layers were dried on Na_2_SO_4,_ concentrated under reduced pressure at 40 °C to yield tan syrups or amorphous solids. The products were analyzed by TLC (hexanes:EtOAc) and NMR.

### 4.3. 8-Chlorooctyl 2-Acetamido-3,4,6-tri-O-acetyl-2-deoxy-β-d-glucopyranoside (***2***)

Known [17] 2-acetamido-3,4,6-tri-*O*-acetyl-2-deoxy-β-d-glucopyranosyl chloride (Horton’s chloride, 4 g, 11 mmol) was dissolved in anhyd DCM (40 mL) under N_2_. Molecular sieves (4 Å, 5 g) were added along with 8-chloro-1-octanol (2.8 mL, 16 mmol, 1.5 equiv), and the reaction was stirred for 1 h. HgCl_2_ (3.86 g, 14 mmol, 1.3 equiv) was added to the reaction, which was left to stir for 16 h at rt. The mixture was diluted with DCM (50 mL), filtered over Celite^®^, and the filtrate was washed with satd aq NaHCO_3_ (2 × 50 mL) and water (3 × 50 mL), and the water phases were re-extracted with DCM (3 × 20 mL). The combined organic layers were dried over Na_2_SO_4_ and concentrated under reduced pressure. The pure 8-chlorooctyl glycoside **2** was precipitated in cold 1:1 Et_2_O–toluene and filtered off as a white solid (3.6 g, 67%). Chromatography of the mother liquor (EtOAc-hexanes, 8:2) gave additional pure 8-chlorooctyl glycoside **2** (0.32 g, 6%). [α]_D_ −8.80 (*c* 0.5, MeOH). ^1^H NMR (CDCl_3_, 400 MHz) *δ*_H_ 5.42 (d, 1H, *J* = 8.7 Hz, NH), 5.28 (dd, 1H, *J* = 9.3, 10.6 Hz, H-3), 5.04 (t, 1H, *J* = 9.7 Hz, H-4), 4.66 (d, 1H, *J* = 8.3 Hz, H-1), 4.24 (dd, 1H, *J* = 4.8, 11.9 Hz, H-6a), 4.11 (dd, 1H, *J* = 2.5, 12.2 Hz, H-6b), 3.86–3.75 (m, 2H, H-2, OCH*H*CH_2_), 3.69–3.65 (m, 1H, H-5), 3.51 (t, 2H, *J* = 6.7 Hz, CH_2_Cl), 3.47–3.41 (m, 1H, OC*H*HCH_2_), 2.06, 2.01, 2.00, 1.92 (4s, 12H, 4 × COCH_3_), 1.73 (m, 2H, *J* = 7.8 Hz, C*H*_2_CH_2_Cl), 1.56–1.50 (m, 2H, OCH_2_C*H*_2_), 1.41–1.35 (m, 2H, C*H*_2_CH_2_CH_2_Cl), 1.29–1.25 (m, 6H, CH_2_(C*H*_2_)_3_CH_2_). ^13^C NMR (CDCl_3_, 100 MHz) *δ*_C_ 170.9, 170.7, 170.0, 169.4 (C=O), 100.7 (C-1), 72.3 (C-4), 71.8 (C-5), 69.8 (O*C*H_2_CH_2_), 68.7 (C-3), 62.2 (C-6), 54.9 (C-2), 45.1 (CH_2_Cl), 32.6 (*C*H_2_CH_2_Cl), 29.4, 29.1, 28.8, 26.8, 25.7 (CH_2_(*C*H_2_)_5_CH_2_), 23.4, 20.8, 20.7, 20.6 (CO*C*H_3_). HRESIMS *m/z*: [M + H]^+^ calcd for C_22_H_37_ClNO_9_ 494.2157; found 494.2151.

### 4.4. 6-Bromohexyl 2-Acetamido-3,4,6-tri-O-acetyl-2-deoxy-α-d-glucopyranoside (***3α***) and 6-Bromohexyl 2-Acetamido-3,4,6-tri-O-acetyl-2-deoxy-β-d-glucopyranoside (***3β***)

Known [16] 6-chlorohexyl 2-acetamido-3,4,6-tri-*O*-acetyl-2-deoxy-β-d-glucopyranoside **1** (Supplementary Material, 100 mg, 0.215 mmol) was reacted in DBM-DMF (1:2) at a 0.4 M concentration with 10 equiv of NaBr in a sealed flask as described above in the general method (100 °C, 24 h). Work-up as described above gave a brown syrup (82 mg, 74%). The α/β (9:1) ratio was determined by NMR. Purification of the residue with chromatography (EtOAc-hexanes, 7:3) gave analytical samples of pure **3α** and **3β**.

Analytical data for compound **3α**: [α]_D_ +73.1 (*c* 1.0, MeOH). ^1^H NMR (CDCl_3_, 400 MHz) δ_H_ 5.64 (d, 1H, *J* = 9.4 Hz, NH), 5.18 (dd, 1H, *J* = 9.3, 10.5 Hz, H-3), 5.09 (t, 1H, *J* = 9.6 Hz, H-4), 4.81 (d, 1H, *J* = 3.7 Hz, H-1), 4.31 (m, 1H, H-2), 4.21 (dd, *J* = 4.8, 11.9 Hz, H-6a), 4.07 (dd, 1H, *J* = 2.5, 12.2 Hz, H-6b), 3.93–3.89 (m, 1H, H-5), 3.69–3.63 (m, 1H, OC*H*HCH_2_), 3.44–3.38 (m, 3H, CH_2_Br, OCH*H*CH_2_), 2.08, 2.01, 2.00, 1.92 (4s, 12H, 4 × COCH_3_), 1.86 (m, 2H, C*H*_2_CH_2_Br), 1.65–1.58 (m, 2H, OCH_2_C*H*_2_), 1.51–1.43 (m, 2H, C*H*_2_CH_2_CH_2_Br), 1.40–1.33 (m, 2H, OCH_2_CH_2_C*H*_2_). ^13^C NMR (CDCl_3_, 100 MHz) *δ*_C_ 171.4, 170.7, 169.8, 169.3 (C=O), 97.1 (C-1), 71.4 (C-4), 68.2 (OCH_2_), 68.1 (C-5), 67.7 (C-3), 62.0 (C-6), 51.9 (C-2), 33.7 (CH_2_Br), 32.5, 29.1, 27.8, 25.3 (CH_2_*(C*H_2_)_4_CH_2_Br), 23.2, 20.7, 20.7, 20.6 (CO*C*H_3_). HRESIMS *m/z*: [M + H]^+^ calcd for C_20_H_33_BrNO_9_ 510.1339; found 510.1323.

Analytical data for compound **3β**: [α]_D_ −11.1 (*c* 0.54, MeOH). ^1^H NMR (CDCl_3_, 400 MHz) δ_H_ 5.44 (d, 1H, *J* = 8.9 Hz, NH), 5.27 (dd, 1H, *J* = 9.3, 10.6 Hz, H-3), 5.04 (t, 1H, *J* = 9.6 Hz, H-4), 4.65 (d, 1H, *J* = 8.3 Hz, H-1), 4.23 (dd, 1H, *J* = 4.8, 11.9 Hz, H-6a), 4.10 (dd, 1H, *J* = 2.5, 12.2 Hz, H-6b), 3.87–3.75 (m, 2H, H-2, OC*H*HCH_2_), 3.69–3.65 (m, 1H, H-5), 3.48–3.42 (m, 1H, OCH*H*CH_2_), 3.38 (t, 2H, *J* = 6.7 Hz, CH_2_Br), 2.09, 2.03, 2.02, 1.93 (4s, 12H, 4 × COCH_3_), 1.82 (m, 2H, C*H*_2_CH_2_Br), 1.63–1.52 (m, 2H, OCH_2_C*H*_2_), 1.47–1.28 (m, 4H, CH_2_(C*H*_2_)_2_CH_2_). ^13^C NMR (CDCl_3_, 100 MHz) δ_C_ 170.9, 170.7, 170.1, 169.4 (C=O), 100.7 (C-1), 72.3 (C-4), 71.8 (C-5), 69.6 (OCH_2_), 68.6 (C-3), 62.1 (C-6), 54.9 (C-2), 33.8 (CH_2_Br), 32.6, 29.2, 27.7 25.0 (CH_2_*(C*H_2_)_4_CH_2_Br), 23.4, 20.7, 20.7, 20.6 (CO*C*H_3_). HRESIMS *m/z*: [M + H]^+^ calcd for C_20_H_33_BrNO_9_ 510.1339; found 510.1339.

### 4.5. 8-Bromooctyl 2-Acetamido-3,4,6-tri-O-acetyl-2-deoxy-α-d-glucopyranoside (***4α***) and 8-Bromooctyl 2-Acetamido-3,4,6-tri-O-acetyl-2-deoxy-β-d-glucopyranoside (***4β***)

The 8-chlorooctyl glycoside **2** (105 mg, 0.213 mmol) was reacted in DBM-DMF (1:2) at a 0.4 M concentration with 10 equiv of NaBr in a sealed flask as described above in the general method (100 °C, 24 h). Work-up as described above gave a brown syrup (80 mg, 70%. The α/β (91:9) ratio was determined by NMR. Analytical samples of **4α** and **4β** were obtained by chromatography (EtOAc/hexanes, 7:3).

Analytical data for compound **4α**: [α]_D_ +49.0 (*c* 0.69, MeOH). ^1^H NMR (CDCl_3_, 400 MHz) δ_H_ 5.64 (d, 1H, *J* = 8.7 Hz, NH), 5.18 (dd, 1H, *J* = 9.3, 10.5 Hz, H-3), 5.09 (t, 1H, *J* = 9.6, 10.5 Hz, H-4), 4.80 (d, 1H, *J* = 3.7 Hz, H-1), 4.34–4.28 (m, 1H, H-2), 4.21 (dd, *J* = 4.6, 12.3 Hz, H-6a), 4.07 (dd, 1H, *J* = 2.5, 12.3 Hz, H-6b), 3.93–3.89 (m, 1H, H-5), 3.68–3.62 (m, 2H, H-2, OC*H*HCH_2_), 3.43–3.37 (m, 3H, OCH*H*CH_2_, CH_2_Br), 2.07, 2.01, 2.00, 1.93 (4s, 12H, 4 × COC*H*_3_), 1.84 (m, 2H, C*H*_2_CH_2_Br), 1.61–1.55 (m, 2H, OCH_2_C*H*_2_), 1.46–1.39 (m, 2H, C*H*_2_CH_2_CH_2_Br), 1.37–1.28 (m, 6H, CH_2_(C*H*_2_)_3_CH_2_). ^13^C NMR (CDCl_3_, 100 MHz) δ_C_ 171.4, 170.7, 169.8, 169.3 (C=O), 97.1 (C-1), 71.4 (C-4), 68.5 (O*C*H_2_), 68.1 (C-5), 67.7 (C-3), 62.0 (C-6), 51.9 (C-2), 33.9 (CH_2_Br), 32.7 (*C*H_2_CH_2_Br), 29.2, 29.1, 28.6, 28.0, 26.0 (CH_2_(*C*H_2_)_5_CH_2_CH_2_Br), 23.2, 20.7, 20.7, 20.6 (COCH_3_). HRESIMS *m/z*: [M + H]^+^ calcd for C_22_H_37_BrNO_9_ 538.1652; found 538.1646.

Analytical data for compound **4β**: [α]_D_ -12.1 (*c* 0.69, MeOH). ^1^H NMR (CDCl_3_, 400 MHz) δ_H_ 5.42 (d, 1H, *J* = 8.7Hz, NH), 5.28 (dd, 1H, *J* = 9.3, 10.6 Hz, H-3), 5.04 (t, 1H, *J* = 9.7 Hz, H-4), 4.66 (d, 1H, *J* = 8.3 Hz, H-1), 4.24 (dd, 1H, *J* = 4.8, 12.2 Hz, H-6a), 4.11 (dd, 1H, *J* = 2.5, 12.2 Hz, H-6b), 3.86–3.75 (m, 2H, H-2, OC*H*HCH_2_), 3.69–3.65 (m, 1H, H-5), 3.47–3.41 (m, 1H, OCH*H*CH_2_), 3.38 (t, 2H, *J* = 6.8 Hz, CH_2_Br), 2.06, 2.01, 2.00, 1.92 (4s, 12H, 4 × COC*H*_3_), 1.82 (m, 2H, C*H*_2_CH_2_Br), 1.57–1.50 (m, 2H, OCH_2_C*H*_2_), 1.43–1.35 (m, 2H, C*H*_2_CH_2_CH_2_Br), 1.31–1.23 (m, 6H, CH_2_(C*H*_2_)_3_CH_2_). ^13^C NMR (CDCl_3_, 100 MHz) δ_C_ 170.9, 170.7, 170.0, 169.4 (C=O), 100.7 (C-1), 72.3 (C-4), 71.8 (C-5), 69.8 (OCH_2_), 68.7(C-3), 62.2 (C-6), 54.9 (C-2), 34.0 (CH_2_Br), 32.7 (*C*H_2_CH_2_Br), 29.4, 29.1, 28.7, 28.0, 25.7 (CH_2_(*C*H_2_)_5_CH_2_CH_2_Br), 23.4, 20.8, 20.7, 20.6 (COCH_3_). HRESIMS *m/z*: [M + H]^+^ calcd for C_22_H_37_BrNO_9_ 538.1652; found 538.1652.

### 4.6. Hexyl 2-Acetamido-3,4,6-tri-O-acetyl-2-deoxy-α-d-glucopyranoside (***5α***) and Hexyl 2-Acetamido-3,4,6-tri-O-acetyl-2-deoxy-β-d-glucopyranoside (***5β***)

The known [19] hexyl glycoside **5β** (Supplementary Material, 12 mg, 0.027 mmol) was reacted in DBM-DMF (1:2) at a 0.04 M concentration in a sealed flask as described above in the general method (100 °C, 24 h). Work-up as described above gave a brown syrup (12 mg, quant). The α/β (89:11) ratio was determined by NMR.

NMR data for known major compound **5α** are in agreement with those previously reported [21] and were assigned in the mixture using 1D TOCSY (irradiation of H-1) and HSQC experiments (Supplementary Material): ^1^H NMR (CDCl_3_, 400 MHz) δ_H_ 5.63 (d, 1H, *J* = 9.5 Hz, NH), 5.19 (dd, 1H, *J* = 9.6, 10.6 Hz, H-3), 5.09 (t, 1H, *J* = 9.6 Hz, H-4), 4.80 (d, 1H, *J* = 3.7 Hz, H-1), 4.31 (m, 1H, H-2), 4.21 (dd, 1H, *J* = 4.8, 12.3 Hz, H-6a), 4.07 (dd, 1H, *J* = 2.5, 12.1 Hz, H-6b), 3.91 (m, 1H, H-5), 3.68–3.63 (m, 1H, OCH*H*CH_2_), 3.43–3.38 (m, 1H, OC*H*HCH_2_), 2.07, 2.01, 2.00, 1.93 (4 s, 12H, 4 × COCH_3_), 1.59 (m, 2H, OCH_2_C*H*_2_), 1.36–1.21 (m, 6H, CH_2_(C*H*_2_)_3_CH_3_), 0.88 (m, 3H, (CH_2_)_5_C*H*_3_). ^13^C NMR (CDCl_3_, 100 MHz) δ_C_ 171.4, 170.7, 169.8, 169.3 (C=O), 97.1 (C-1), 71.5 (C-4), 68.7 (O*C*H_2_CH_2_), 68.6 (C-5), 67.7 (C-3) 62.1 (C-6), 51.9 (C-2), 31.5 (OCH_2_*C*H_2_), 29.2, 25.8 (CH_2_(*C*H_2_)_2_CH_2_), 23.3 (COCH_3_), 22.6 (*C*H_2_CH_3_), 20.7, 20.7, 20.6 (CO*C*H_3_), 14.0 (CH_2_*C*H_3_).

NMR data for known minor compound **5β** are in agreement with those previously reported [19]: ^1^H NMR (CDCl_3_, 400 MHz) δ_H_ 5.46 (d, 1H, *J* = 8.7 Hz, NH), 5.29 (dd, 1H, *J* = 9.4, 10.5 Hz, H-3), 5.04 (t, 1H, *J* = 9.6 Hz, H-4), 4.66 (d, 1H, *J* = 8.3 Hz, H-1), 4.23 (dd, 1H, *J* = 4.7, 12.2 Hz, H-6a), 4.10 (dd, 1H, *J* = 2.4, 12.2 Hz, H-6b), 3.96–3.75 (m, 2H, H-2, OCH*H*CH_2_), 3.69–3.65 (m, 1H, H-5), 3.48–3.41 (m, 1H, OC*H*HCH_2_), 2.10, 2.02, 2.01, 1.92 (4 s, 12H, 4 × COCH_3_), 1.55 (m, 2H, OCH_2_C*H*_2_), 1.31–1.20 (m, 6H, CH_2_(C*H*_2_)_3_CH_3_), 0.85 (m, 3H, (CH_2_)_5_C*H*_3_). ^13^C NMR (CDCl_3_, 100 MHz) δ_C_ 170.9, 170.7, 170.1, 169.4 (C=O), 100.7 (C-1), 72.3 (C-4), 71.7 (C-5), 70.0 (O*C*H_2_CH_2_), 68.7 (C-3) 62.2 (C-6), 54.9 (C-2), 31.5 (OCH_2_*C*H_2_), 29.4, 25.5, (CH_2_(*C*H_2_)_2_CH_2_), 23.3 (CO*C*H_3_), 22.6 (*C*H_2_CH_3_), 20.7, 20.7, 20.6 (CO*C*H_3_), 14.0 (CH_2_*C*H_3_).

### 4.7. Methyl 2-Acetamido-3,4,6-tri-O-acetyl-2-deoxy-α-d-glucopyranoside (***6α***) and Methyl 2-acetamido-3,4,6-tri-O-acetyl-2-deoxy-β-d-glucopyranoside (***6β***)

The known [20] methyl glycoside **6β** (Supplementary Material, 10 mg, 0.028 mmol) was reacted in DBM-DMF (1:2) at a 0.04 M concentration in a sealed flask as described above in the general method (100 °C, 24 h). Work-up as described above gave a brown syrup (10 mg, quant.). The α/β (91:9) ratio was determined by NMR.

NMR data for known major compound **6α** are in agreement with those previously reported [22] and were assigned using 1D TOCSY (irradiation of H-1) and HSQC experiments (Supplementary Material): ^1^H NMR (CDCl_3_, 400 MHz) δ_H_ 5.69 (d, 1H, *J* = 9.7 Hz, NH), 5.19 (dd, 1H, *J* = 9.6, 10.6 Hz, H-3), 5.09 (t, 1H, *J* = 9.8 Hz, H-4), 4.70 (d, 1H, *J* = 3.6 Hz, H-1), 4.32 (m, 1H, H-2), 4.21 (dd, 1H, *J* = 3.7, 9.7 Hz, H-6a), 4.08 (dd, 1H, *J* = 3.6, 9.6 Hz, H-6b), 3.89 (m, 1H, H-5), 3.38 (s, 3H, OCH_3_), 2.07, 2.00, 1.99, 1.93 (4 s, 12H, 4 × COCH_3_). ^13^C NMR (CDCl_3_, 100 MHz) δ_C_ 171.4, 170.7, 169.9, 169.3 (C=O), 98.3 (C-1), 71.3 (C-4), 68.1 (C-5), 67.6 (C-3), 62.0 (C-6), 55.4 (CH_3_), 54.6 (C-2), 23.2, 22.7, 20.7, 20.6 (CO*C*H_3_).

NMR data for known minor compound **6β** are in agreement with those previously reported [20]: ^1^H NMR (CDCl_3_, 400 MHz) δ_H_ 5.45 (d, 1H, *J* = 8.9 Hz, NH), 5.25 (dd, 1H, *J* = 9.3, 10.4 Hz, H-3), 5.06 (t, 1H, *J* = 9.8 Hz, H-4), 4.56 (d, 1H, *J* = 8.3 Hz, H-1), 4.26 (dd, 1H, *J* = 4.7, 12.3 Hz, H-6a), 4.13 (dd, 1H, *J* = 2.5, 12.3 Hz, H-6b), 3.84 (m, 1H, H-2), 3.68 (m, 1H, H-5), 3.48 (s, 3H, OCH_3_), 2.08, 2.01, 2.00, 1.94 (4 s, 12H, 4 × COCH_3_). ^13^C NMR (CDCl_3_, 100 MHz) δ_C_ 171.0, 170.7, 170.2, 169.4 (C=O), 101.6 (C-1), 72.4 (C-4), 71.8 (C-5), 68.5 (C-3), 62.1 (C-6), 56.8 (*C*H_3_), 54.6 (C-2), 23.4, 20.7, 20.7, 20.6 (CO*C*H_3_).

## 5. Conclusions

We have described the synthesis of a new 8-chlorooctyl β Glc*N*Ac glycoside. We have established that the chlorine atom in this compound as well as in the 6-chlorohexyl analog can be swiftly displaced by bromide in a mixture of DBM and DMF at 90–100 °C with or without the addition of sodium bromide. However, these reactions must be carefully monitored to maximize the nucleophilic displacement while limiting anomerization of the glycosidic bond. At 100 °C, the reaction is best stopped after ~2 h. The bromide in these glycosides should then be easily displaced with *N-*hydroxyphthalimide in mild conditions [14], leading, after deprotection, to the corresponding aminooxy functional group needed for conjugation to PS A1 [10,11,12,13] or other carrier molecules.

In the course of this work, we discovered that these mild conditions led to the anomerization of the β glycosidic bond in Glc*N*Ac to the corresponding α anomer. At 100 °C, the α/β was 9/1 after 24 h of reaction in a mixture (1:2) of DBM and DMF. While we initially assumed that anomerization happened preferentially from the bromoalkyl glycosides, we established that the alkyl glycosides also anomerized in these conditions. We discovered that applying a heating–cooling cycle to the solvent mixtures prior to adding the β glycosides and placing the reaction at 100 °C increased the kinetics of anomerization. Upon cooling the preheated DBM-DMF mixtures, we observed the formation of a crystalline solid, which was characterized by NMR spectroscopy and X-ray crystallography to be the known [25,26] Mannich salt dimethylmethyleniminium bromide. Although we only observed limited anomerization (4–7%) when the solid was reacted with the β glycoside **6β** in pure DMF at 100 °C, we propose that this highly reactive carbocation could play the role of a Lewis acid in promoting anomerization in these reactions. However, it is also possible that the HBr formed together with the dimethylmethyleniminium bromide plays a role in the anomerization. Further experiments are ongoing in our laboratory to clarify the mechanism of this reaction. Our studies strongly suggest that anomerization of these Glc*N*Ac β glycosides occurs via an *endo* O5-C1 cleavage, followed by bond rotation and cyclization to the more stable α anomer. We have also shown that the nature of the aglycon impacted the rate of anomerization, with hexyl β glycosides of Glc*N*Ac anomerizing at a faster rate than the analogous methyl β glycoside. The conditions discovered and described here are cost-effective and simple when compared to using heavy-metal-containing Lewis acids such as TiCl_4_ or SnCl_4_ [27,28,29,31,32,33,34] to promote the anomerization of β glycosides. Furthermore, these conditions are mild and do not lead to the degradation of the glycoside like the use of a strong protic acid such as TfOH does [39]. In the present work, we focused our investigation on the anomerization of β glycosides of peracetylated *N*-acetylated glucosamine. Following this serendipitous discovery, additional experiments are ongoing to optimize the reaction conditions and further maximize the anomerization. Furthermore, we are currently investigating the scope of this reaction and its applicability to other glycosides, including disaccharides. Preliminary results suggest that the presence of a more electron-withdrawing *N*-Troc, *N*-TCA, or *O*-Ac at C-2 of a β-d-glucopyranoside has a significant impact on the degree of anomerization. Finally, further mechanistic studies are also ongoing to clarify the involvement of the Mannich salt dimethylmethyleniminium bromide and HBr in the anomerization; our results will be reported in due course.

## Figures and Tables

**Table 1:** Effect of the temperature, concentration, and presence of NaBr (10 equiv) on the substitution and anomerization of the 6-chlorohexyl β glycoside **1** (ratios assessed by ^1^H NMR).

Entry	Concentration (M)	Temperature (°C)	3α/3β	Br/Cl	Crude Yield (%)
1	0.4	100	90/10	100/0	74
2	0.04	100	90/10	100/0	78
3 ^a^	0.04	100	85/15	100/0	quant.
4	0.4	90	50/50	100/0	quant.
5	0.4	80	15/85	96/4	90
6	0.4	70	6/94	82/18	98

^a^ Reaction without NaBr.

## Data Availability

Data are contained within the article and Supplementary Materials.

## Supplementary Materials

The following supporting information can be downloaded at: https://www.mdpi.com/article/10.3390/molecules30071483/s1, Experimental section for the synthesis of known compounds: **1**, **5β**, and **6β**. X-ray crystallography of dimethylmethyleniminium bromide (**III**). Tables of crystallographic parameters for dimethylmethyleniminium bromide (**III**). ^1^H, JMOD, COSY, HSQC, and NMR spectra for new compounds: **2**, **3α**, **3β**, **4α**, and **4β**. ^1^H, JMOD, COSY, HSQC, and NMR spectra for known compounds: **1**, **5β**, and **6β**. ^1^H, JMOD, COSY, HSQC, and TOCSY for end mixtures for compounds **5α/β** (9/1) and **6α/β** (9/1). ^1^H, JMOD, COSY, and HSQC of dried solids formed upon heating/cooling of DBM-DMF (1:2) mixture. ^1^H, JMOD, COSY, and HSQC of supernatant from heated/cooled DBM-DMF (1:2) mixture. Refs. [46,47,48,49,50,51] are cited in the Supplementary Materials.

## Abbreviations

The following abbreviations are used in this manuscript:

TACA	Tumor-associated carbohydrate antigen
DMF	Dimethylformamide
DBM	Dibromomethane
Glc*N*Ac	*N*-acetyl glucosamine
TMMD	*N*,*N*,*N*′,*N*′-tetramethylmethylenediamine
