# Supplementary material for: Anomerization of N-Acetylglucosamine Glycosides Promoted by Dibromomethane and Dimethylformamide
Source: Molecules. 2025 Mar 27;30(7):1483. doi: 10.3390/molecules30071483 (PMC11990195; doi:10.3390/molecules30071483)

Anomerisation of *N*-acetylglucosamine glycosides promoted by dibromomethane and dimethylformamide

Natalie B. Condino 1, Doriane Rousseau 2,†, Esperance Mutoni 3,†, Jeffrey Davidson 4,†, Lara K. Watanabe 1 and France-Isabelle Auzanneau 1,*

1 Department of Chemistry, University of Guelph, Guelph, ON N1G 2W1, Canada;

ncondino@uoguelph.ca (N.B.C.); lwatanab@uoguelph.ca (L.K.W.)

2 Fareva Valdepharm, Parc Industriel d'Incarville CS 10606, 27106 Val de Reuil, France;

doriane.rousseau04@gmail.com

3 Sun Pharma Canada Inc., 126 East Drive, Brampton, ON L6T 1C1, Canada;

esperance_mutoni@outlook.com

4 Toronto Research Chemicals, 20 Martin Ross Avenue, Toronto, ON M3J 2 K8, Canada;

jeffrey.davidson@lgcgroup.com

* Correspondence: fauzanne@uoguelph.ca

† The work was entirely done at the University of Guelph.

Supplementary Material

	Pages
Experimental section for the synthesis of known compounds: **1, 5β, 6β**	2-3
X-ray Crystallography of dimethylmethyleniminium bromide (**III**):	3-4
Tables of crystallographic parameters for dimethylmethyleniminium bromide (**III**)	5-10
References	11
^{1}H, JMOD, COSY, HSQC for new compounds: **2**, **3α**, **3β**, **4α**, **4β**	12-31
^{1}H, JMOD, COSY, HSQC for known compounds **1**, **5β**, **6β**	32-43
^{1}H, JMOD, COSY, HSQC, TOCSY for end mixtures for compounds **5α/β** (9:1), **6α/β** (9:1)	44-53
^{1}H, JMOD, COSY, HSQC of isolated solids upon heating/cooling of DBM-DMF (1:2) mixture	54-58
^{1}H, JMOD, COSY, HSQC of an isolated supernatant upon heating/cooling of DBM-DMF (1:2) mixture	59-63

1. Experimental section for the synthesis of known compounds: 1, 5β, 6β

1.1. 6-Chlorohexyl 2-acetamido-3,4,6-tri-O-acetyl-2-deoxy-β-D-glucopyranoside (1).

Known [1] Horton's chloride (4 g, 11 mmol) was dissolved in dry DCM (50 mL) under N_2. Molecular sieves (4 Å, 5 g) were added along with 6-chlorohexanol (16 mmol, 1.5 eq) and the reaction was stirred for 1 h. $HgCl_2$ (3.87 g, 14 mmol, 1.3 equiv) was added to the reaction which was left to stir for 16 h at rt. The mixture was diluted with DCM (50 mL), filtered over Celite® and the filtrate was washed with satd aq $NaHCO_3$ (2 x 50 mL), water (3 x 50 mL), and the water phases were reextracted with DCM (3 x 20 mL). The combined organic layers were dried over Na_2SO_4 and concentrated under reduced pressure. The pure hexyl glycoside **1** was precipitated in cold 1:1 Et_2O:toluene and filtered off as a white solid (3.7 g, 73%). Chromatography of the mother liquor (EtOAc-hexanes, 8:2) gave additional pure 6-chlorohexyl glycoside **1** (0.03 g, 1%). NMR in agreement with literature. ^1H NMR ($CDCl_3$, 400 MHz) δ_H 5.48 (d, 1H, *J* = 8.7 Hz, NH), 5.28 (dd, 1H, *J* = 9.3, 10.6 Hz, H-3), 5.04 (t, 1H, *J* = 9.5 Hz, H-4), 4.66 (d, 1 H, *J* = 8.3 Hz, H-1), 4.24 (dd, 1H, *J* = 4.8, 12.2 Hz, H-6a), 4.11 (dd, 1H, *J* = 2.5, 12.2 Hz, H-6b), 3.82 (m, 2H, H-2, OCH*H*CH$_2$), 3.70-3.64 (m, 1H, H-5), 3.52 (m, 3H, C*H*$_2$Cl, OC*H*HCH$_2$), 2.06, 2.01, 2.00, 1.92 (4s, 12H, 4 × COCH$_3$), 1.74 (m, 2H, C*H*$_2$CH$_2$Cl), 1.56 (m, 2H, OCH$_2$C*H*$_2$), 1.46-1.28 (m, 4H, CH$_2$(C*H*$_2$)$_2$CH$_2$). ^{13}C NMR ($CDCl_3$, 100 MHz) δ_C 170.9, 170.7, 170.1, 169.4 (C=O), 100.7 (C-1), 72.3 (C-4), 71.8 (C-5), 69.6 (OCH$_2$CH$_2$), 68.6 (C-3) 62.1 (C-6), 54.9 (C-2), 45.0 (CH$_2$Cl), 32.4 (CH$_2$CH$_2$Cl), 29.2, 26.5, 25.1 (CH$_2$(CH$_2$)$_3$CH$_2$), 23.3, 20.8, 20.7, 20.6 (COCH$_3$).

1.2. Hexyl 2-acetamido-3,4,6-tri-O-acetyl-2-deoxy-β-D-glucopyranoside (5β)

Known [1] 2-acetamido-3,4,6-tri-*O*-acetyl-2-deoxy-β-D-glucopyranosyl chloride (0.99 g, 2.6 mmol) was dissolved in dry DCM (20 mL) under N_2. Molecular sieves (4 Å, 2 g) were added along with 1-hexanol (1.6 mL, 13 mmol, 5 eq) and the reaction was stirred for 1 h. $HgCl_2$ (1.5 g, 6 mmol, 1.3 equiv) was added to the reaction which was left to stir for 16 h at rt. The mixture was diluted with DCM (50 mL), filtered over Celite® and the filtrate was washed with satd aq $NaHCO_3$ (2 x 50 mL), water (3 x 50 mL), and the water phases were reextracted with DCM (3 x 20 mL). The combined organic layers were dried over Na_2SO_4 and concentrated under reduced pressure. The pure hexyl glycoside **5β** was precipitated in cold 1:1 Et_2O:toluene and filtered off as a white solid (0.52 g, 56%). Chromatography of the mother liquor (EtOAc-hexanes, 6:4) gave additional pure hexyl glycoside **5β** (0.42 g, 45%). Analytical data for glycoside **5β** is in agreement with reported data [2]. ^1H NMR ($CDCl_3$, 400 MHz) δ_H 5.46 (d, 1H, *J* = 8.7 Hz, NH), 5.29 (dd, 1H, *J* = 9.4, 10.5 Hz, H-3), 5.04 (t, 1H, *J* = 9.6 Hz, H-4), 4.66 (d, 1H, *J* = 8.3 Hz, H-1), 4.23 (dd, 1H, *J* = 4.7, 12.2 Hz, H-6a), 4.10 (dd, 1H, *J* = 2.4, 12.2 Hz, H-6b), 3.96-3.75 (m, 2H, H-2, OCH*H*CH$_2$), 3.69-3.65 (m, 1H, H-5), 3.48-3.41 (m, 1H, OC*H*HCH$_2$), 2.10, 2.02, 2.01, 1.92 (4 s, 12H, 4 × COC*H*$_3$), 1.55 (m, 2H, OCH$_2$C*H*$_2$), 1.31-1.20 (m, 6H, CH$_2$(C*H*$_2$)$_3$CH$_3$), 0.85 (m, 3H, (CH$_2$)$_5$CH$_3$). ^{13}C NMR ($CDCl_3$, 100 MHz) δ_C 170.9, 170.7, 170.1, 169.4 (C=O), 100.7 (C-1), 72.3 (C-4), 71.7 (C-5), 70.0 (OCH$_2$CH$_2$), 68.7 (C-3) 62.2 (C-6), 54.9 (C-2), 31.5 (OCH$_2$CH$_2$), 29.4, 25.5, (CH$_2$(CH$_2$)$_2$CH$_2$), 23.3 (COCH$_3$), 22.6 (CH$_2$CH$_3$), 20.7, 20.7, 20.6 (COCH$_3$), 14.0 (CH$_2$CH$_3$).

1.3. Methyl 2-acetamido-3,4,6-tri-O-acetyl-2-deoxy-β-D-glucopyranoside (6β)

Known 2-acetamido-3,4,6-tri-*O*-acetyl-2-deoxy-β-D-glucopyranosyl chloride [1] (5.1 g, 14 mmol) was dissolved in dry DCM (100 mL) under N_2. Molecular sieves (4 Å, 5 g) were added along with dry MeOH (5 mL, 124 mmol, 9 eq) and the reaction was stirred for 1 h. $HgCl_2$ (3.82 g, 14 mmol, 1 equiv) was added to the reaction which was left to stir for 16 h at rt. The mixture was diluted with DCM (150 mL), filtered over Celite® and the filtrate was washed with satd aq $NaHCO_3$ (2 x 100 mL), water (3 x 100 mL), and the water phases were reextracted with DCM (3 x 50 mL). The combined organic layers were dried over Na_2SO_4 and concentrated under reduced pressure. The methyl glycoside **6β** was precipitated in cold 1:1 Et_2O:toluene and filtered off as an off white solid (1.9 g, 38%). Chromatography of the mother liquor (EtOAc-hexanes, 8:2) gave additional pure methyl glycoside **6β** (2.5 g, 50%). %). Analytical data for glycoside **6β** is in

agreement with reported data [3]. ^{1}H NMR ($CDCl_3$, 400 MHz) δ_H 5.45 (d, 1H, *J* = 8.9 Hz, NH), 5.25 (dd, 1H, *J* = 9.3, 10.4 Hz, H-3), 5.06 (t, 1H, *J* = 9.8 Hz, H-4), 4.56 (d, 1H, *J* = 8.3 Hz, H-1), 4.26 (dd, 1H, *J* = 4.7, 12.3 Hz, H-6a), 4.13 (dd, 1H, *J* = 2.5, 12.3 Hz, H-6b), 3.84 (m, 1H, H-2), 3.68 (m, 1H, H-5), 3.48 (s, 3H, OCH_3), 2.08, 2.01, 2.00, 1.94 (4 s, 12H, 4 × COCH_3). 13C NMR ($CDCl_3$, 100 MHz): δ_C 171.0, 170.7, 170.2, 169.4 (C=O), 101.6 (C-1), 72.4 (C-4), 71.8 (C-5), 68.5 (C-3), 62.1 (C-6), 56.8 (CH_3), 54.6 (C-2), 23.4, 20.7, 20.7, 20.6 ($COCH_3$).

2. X-ray Crystallography of dimethylmethyleniminium bromide (III):

2.1. Experimental

Crystals were mounted on a cryoloop with paratone oil and examined on a Bruker Dual Source D8 Venture diffractometer using the IμS 3.0 Mo source (λ = 0.71073 Å) with a Photon III area detector in the McMaster Analytical X-ray (MAX) Diffraction Facility. An Oxford Cryostream cooler was used to maintain cryogenic temperatures of 100 K for this study. Data were collected using the APEX v5 software [4], integrated using SAINT [5] and corrected for absorption using a multi-scan approach (SADABS) [6]. Final cell constants were determined from full least squares refinement of all observed reflections. This structure was solved using intrinsic phasing (SHELXT) [7] and refined with full least squares refinement on F^2 using SHELXL [8] in Olex2-1.5 software [9].
Data Availability: Crystallographic data for dimethylmethyleniminium bromide salt III has been deposited at the CCDC with the deposition number 2425977.

2.2. Crystal Structure of dimethylmethyleniminium bromide (III).

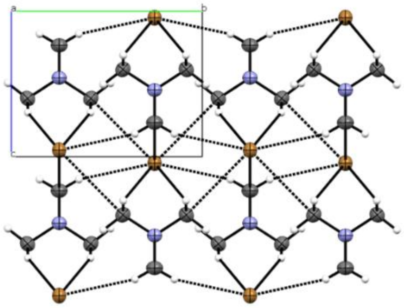

A. Crystal packing of **III** viewed along the crystallographic a-axis. Thermal ellipsoids are drawn at the 50% probability level and there is no labelling.

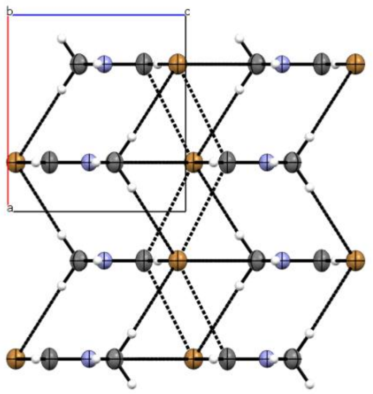

B. Crystal packing of **III** viewed along the crystallographic b-axis. Thermal ellipsoids are drawn at the 50% probability level and there is no labelling.

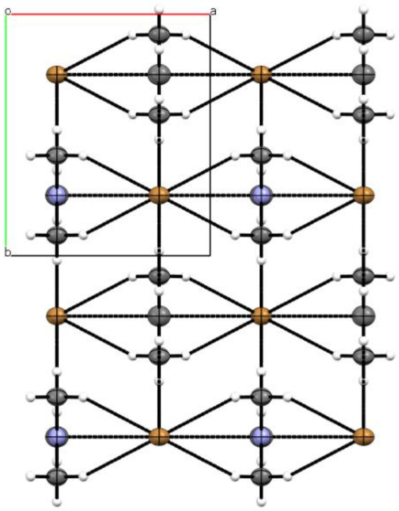

C. Crystal packing of **III** viewed along the crystallographic ac-axis. Thermal ellipsoids are drawn at the 50% probability level and there is no labelling.

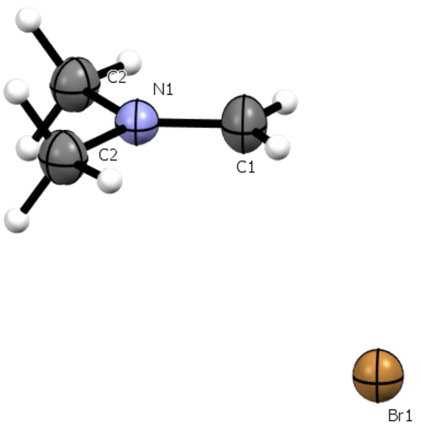

D. Unit cell of **III** showing structure planarity with H atoms shown and thermal ellipsoids are drawn at the 50% probability level with non H atoms labelled.

LW_NC_39B_Pmmn_a

Table 1 Crystal data and structure refinement for LW_NC_39B_Pmmn_a.

Identification code	LW_NC_39B_Pmmn_a
Empirical formula	C_3H_8BrN
Formula weight	138.01
Temperature/K	100.00
Crystal system	orthorhombic
Space group	Pmmn
a/Å	6.2606(5)
b/Å	7.4223(5)
c/Å	5.6570(5)
α/°	90
β/°	90
γ/°	90
Volume/Å^3	262.87(4)
Z	2
ρ_{calc}g/cm^3	1.744
μ/mm^{-1}	7.653
F(000)	136.0
Crystal size/mm^3	0.176 × 0.141 × 0.071
Radiation	MoKα (λ = 0.71073)
2Θ range for data collection/°	7.204 to 52.73
Index ranges	$-7 \le h \le 7$, $-9 \le k \le 9$, $-7 \le l \le 7$
Reflections collected	6182
Independent reflections	316 [R_{int} = 0.0596, R_{sigma} = 0.0297]
Data/restraints/parameters	316/0/29
Goodness-of-fit on F^2	1.130
Final R indexes [I>=2σ (I)]	R_1 = 0.0169, wR_2 = 0.0338
Final R indexes [all data]	R_1 = 0.0174, wR_2 = 0.0340
Largest diff. peak/hole / e Å^{-3}	0.20/-0.52

Table 2 Fractional Atomic Coordinates ($\times10^4$) and Equivalent Isotropic Displacement Parameters ($Å^2\times10^3$) for LW_NC_39B_Pmmn_a. U_{eq} is defined as 1/3 of the trace of the orthogonalised U_{IJ} tensor.

Atom	*x*	*y*	*z*	U(eq)
Br1	7500	7500	10446.1(4)	36.64(14)
N1	2500	7500	5419(4)	30.5(5)
C2	2500	5841(3)	4002(4)	38.1(4)
C1	2500	7500	7656(5)	40.9(6)

Table 3 Anisotropic Displacement Parameters ($Å^2 \times 10^3$) for LW_NC_39B_Pmmn_a. The Anisotropic displacement factor exponent takes the form: $-2\pi^2[h^2a^{*2}U_{11}+2hka^*b^*U_{12}+\ldots]$.

Atom	U_{11}	U_{22}	U_{33}	U_{23}	U_{13}	U_{12}
Br1	43.76(19)	29.71(17)	36.4(2)	0	0	0
N1	30.5(10)	34.2(12)	26.9(12)	0	0	0
C2	44.7(10)	34.2(10)	35.3(9)	-4.0(8)	0	0
C1	51.2(14)	42.2(14)	29.3(14)	0	0	0

Table 4 Bond Lengths for LW_NC_39B_Pmmn_a.

Atom	Atom	Length/Å	Atom	Atom	Length/Å
N1	C2	1.470(2)	N1	C1	1.265(4)
N1	C2[1]	1.470(2)			

[1]1/2-X,3/2-Y,+Z

Table 5 Bond Angles for LW_NC_39B_Pmmn_a.

Atom	Atom	Atom	Angle/°	Atom	Atom	Atom	Angle/°
C2[1]	N1	C2	113.9(2)	C1	N1	C2	123.07(11)
C1	N1	C2[1]	123.07(11)				

[1]1/2-X,3/2-Y,+Z

Table 6 Hydrogen Atom Coordinates (Å×10^4) and Isotropic Displacement Parameters (Å^2×10^3) for LW_NC_39B_Pmmn_a.

Atom	*x*	*y*	*z*	U(eq)
H1	2500	6470(30)	8430(60)	47(6)
H2A	2500	4810(50)	5050(50)	45(7)
H2B	1210(20)	5860(20)	3030(30)	44(4)

LW_NC_39B_Pmmn_a

Table 1 Crystal data and structure refinement for LW_NC_39B_Pmmn_a.

Identification code	LW_NC_39B_Pmmn_a
Empirical formula	C_3H_8BrN
Formula weight	138.01
Temperature/K	100.00
Crystal system	orthorhombic
Space group	Pmmn
a/Å	6.2606(5)
b/Å	7.4223(5)
c/Å	5.6570(5)
α/°	90
β/°	90
γ/°	90
Volume/Å^3	262.87(4)
Z	2
ρ_{calc}g/cm^3	1.744
μ/mm^{-1}	7.653
F(000)	136.0
Crystal size/mm^3	0.176 × 0.141 × 0.071
Radiation	MoKα (λ = 0.71073)
2Θ range for data collection/°	7.204 to 52.73
Index ranges	-7 ≤ h ≤ 7, -9 ≤ k ≤ 9, -7 ≤ l ≤ 7
Reflections collected	6182
Independent reflections	316 [R_{int} = 0.0596, R_{sigma} = 0.0297]
Data/restraints/parameters	316/0/29
Goodness-of-fit on F^2	1.130
Final R indexes [I>=2σ (I)]	R_1 = 0.0169, wR_2 = 0.0338
Final R indexes [all data]	R_1 = 0.0174, wR_2 = 0.0340
Largest diff. peak/hole / e Å^{-3}	0.20/-0.52

3. References

1. Horton, D., 2-Acetamido-3,4,6-tri-O-acetyl-2-deoxy-α-D-glucopyranosyl choride. *Org. Synth.* **1966**, *46,* 1-5.
2. Heskamp, B. M.; Veeneman, G. H.; Vandermarel, G. A.; Vanboeckel, C. A. A.; Vanboom, J. H., Synthesis of a Sle(X) Mimic - a Potential E-Selectin Binding Antagonist. *Recl. Trav. Chim. Pays-Bas* **1995**, *114,* 398-402.
3. Myers, W. H.; Robertson, G. J., The synthesis of amino sugars I. *J. Am. Chem. Soc.* **1943**, *65,* 8-11.
4. Bruker. *APEX 5*; Bruker AXS Inc.: Madison, WI, USA, **2023**.
5. Bruker. *SAINT*; Bruker AXS Inc., Madison, WI, USA, **2020**.
6. Bruker. *SADABS*, Bruker AXS, Madison, WI, USA, **2015**.
7. G. M. Sheldrick, SHELXT-Integrated Space-Group and Crystal-Structure Determination.. *Acta Crystallogr., Sect. A: Found. Adv.* **2015**, *71*, 3–8.
8. G. M. Sheldrick, Crystal structure refinement with SHELXL. *Acta Crystallogr., Sect. C: Struct. Chem.* **2015**, *71*, 3–8.
9. O. V. Dolomanov, L. J. Bourhis, R. J. Gildea, J. K. A. Howard, H. Puschmann, OLEX2: A Complete Structure Solution, Refinement and Analysis Program. *J. Appl. Crystallogr.* **2009**, *42*, 339–341.

Compound **2,** $CDCl_3$, 400 MHz

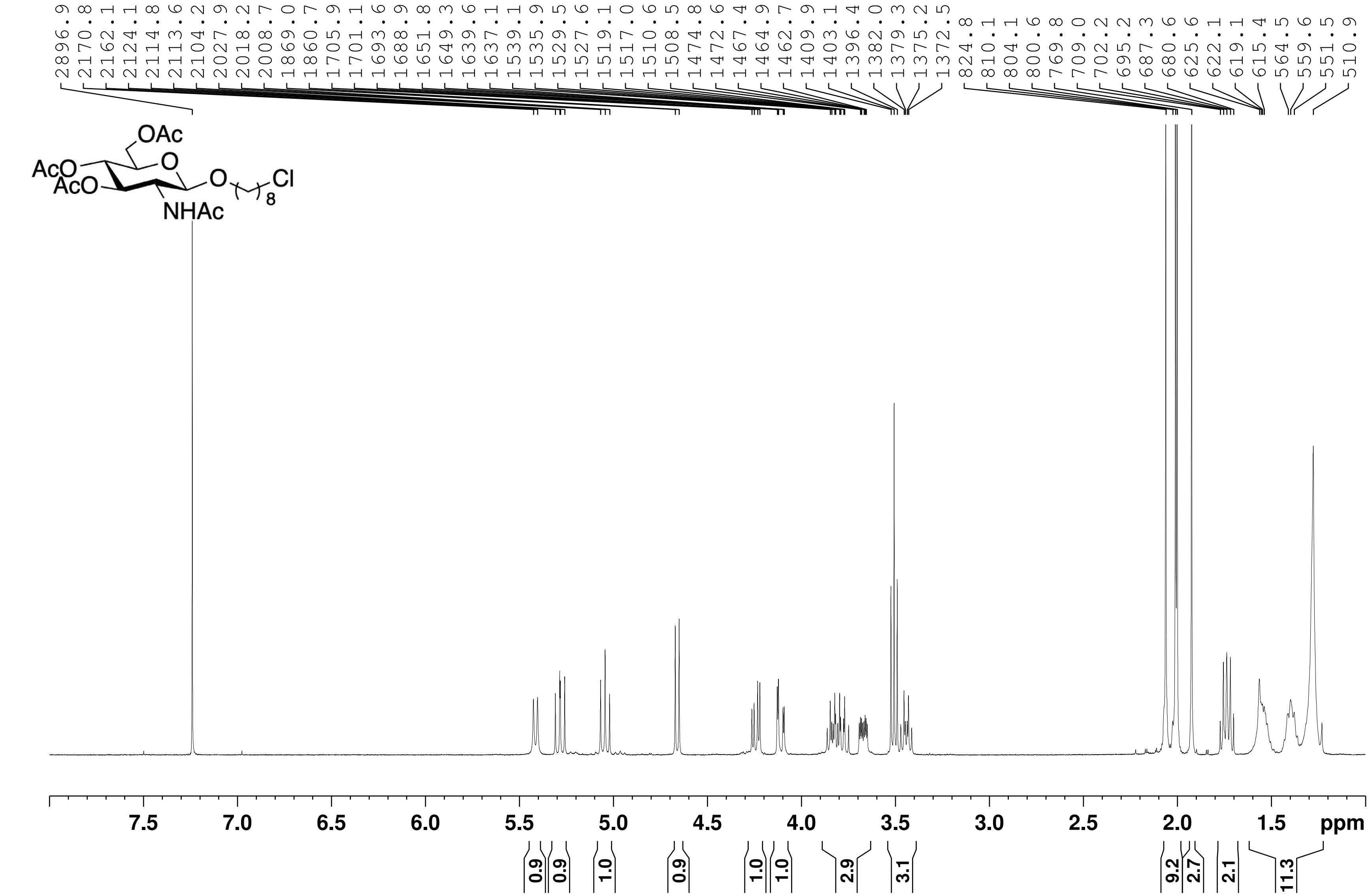

Compound **2,** $CDCl_3$, 400 MHz

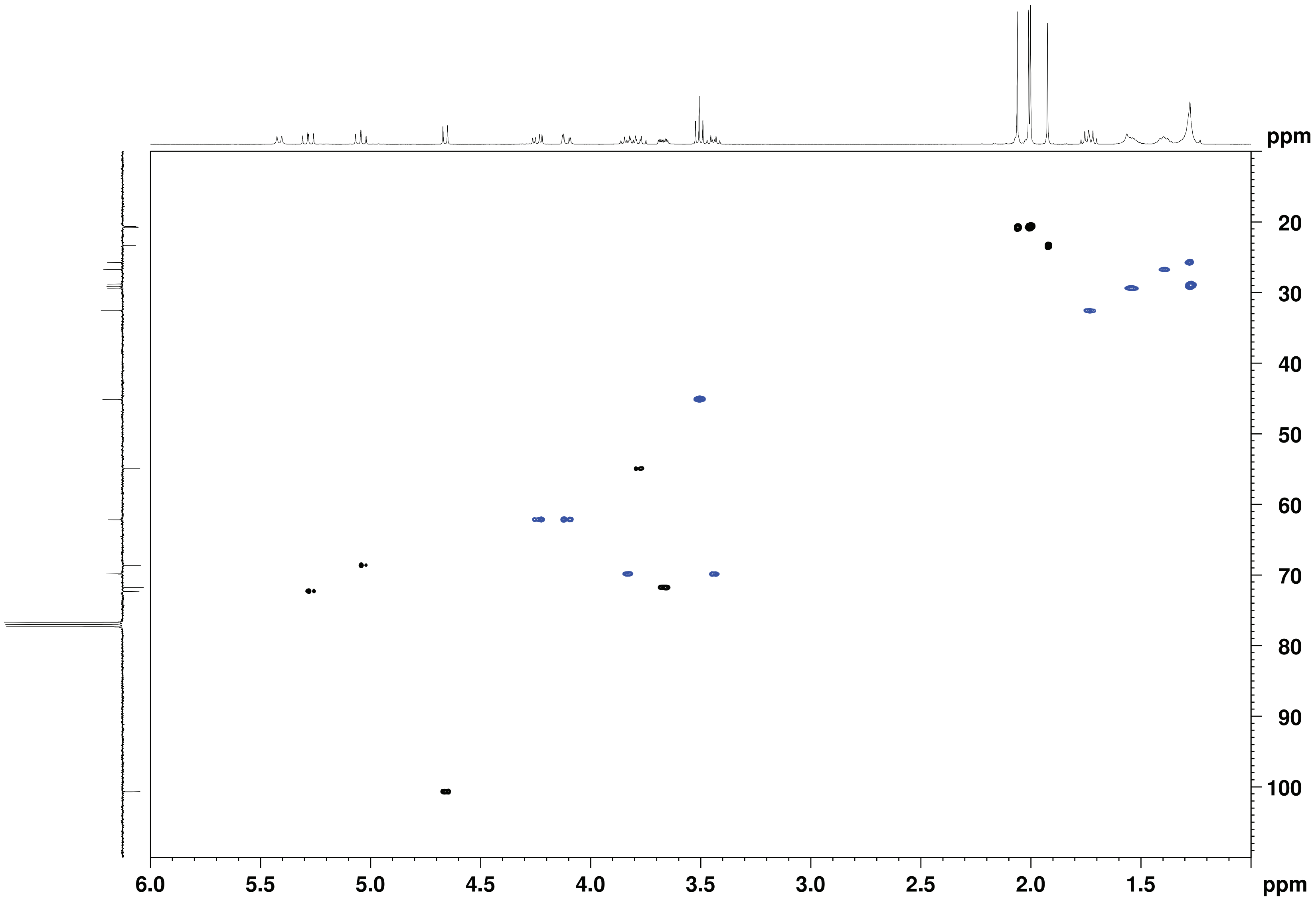

Compound **2,** $CDCl_3$, 100 MHz

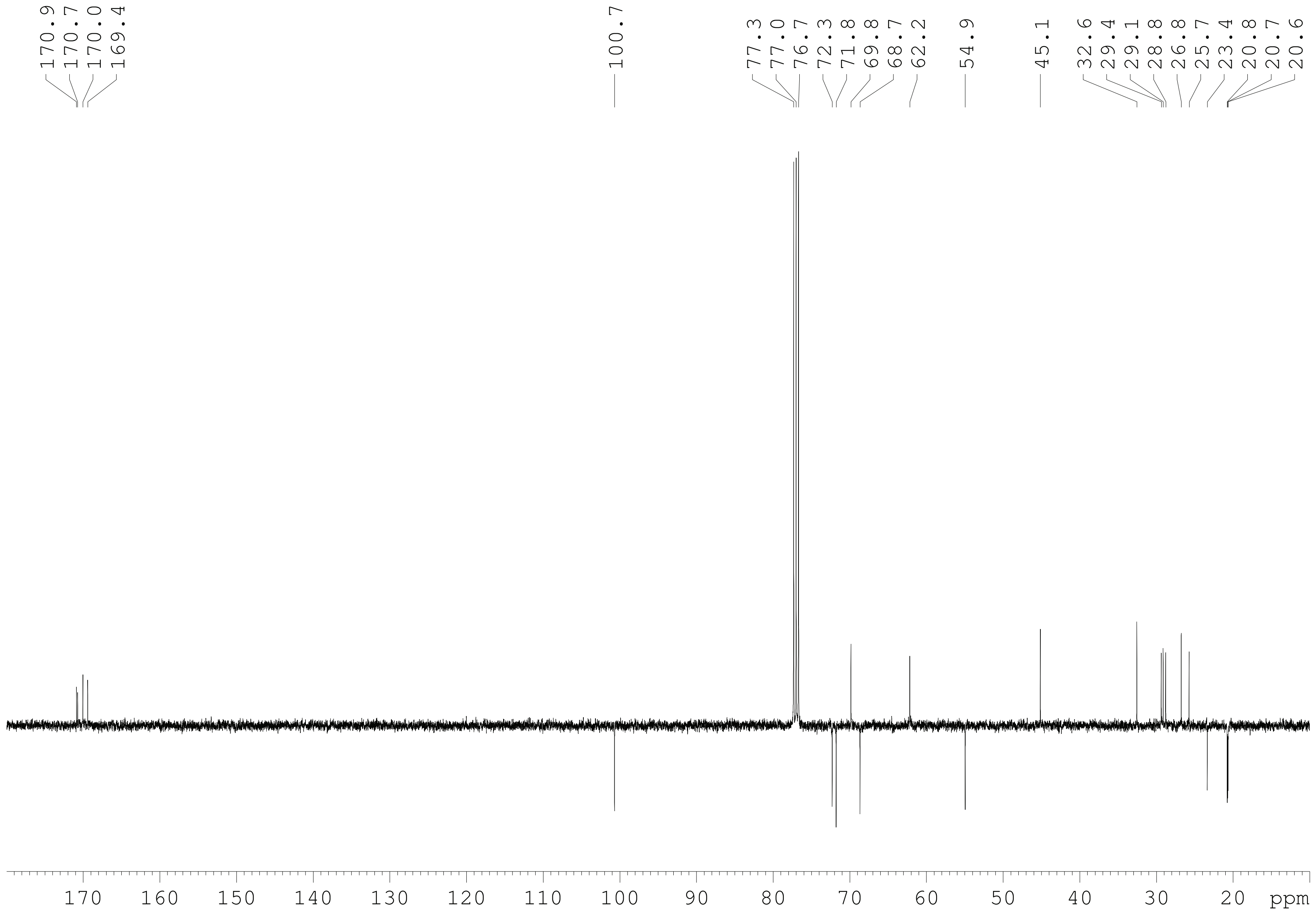

Compound **2,** $CDCl_3$, 400 MHz

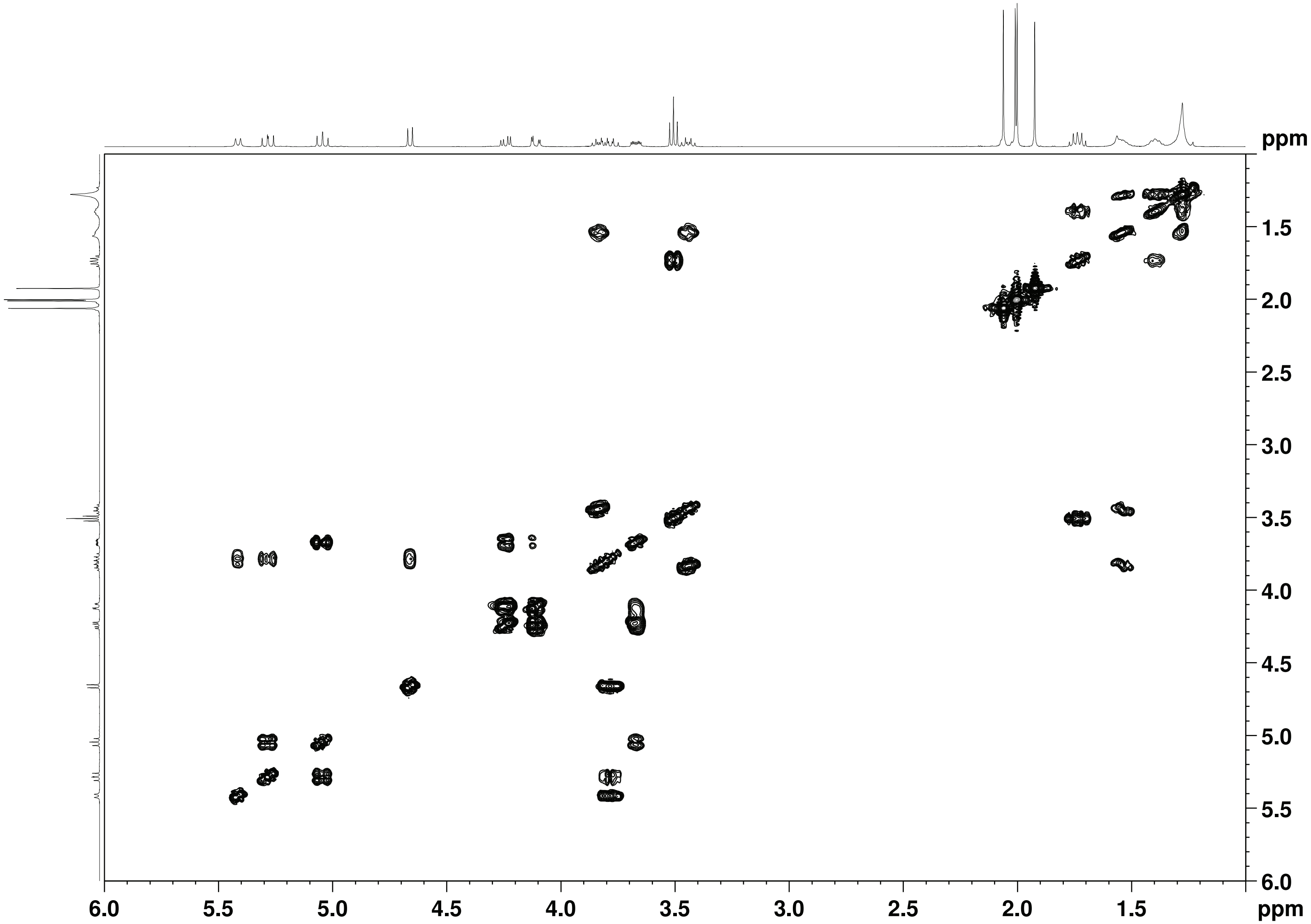

Compound **3α**, $CDCl_3$, 400 MHz

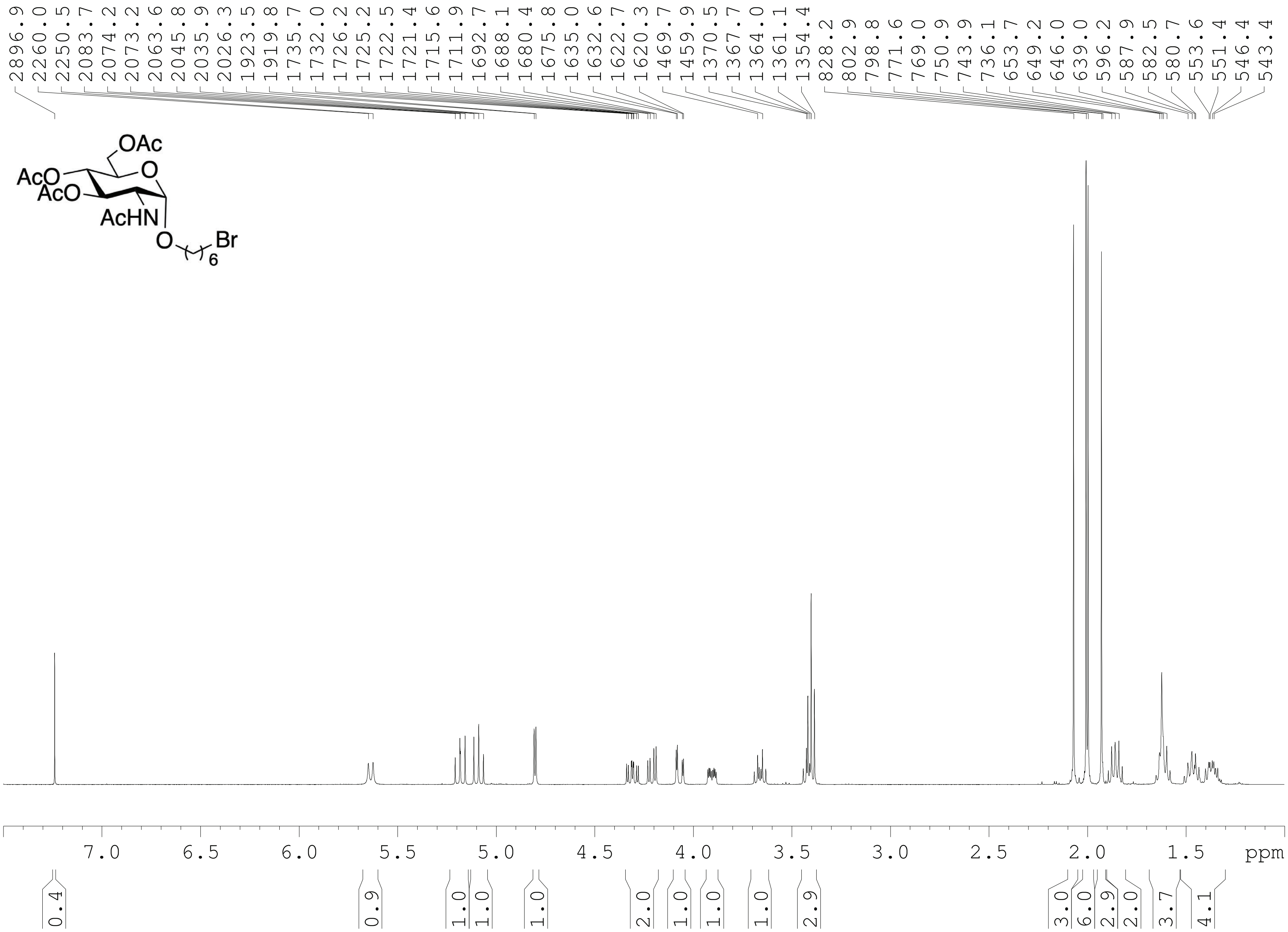

Compound **3α**, $CDCl_3$, 400 MHz

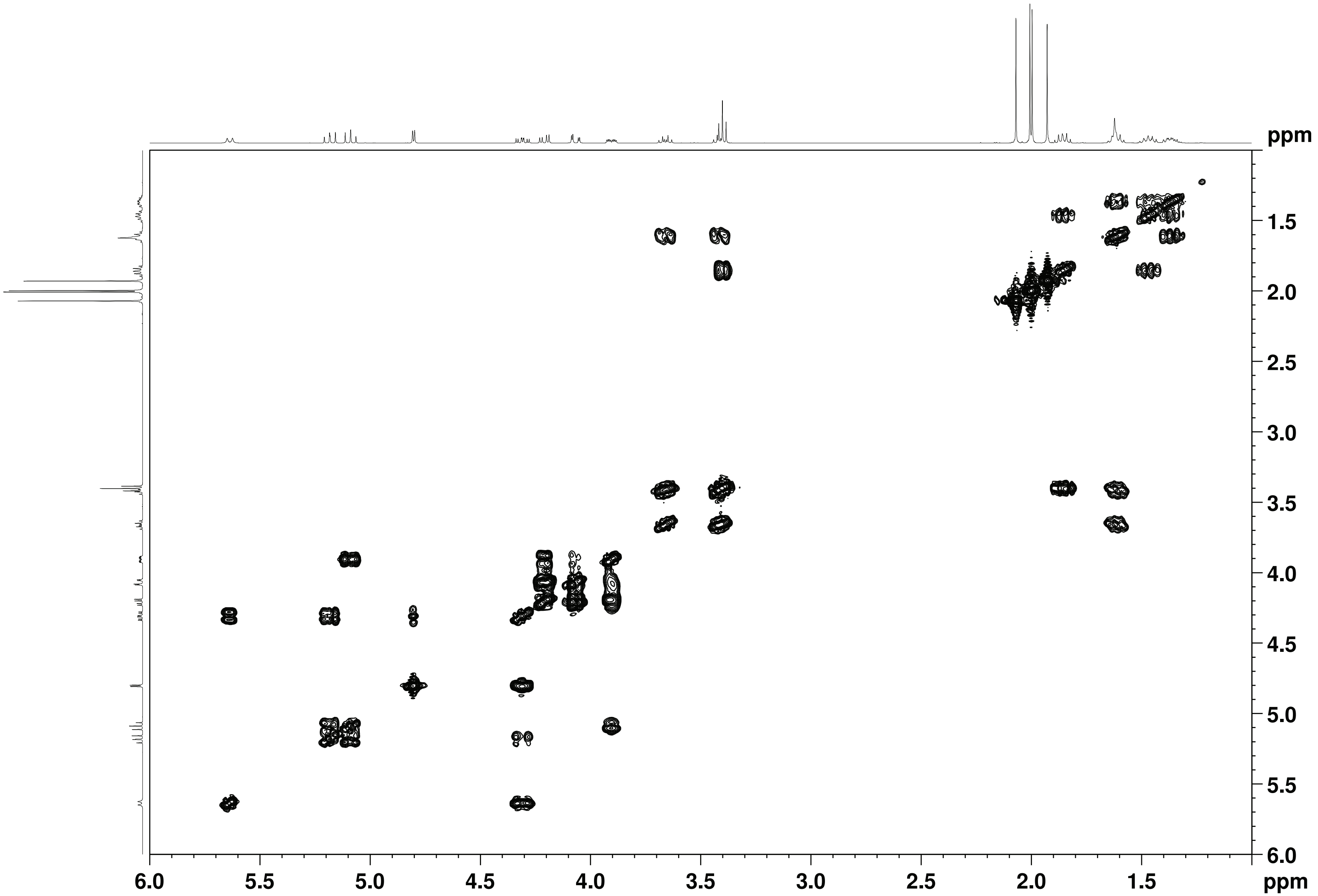

Compound **3α**, $CDCl_3$, 100 MHz

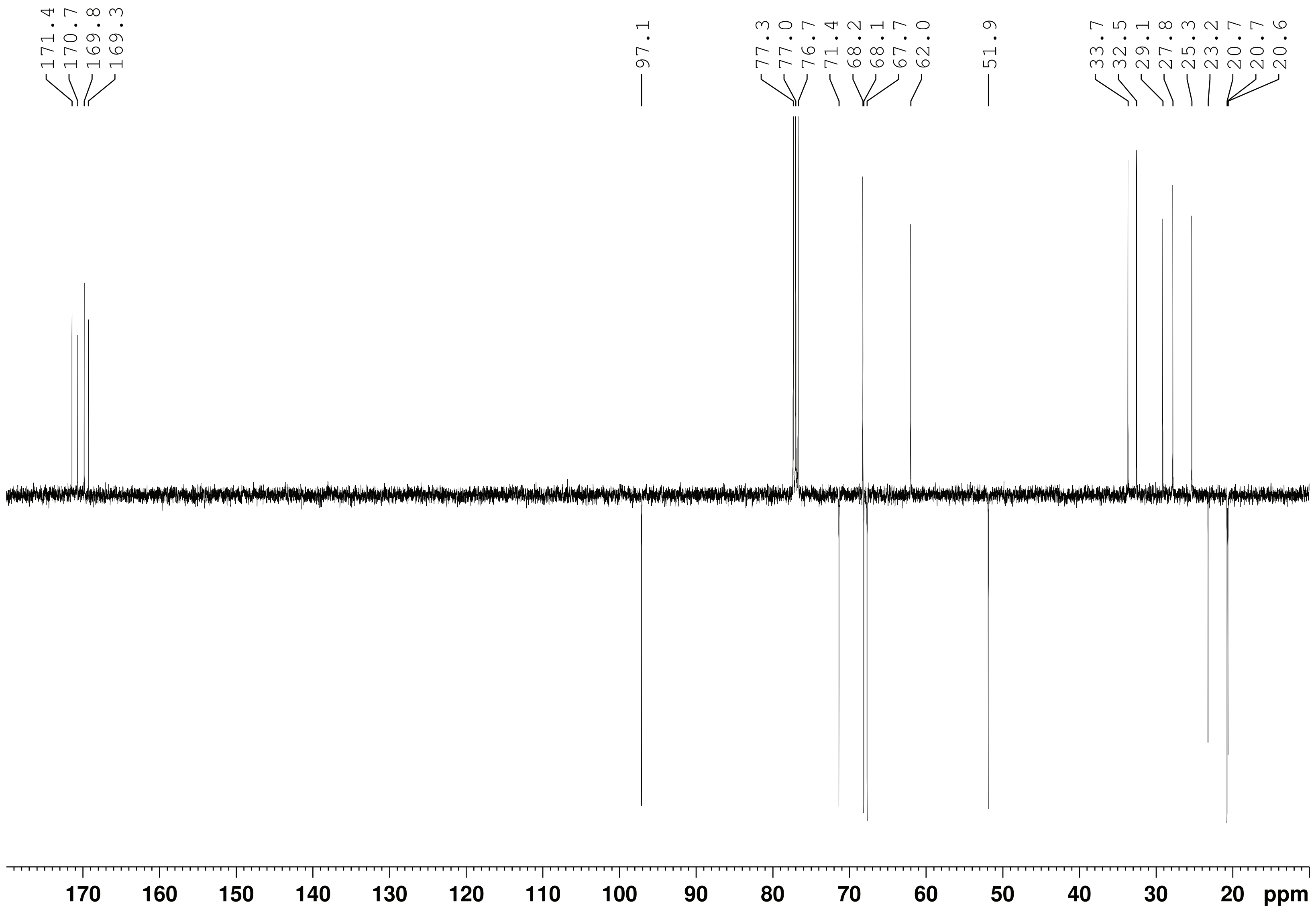

Compound **3α**, $CDCl_3$, 400 MHz

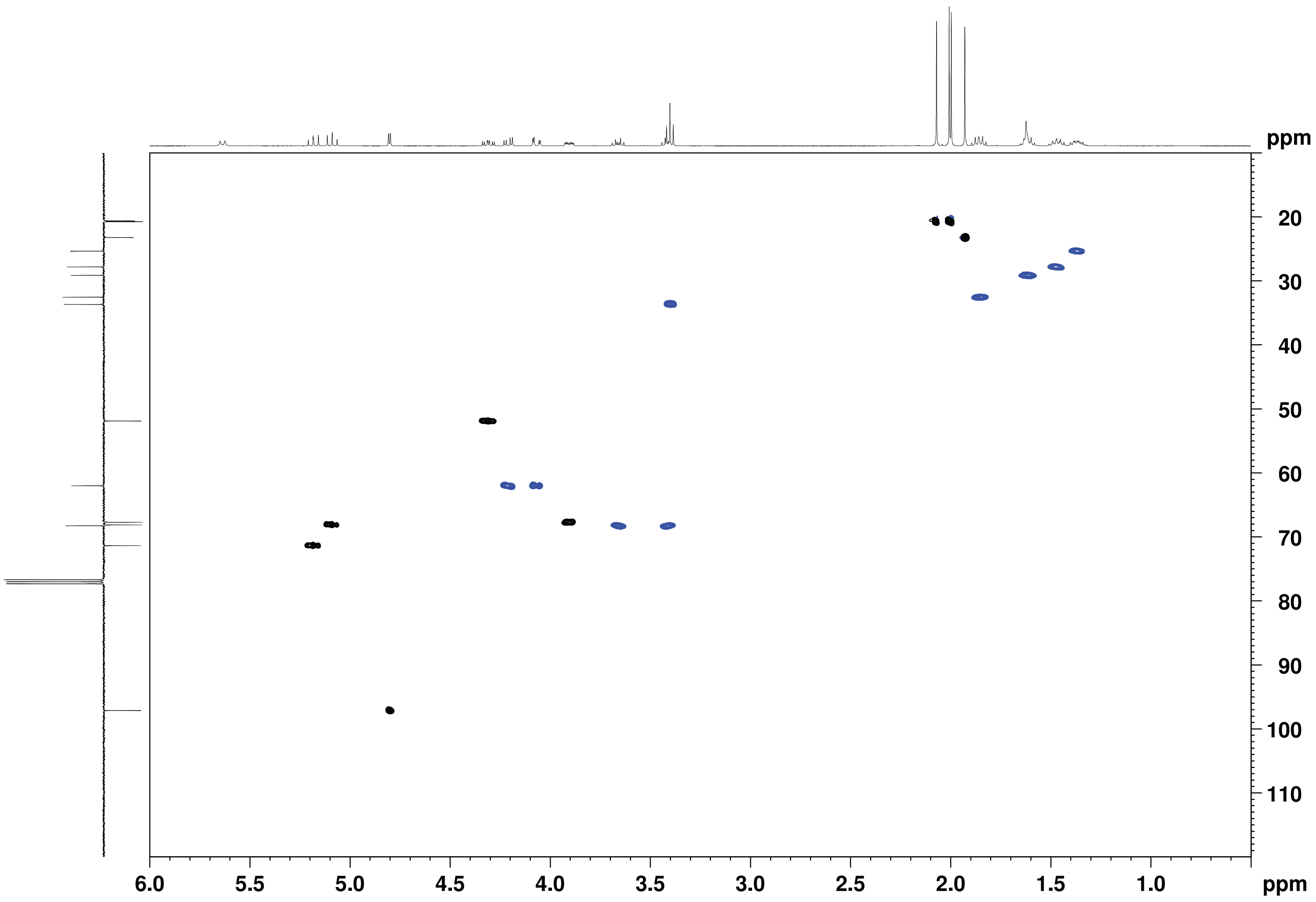

Compound **3β**, $CDCl_3$, 400 MHz

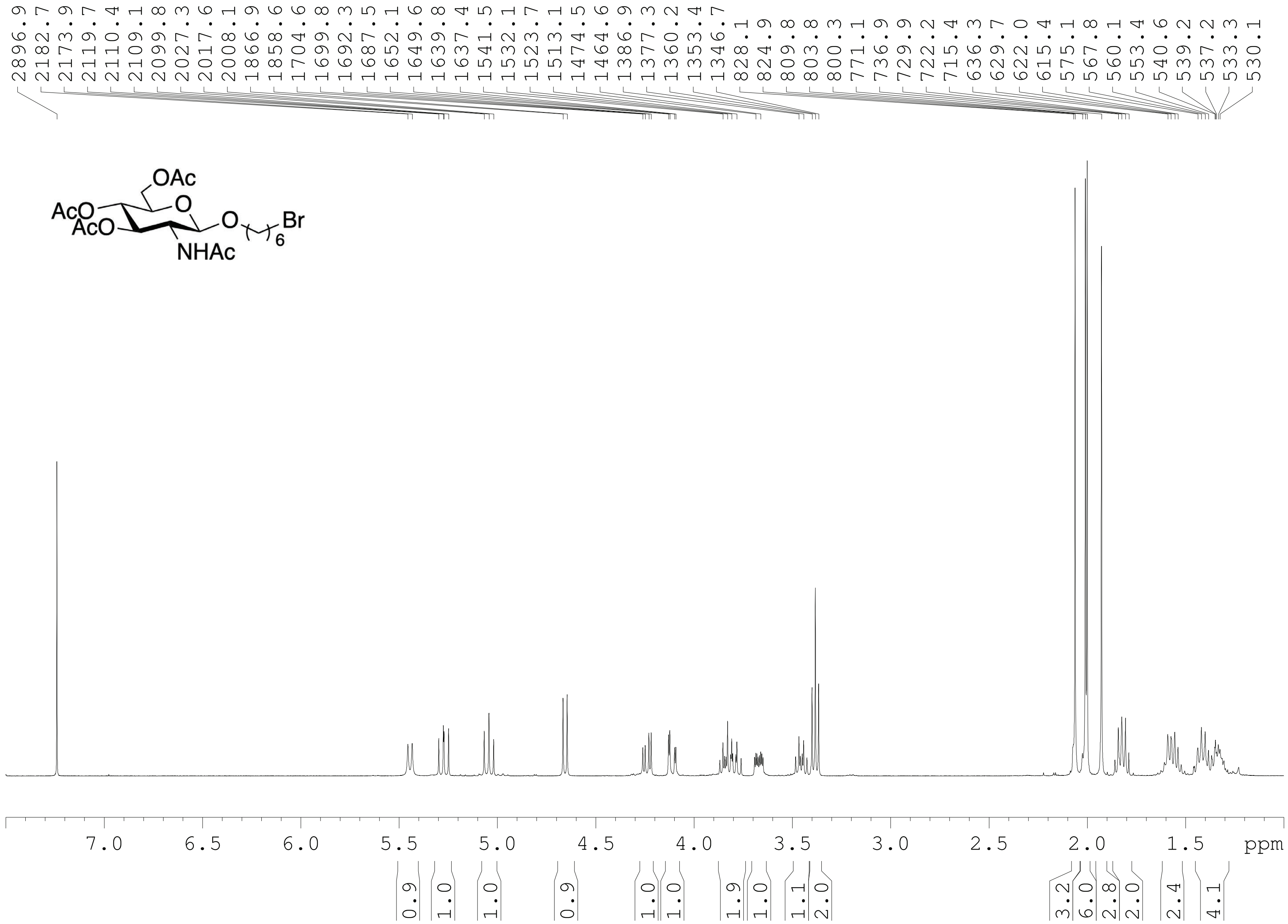

Compound **3β**, $CDCl_3$, 400 MHz

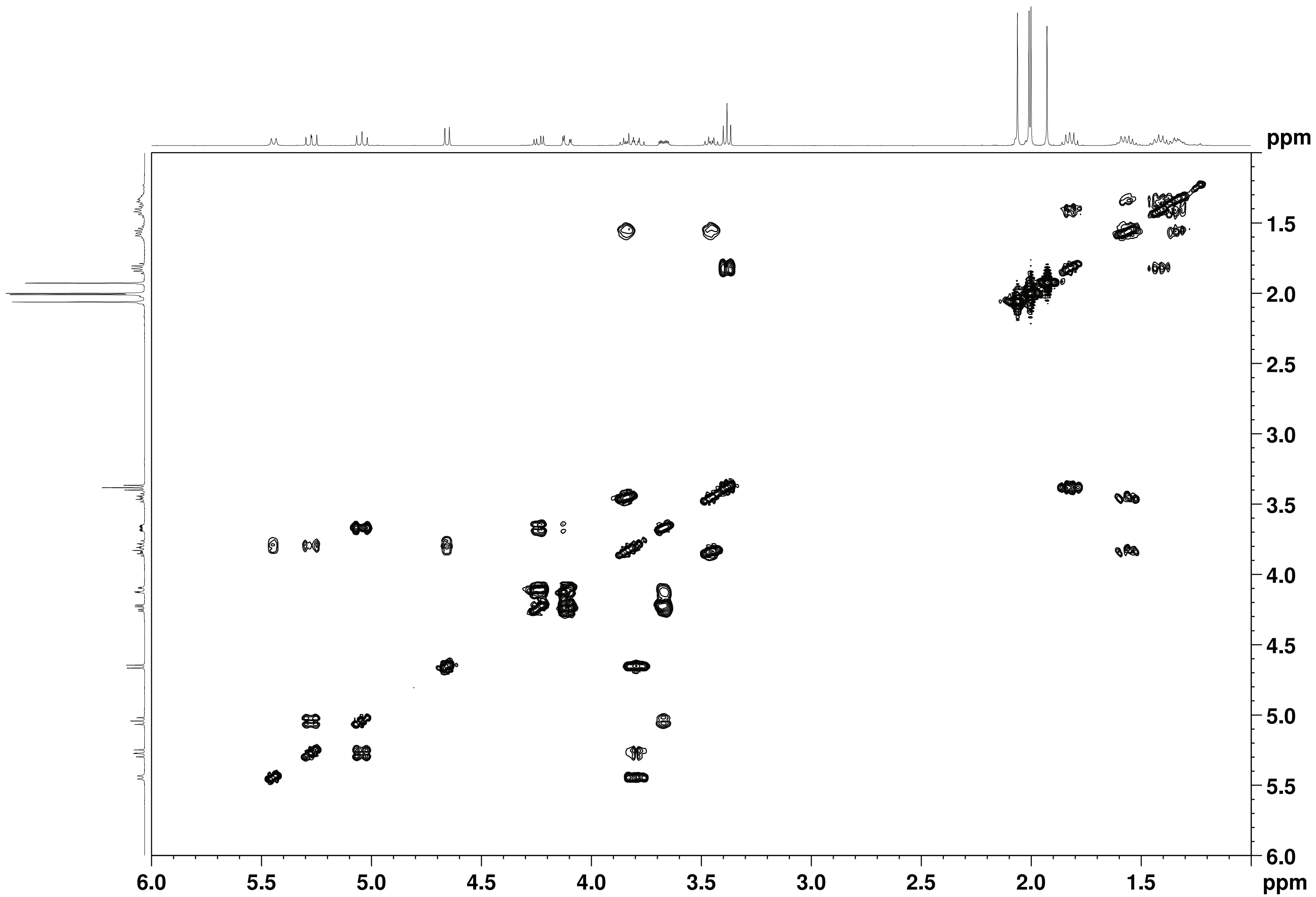

Compound **3β**, $CDCl_3$, 100 MHz

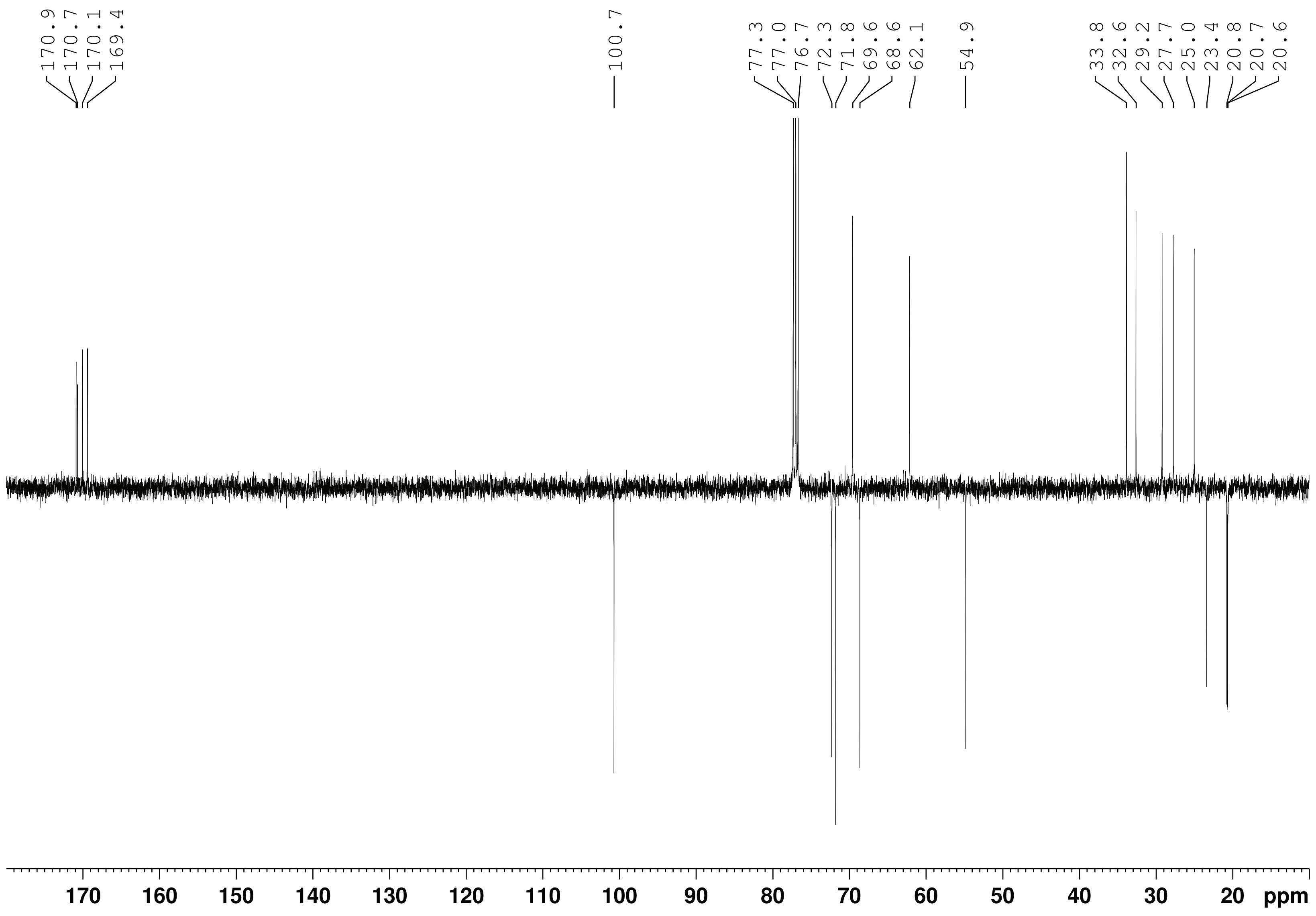

Compound **3β**, $CDCl_3$, 400 MHz

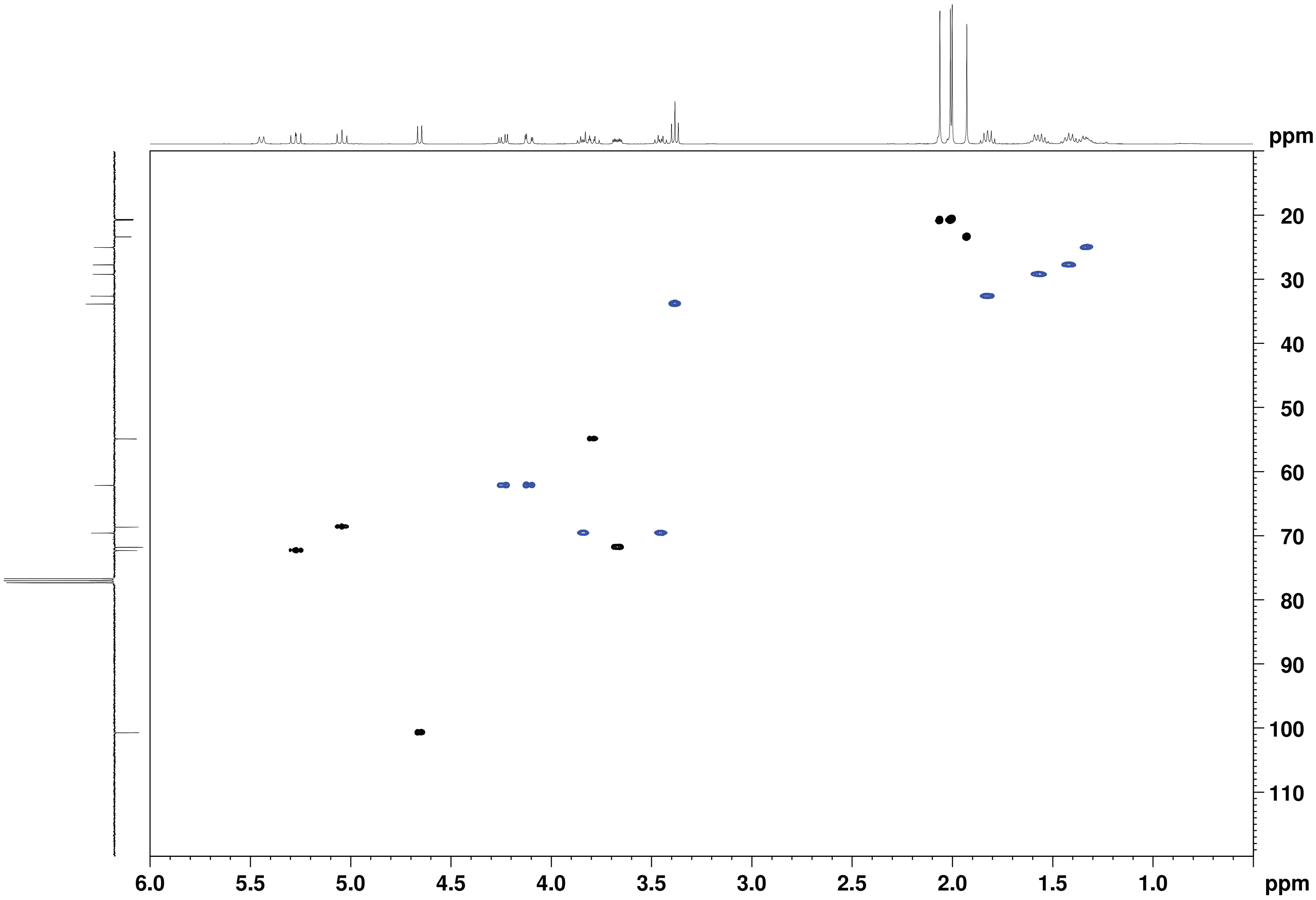

Compound **4α**, $CDCl_3$, 400 MHz

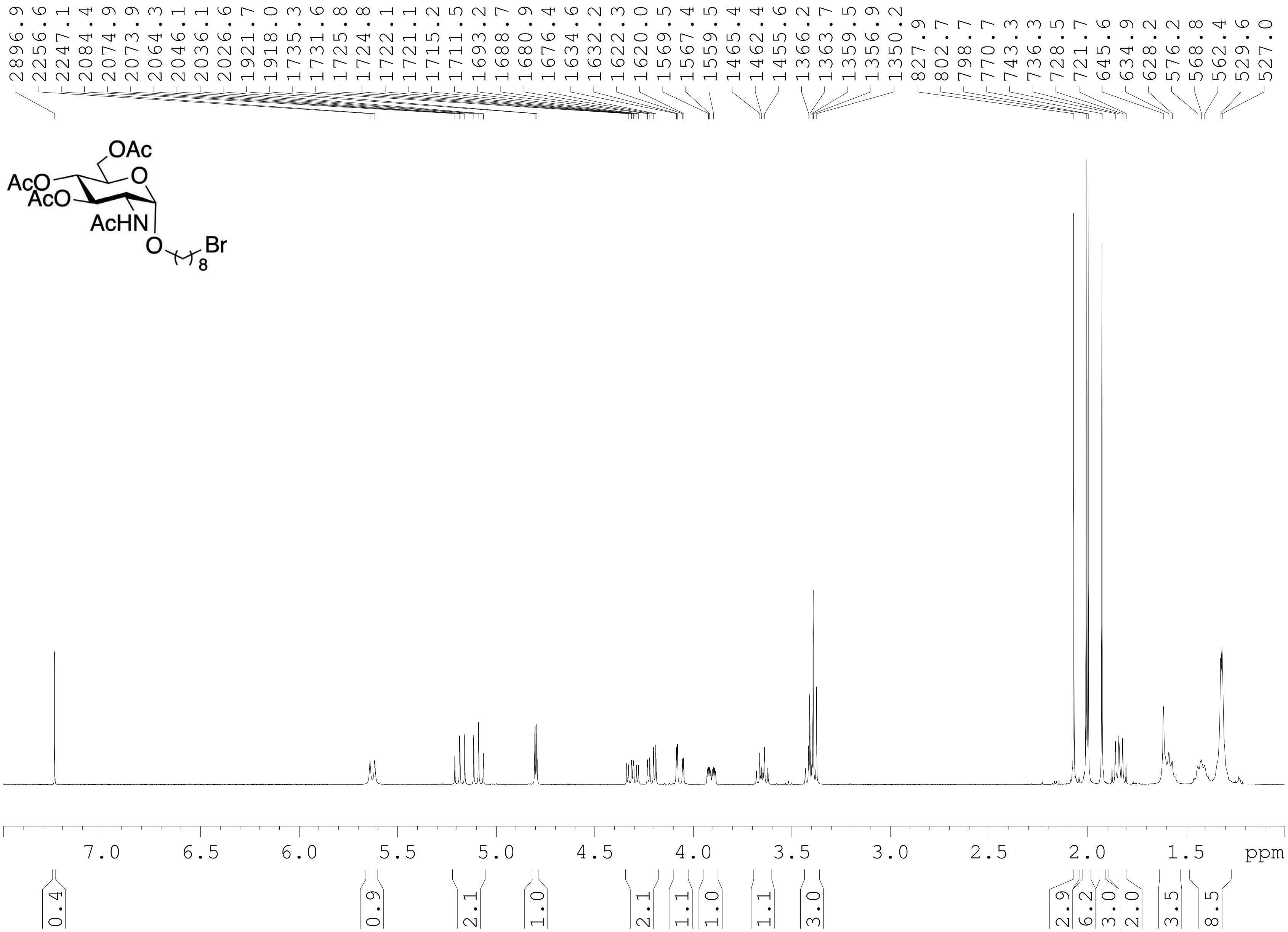

Compound **4α**, $CDCl_3$, 400 MHz

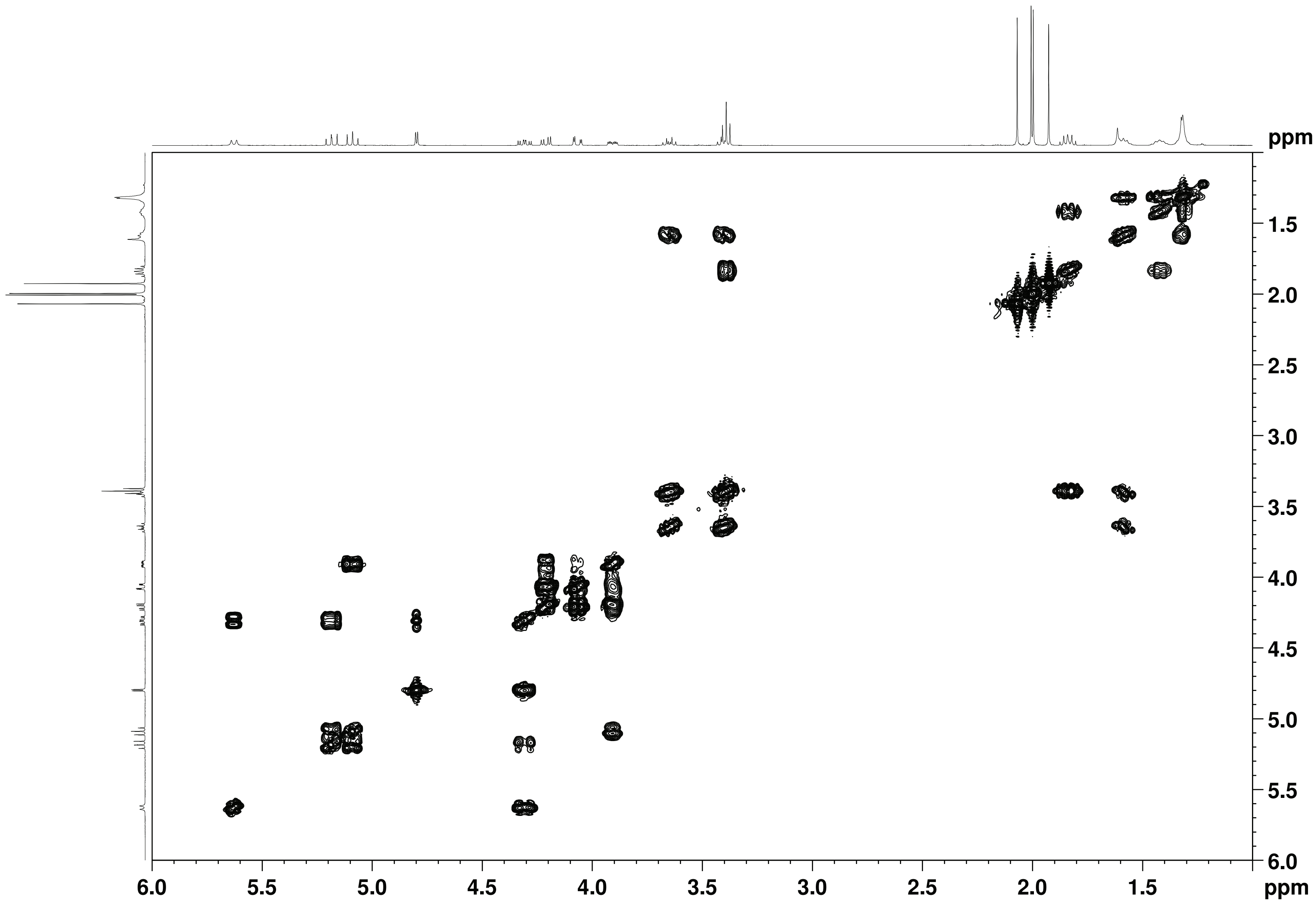

Compound **4α**, $CDCl_3$, 100 MHz

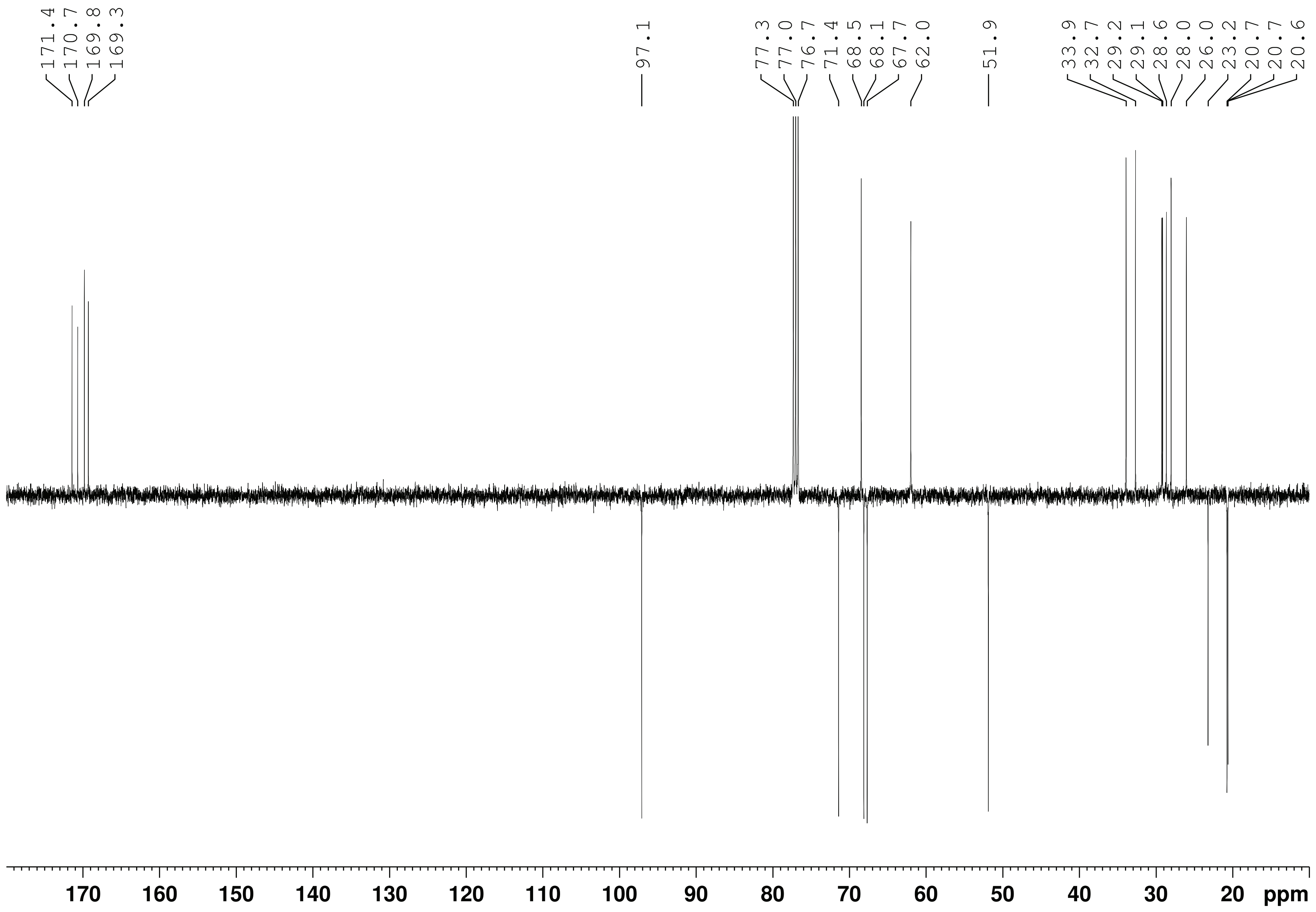

Compound **4α**, $CDCl_3$, 400 MHz

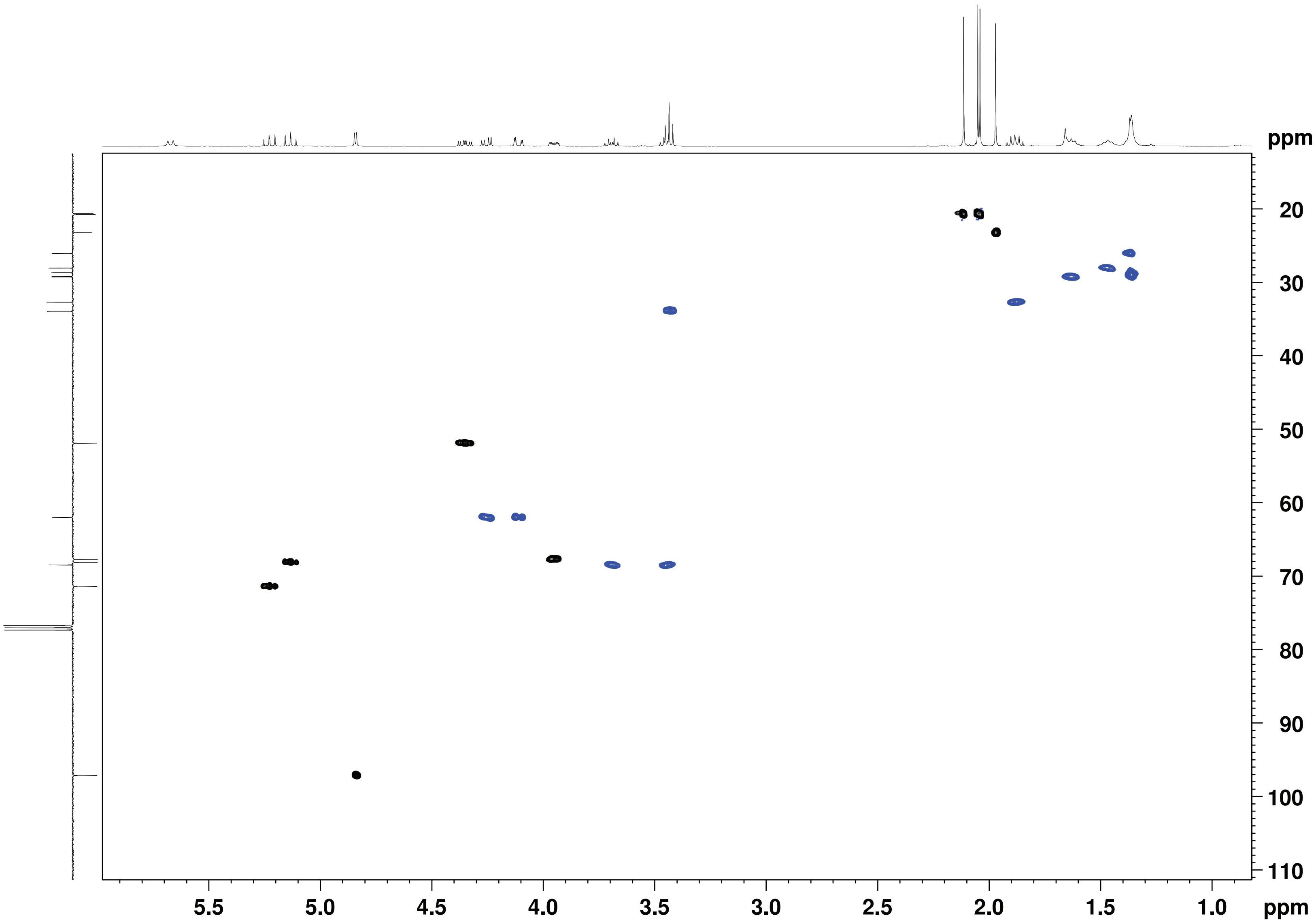

Compound **4β**, $CDCl_3$, 400 MHz

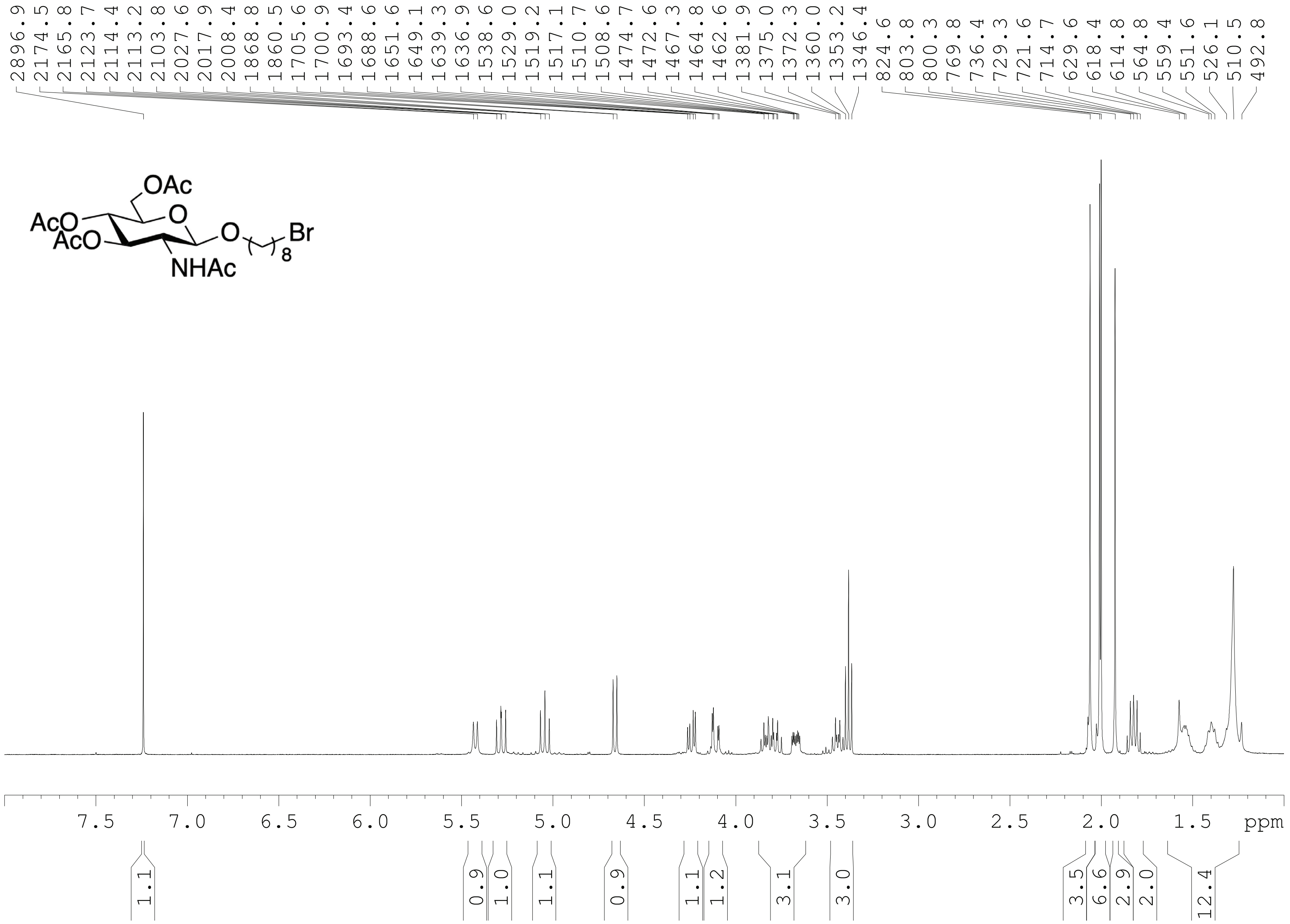

Compound **4β**, $CDCl_3$, 400 MHz

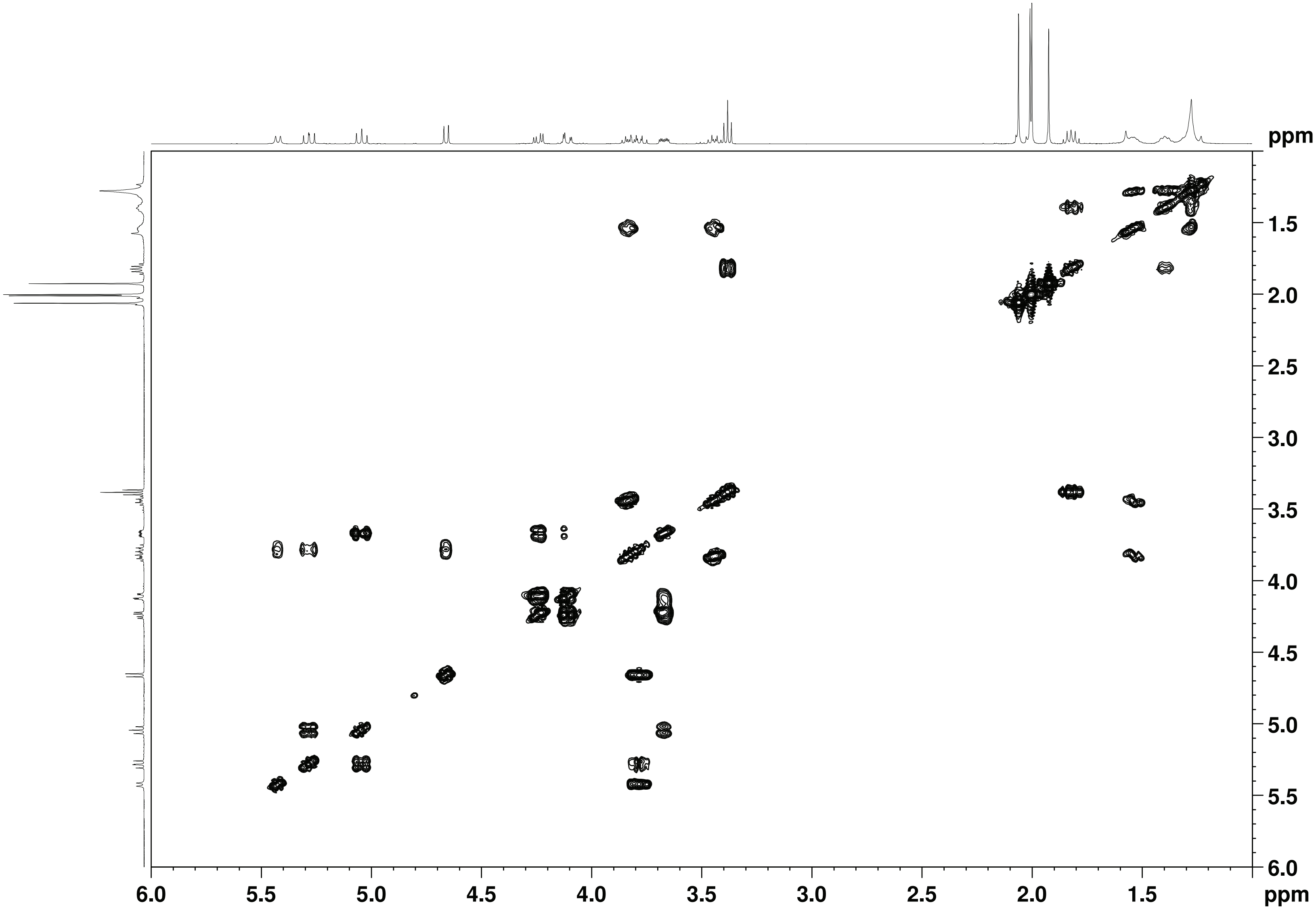

Compound **4β**, $CDCl_3$, 100 MHz

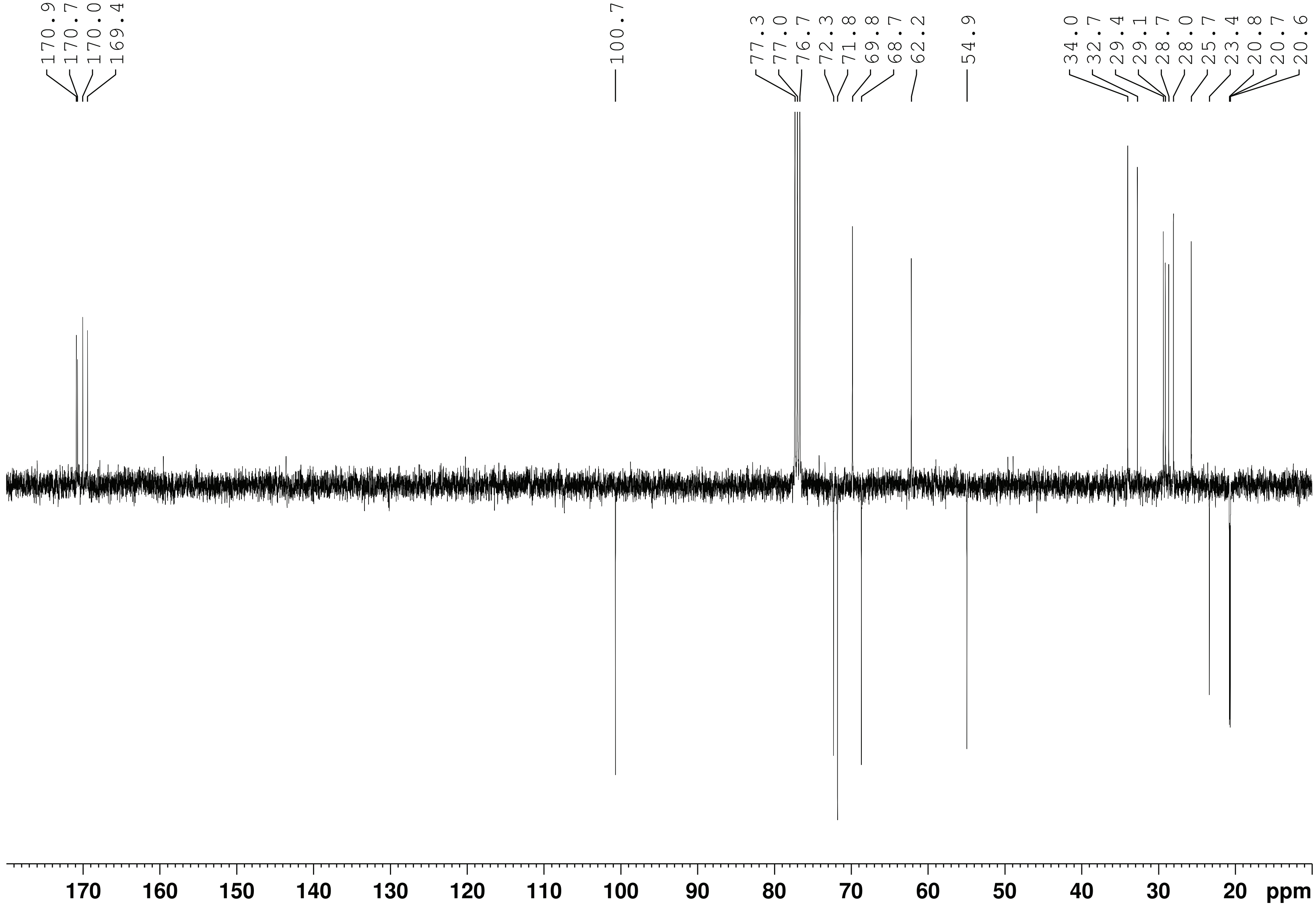

Compound **4β**, $CDCl_3$, 400 MHz

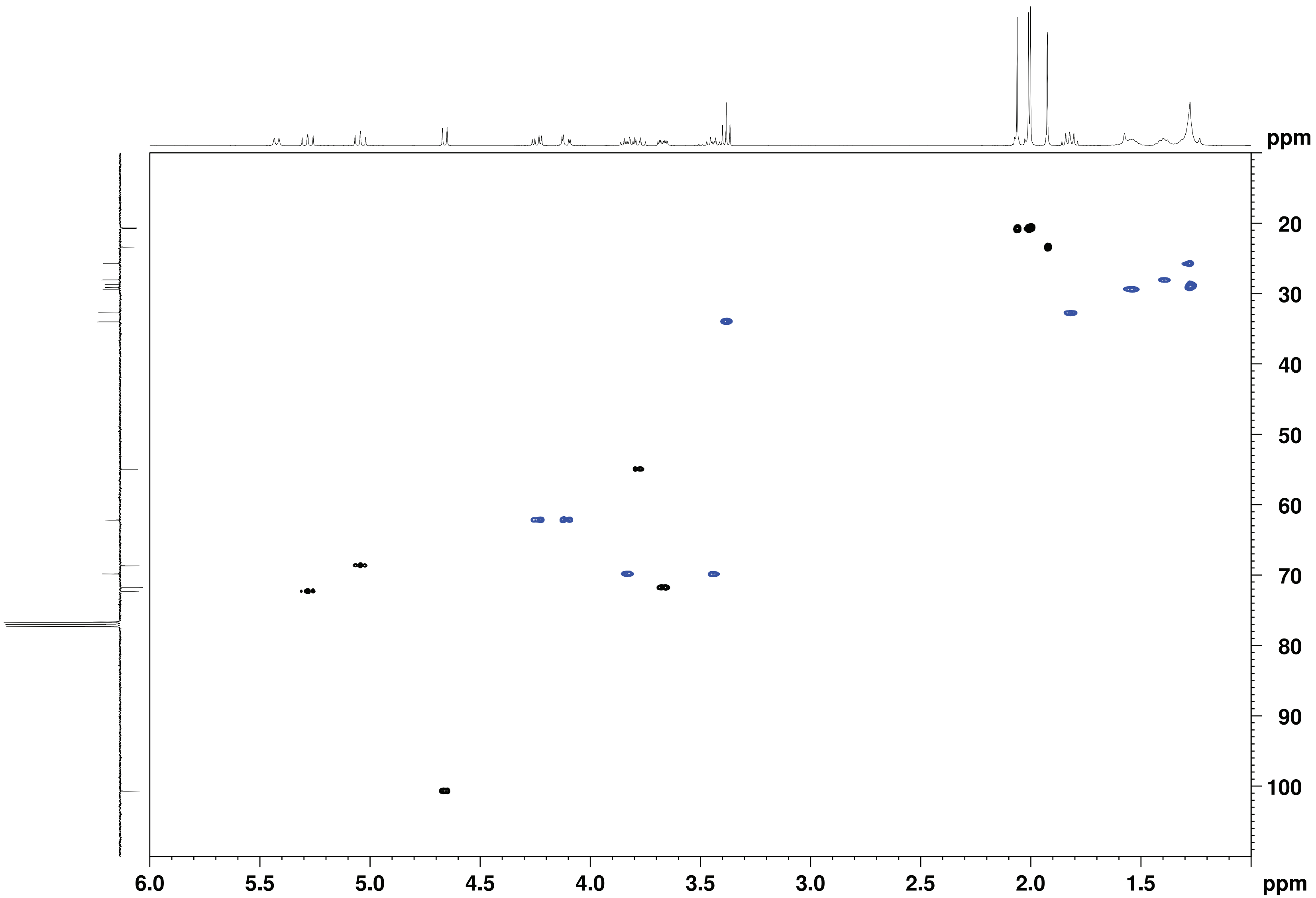

Compound **1**, $CDCl_3$, 400 MHz

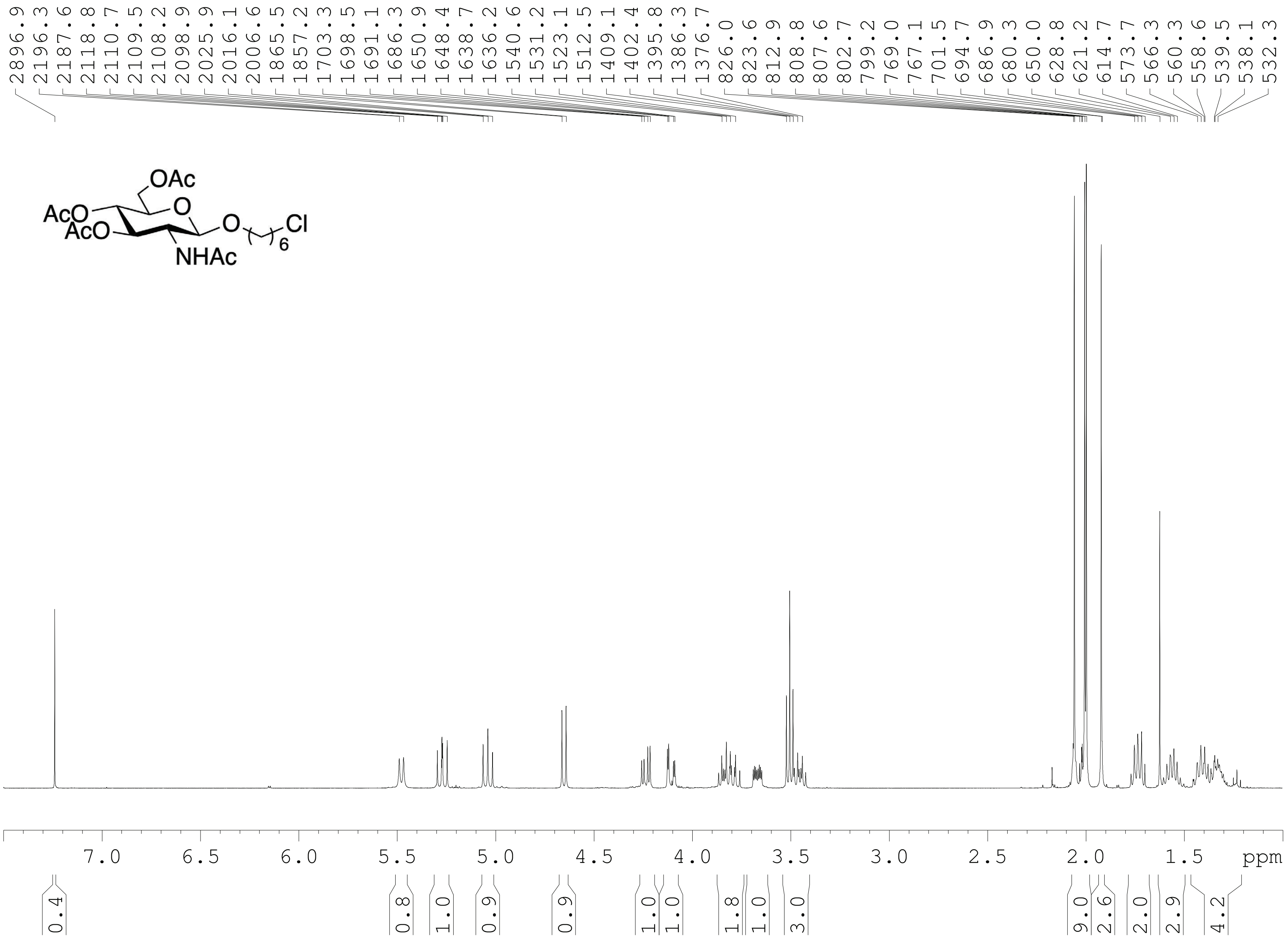

Compound **1**, $CDCl_3$, 400 MHz

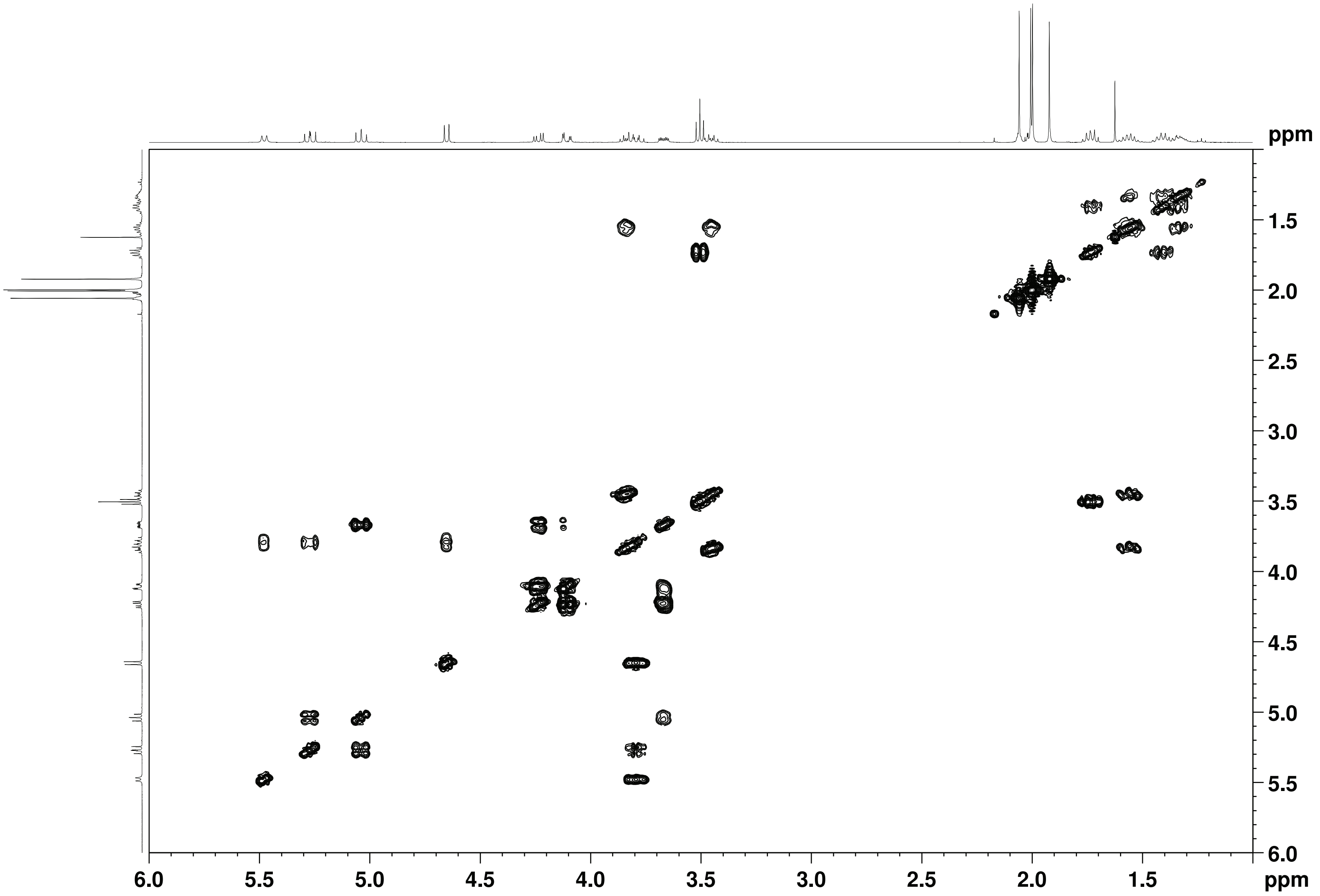

Compound **1**, $CDCl_3$, 100 MHz

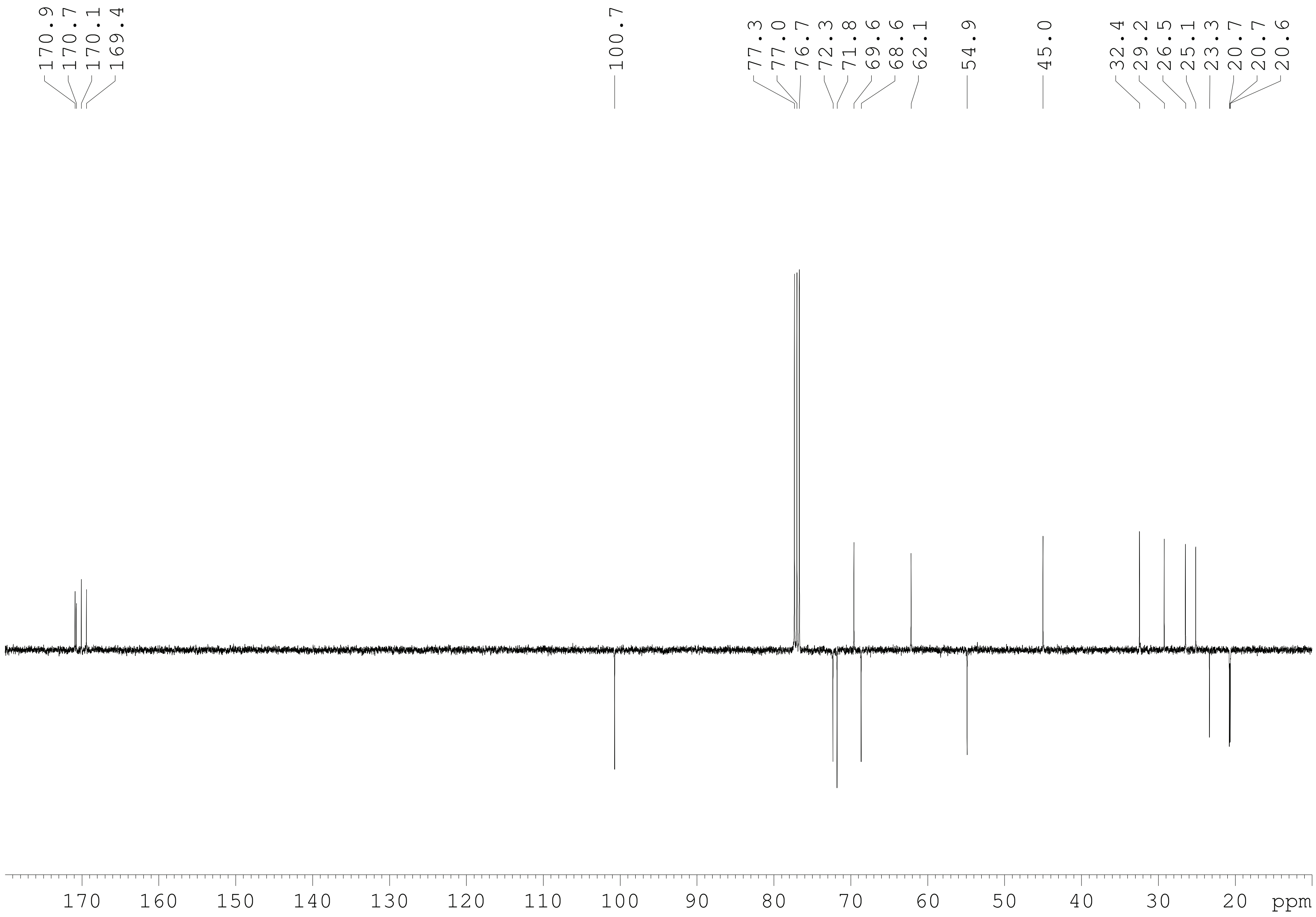

Compound **1**, $CDCl_3$, 400 MHz

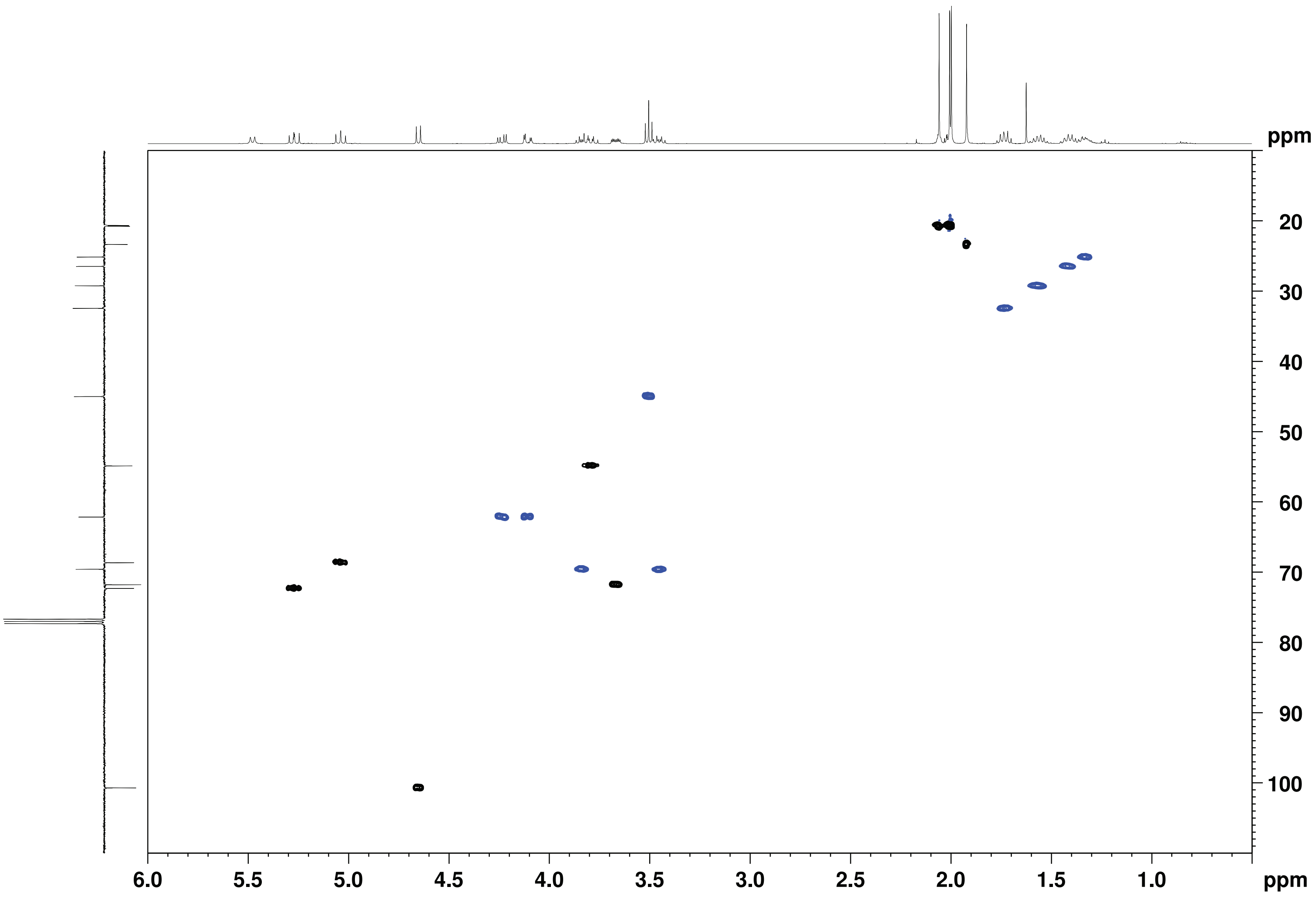

Compound **5β**, $CDCl_3$, 400 MHz

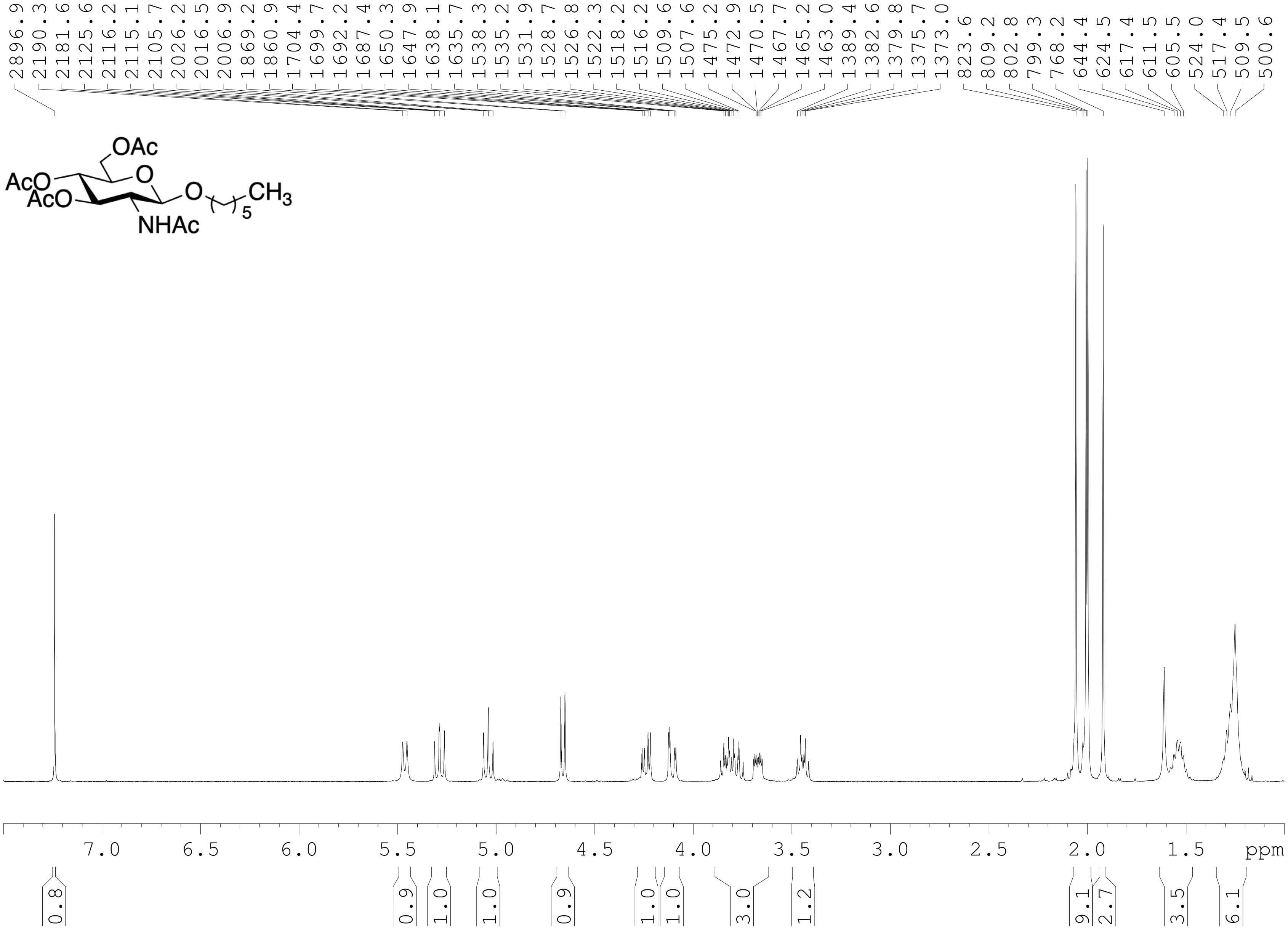

Compound **5β**, $CDCl_3$, 400 MHz

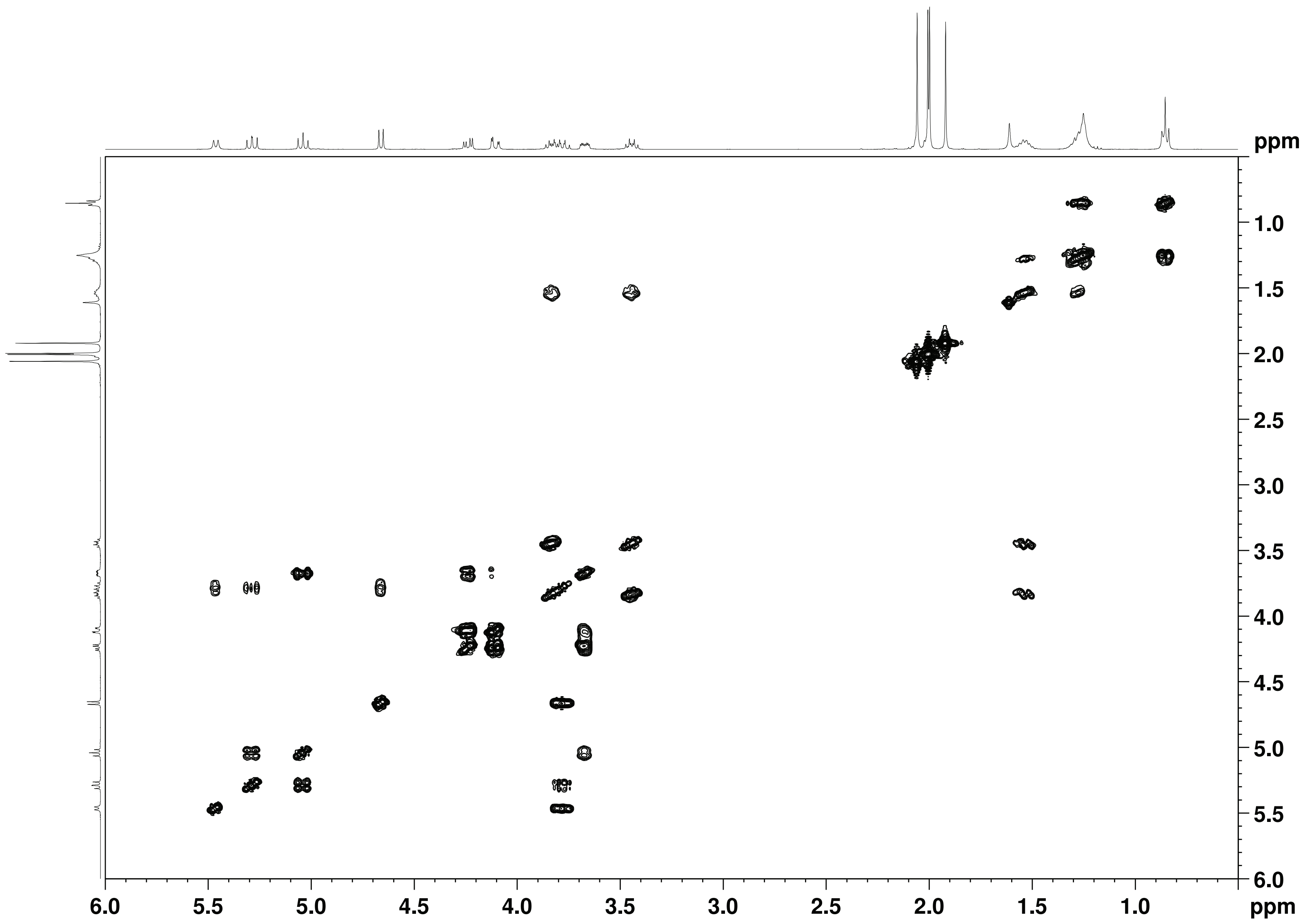

Compound **5β**, $CDCl_3$, 100 MHz

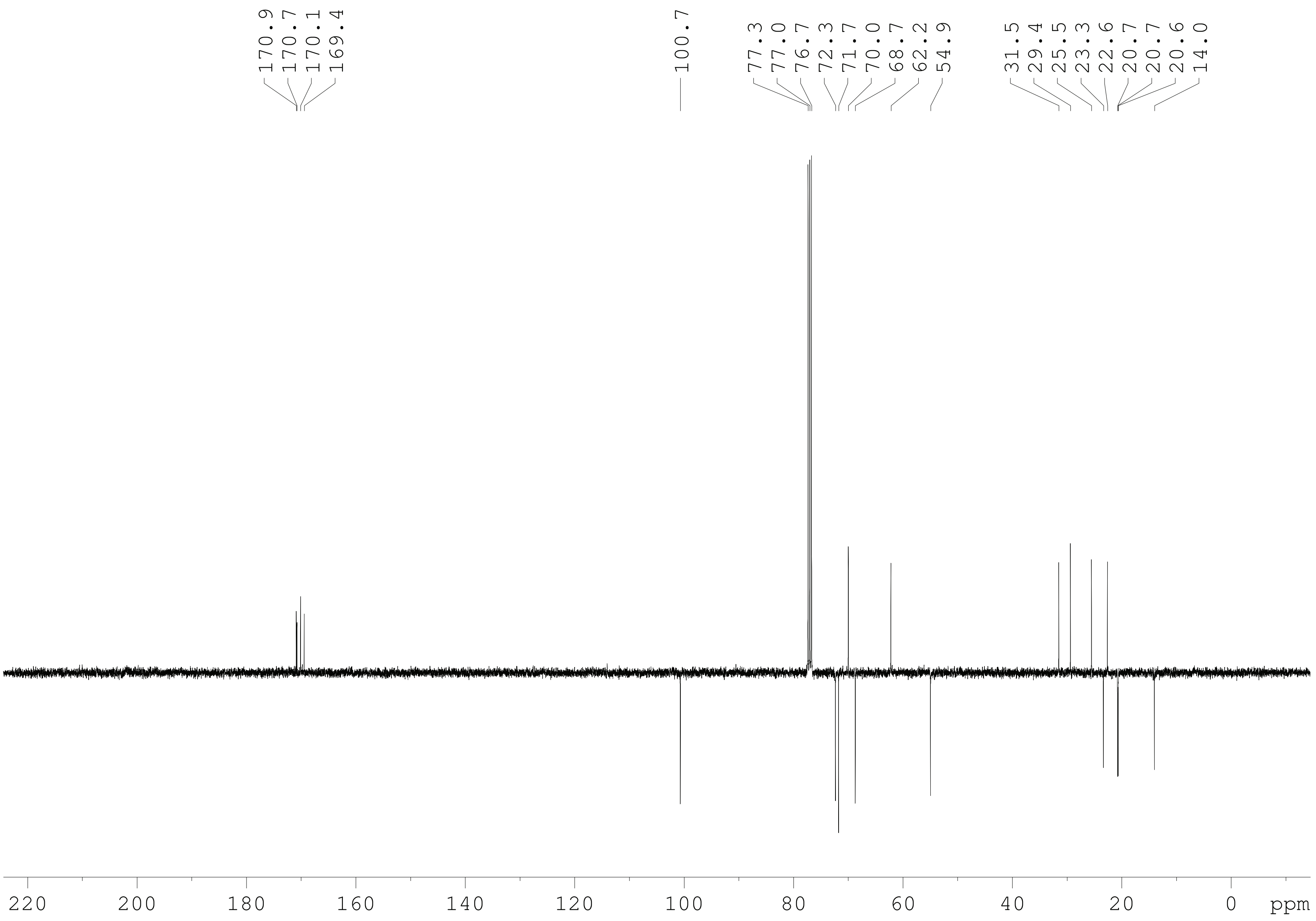

Compound **5β**, $CDCl_3$, 400 MHz

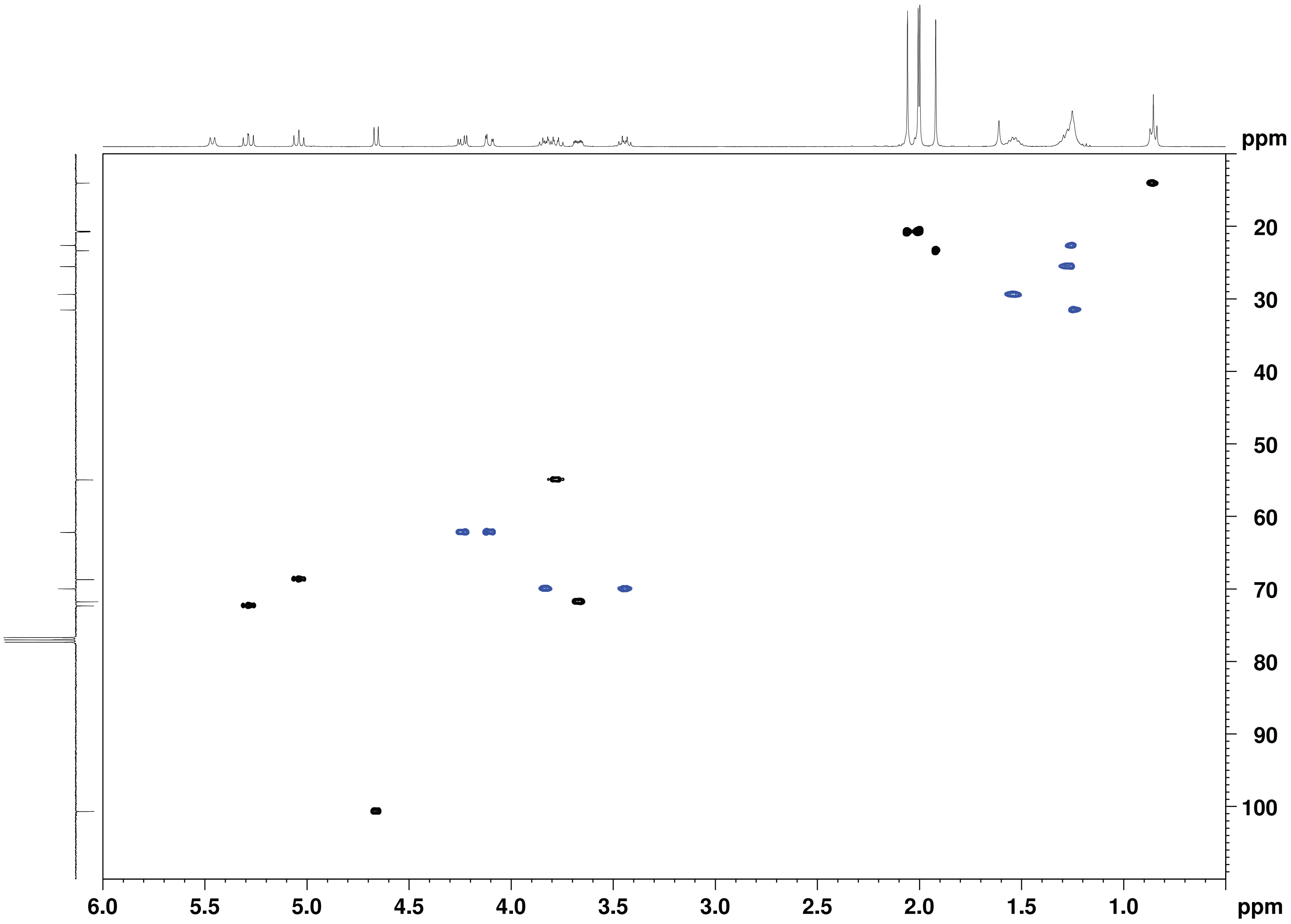

Compound **6β**, $CDCl_3$, 400 MHz

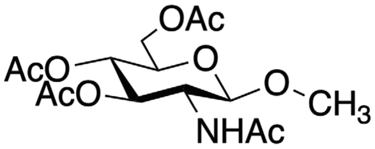

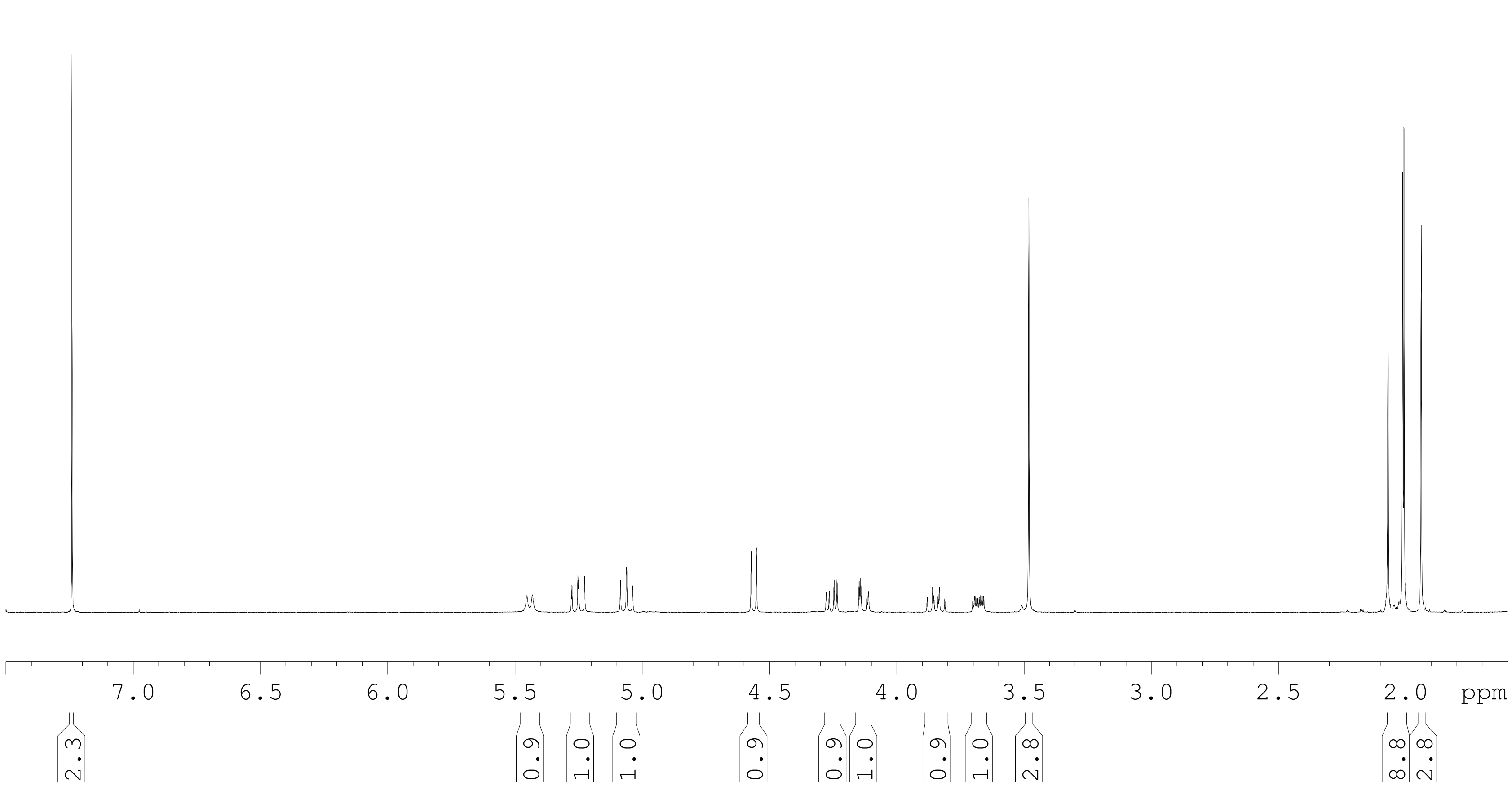

Compound **6β**, $CDCl_3$, 400 MHz

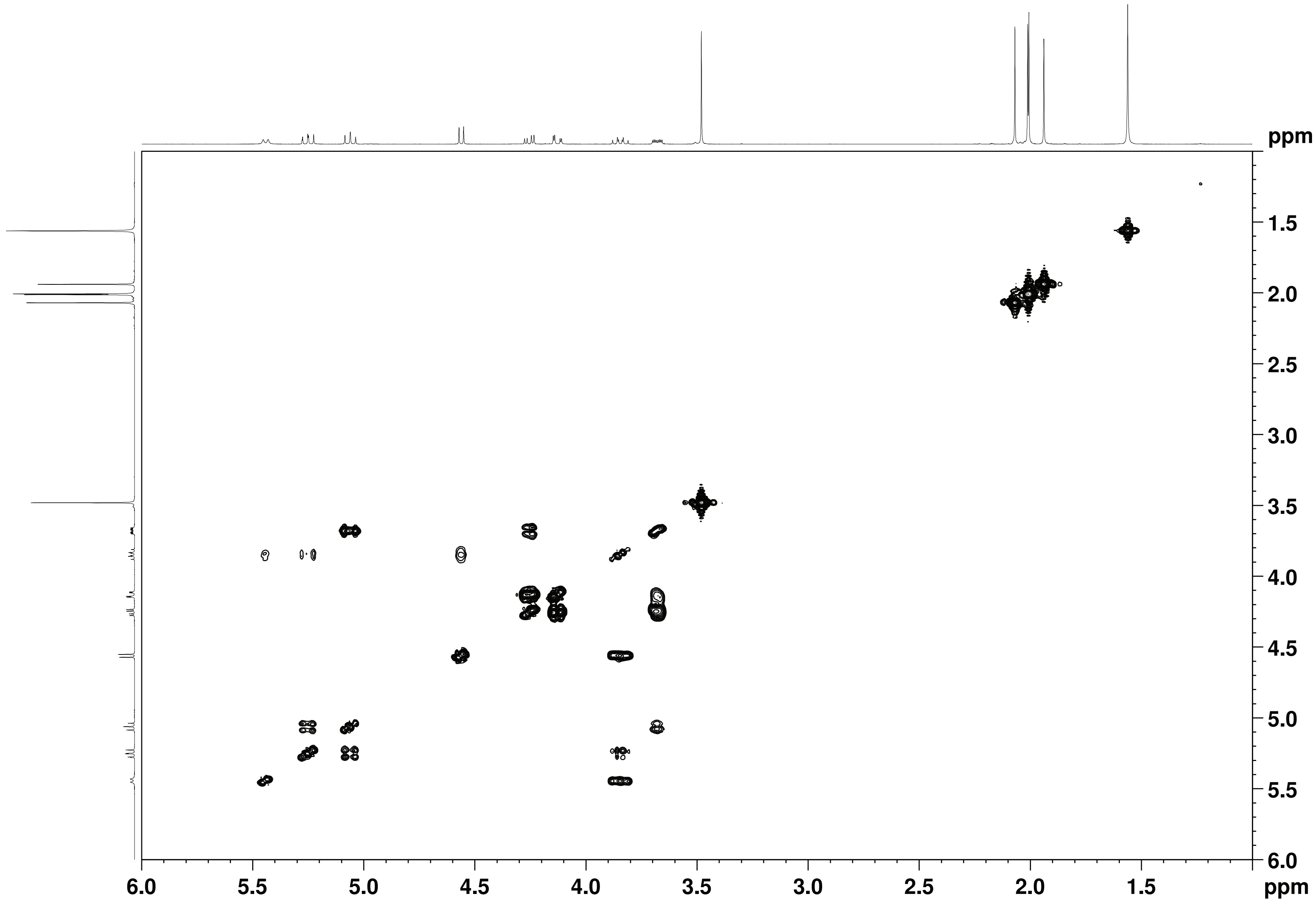

Compound **6β**, $CDCl_3$, 100 MHz

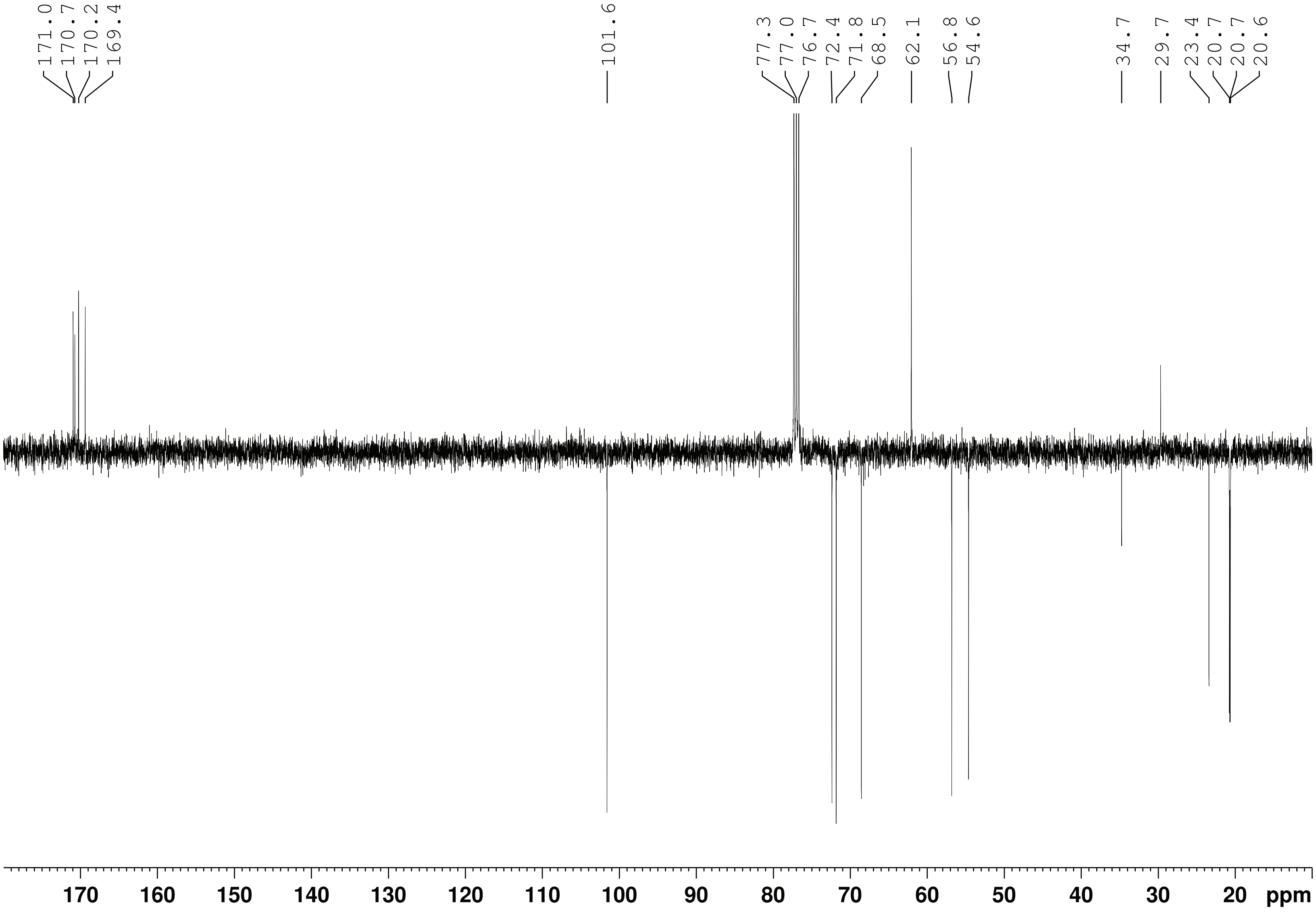

Compound **6β**, $CDCl_3$, 400 MHz

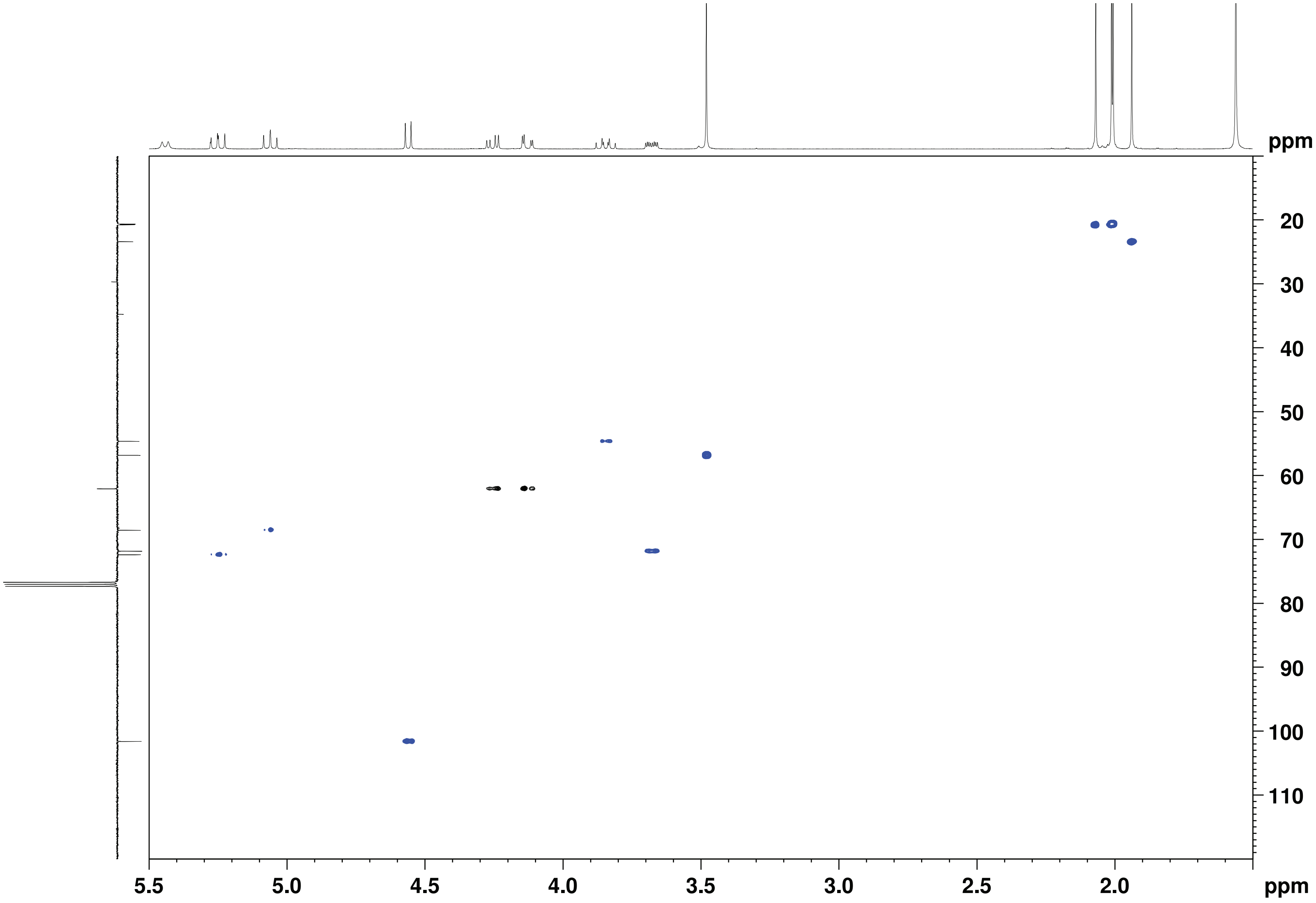

Compound **5α/β** (9:1), $CDCl_3$, 400 MHz

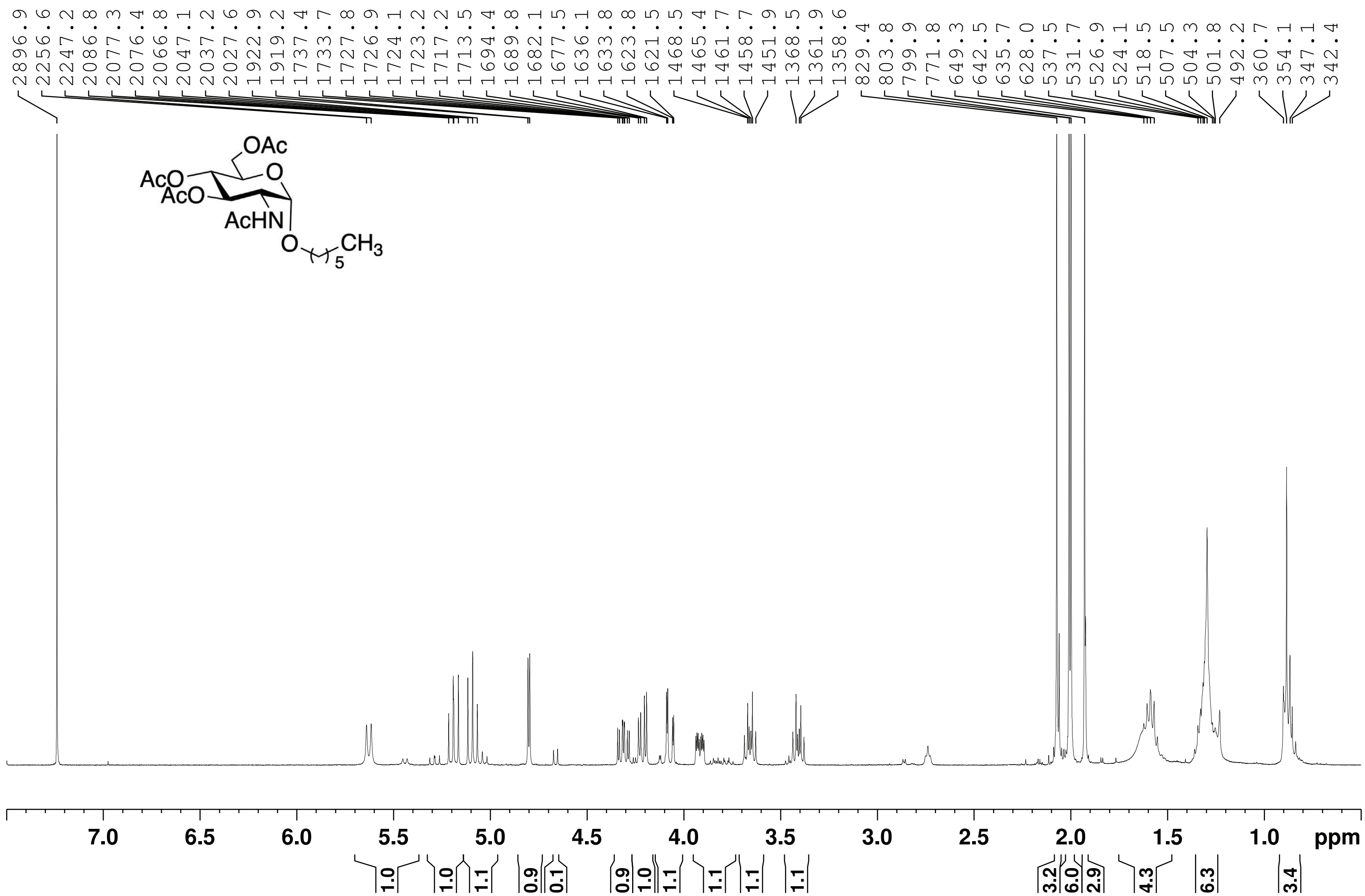

Compound **5α/β** (9:1), $CDCl_3$, 600 MHz

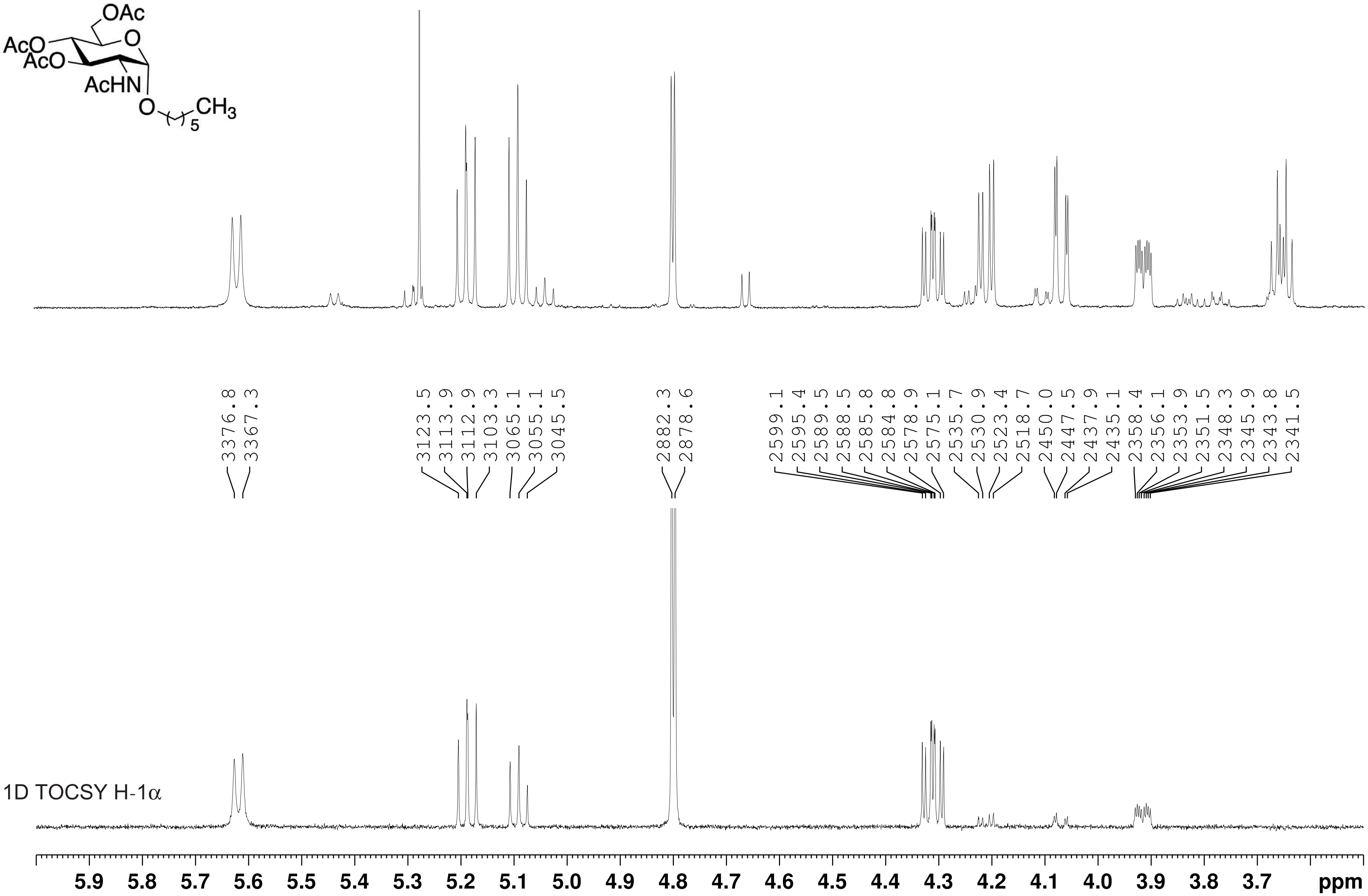

Compound **5α/β** (9:1), $CDCl_3$, 400 MHz

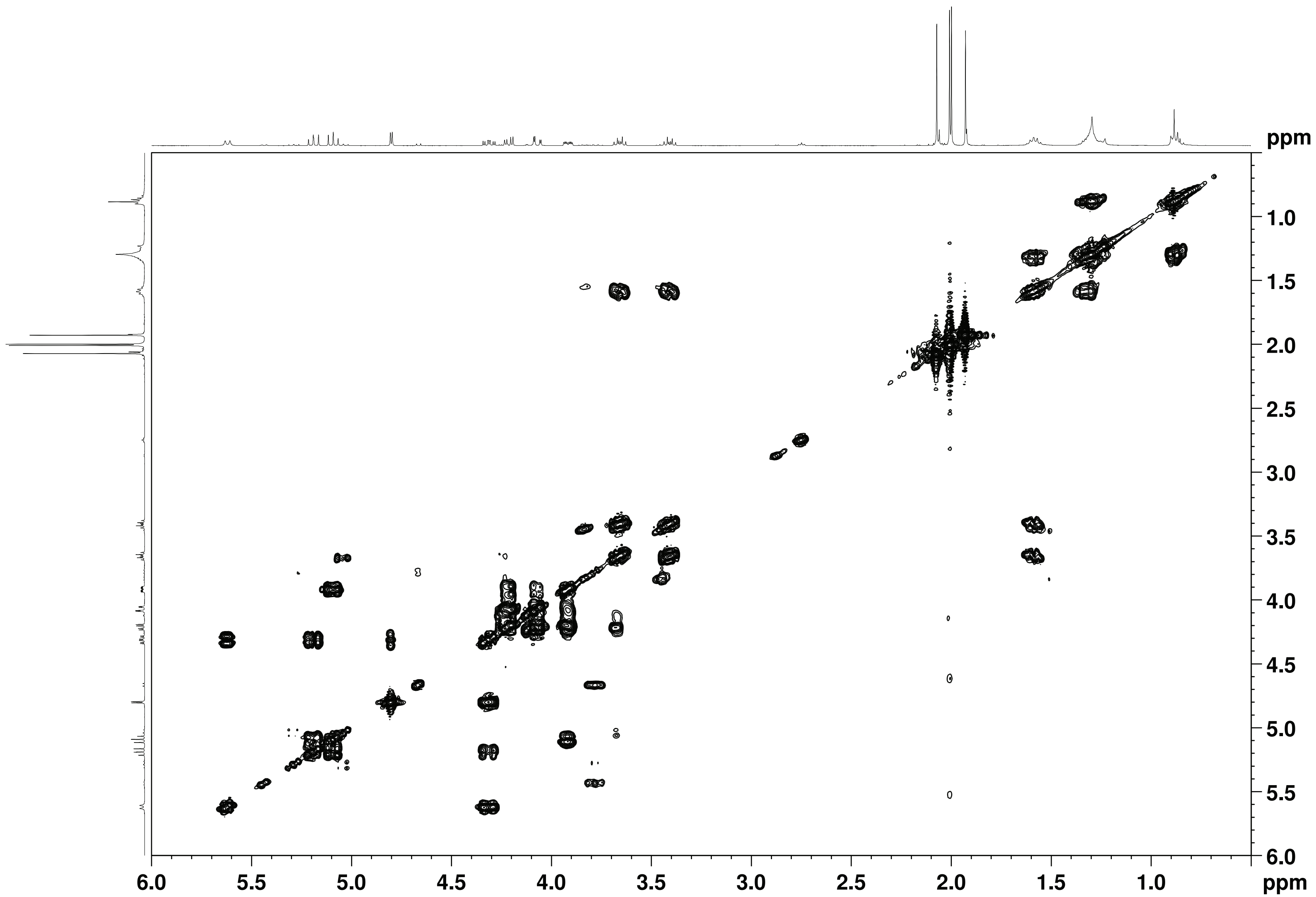

Compound **5α/β** (9:1), $CDCl_3$, 100 MHz

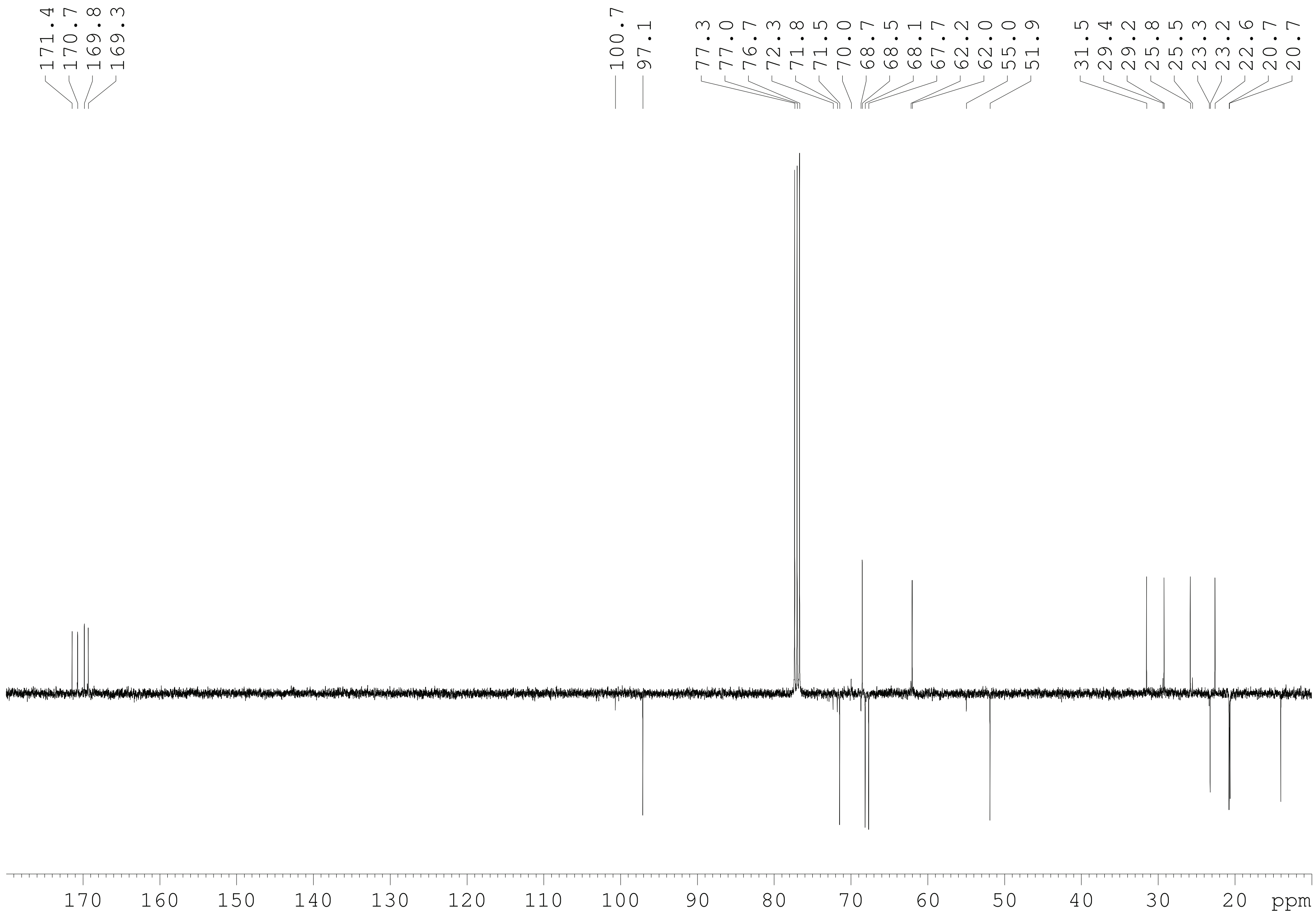

Compound **5α/β** (9:1), $CDCl_3$, 400 MHz

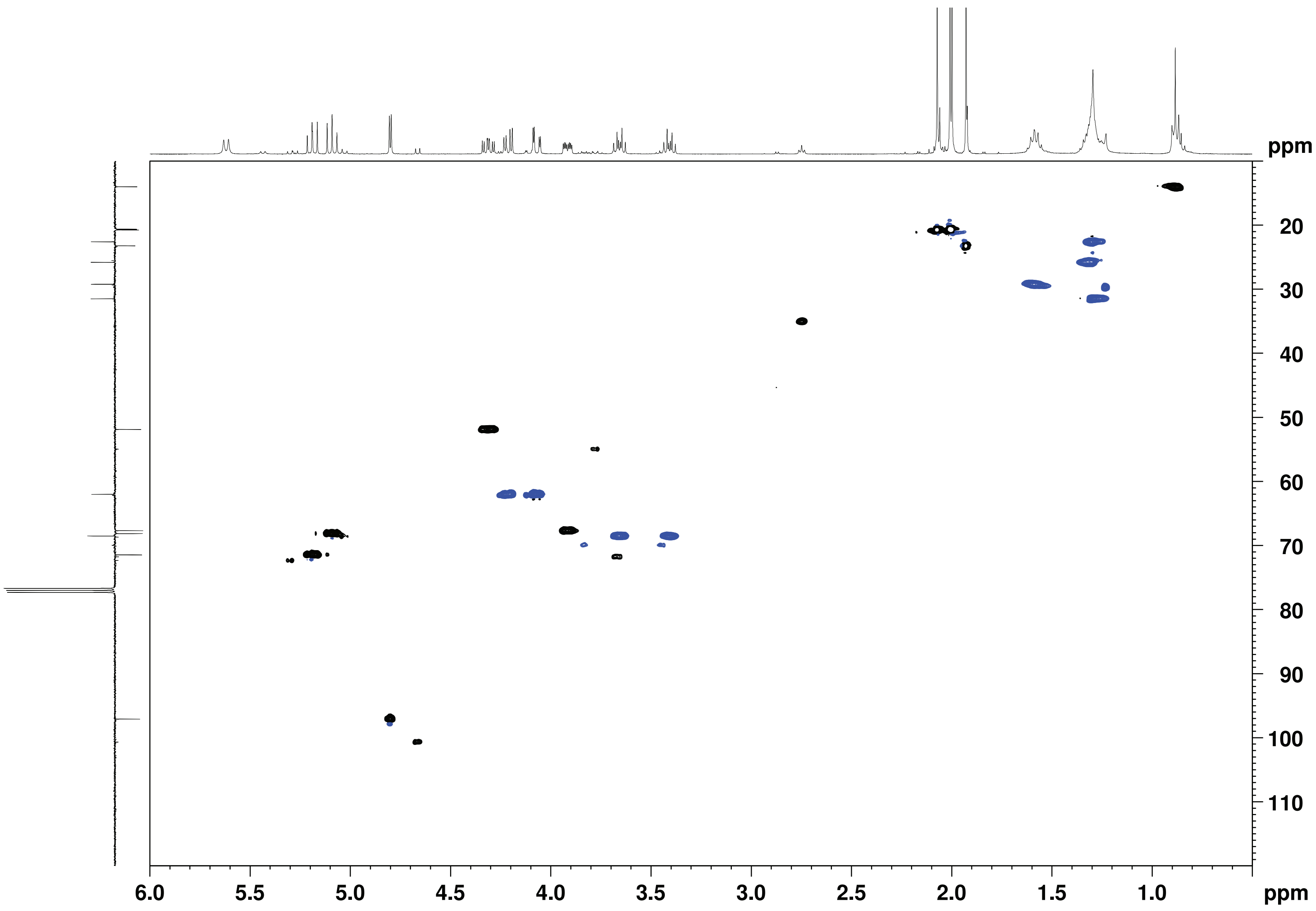

Compound **6**α/β (9:1), $CDCl_3$, 400 MHz

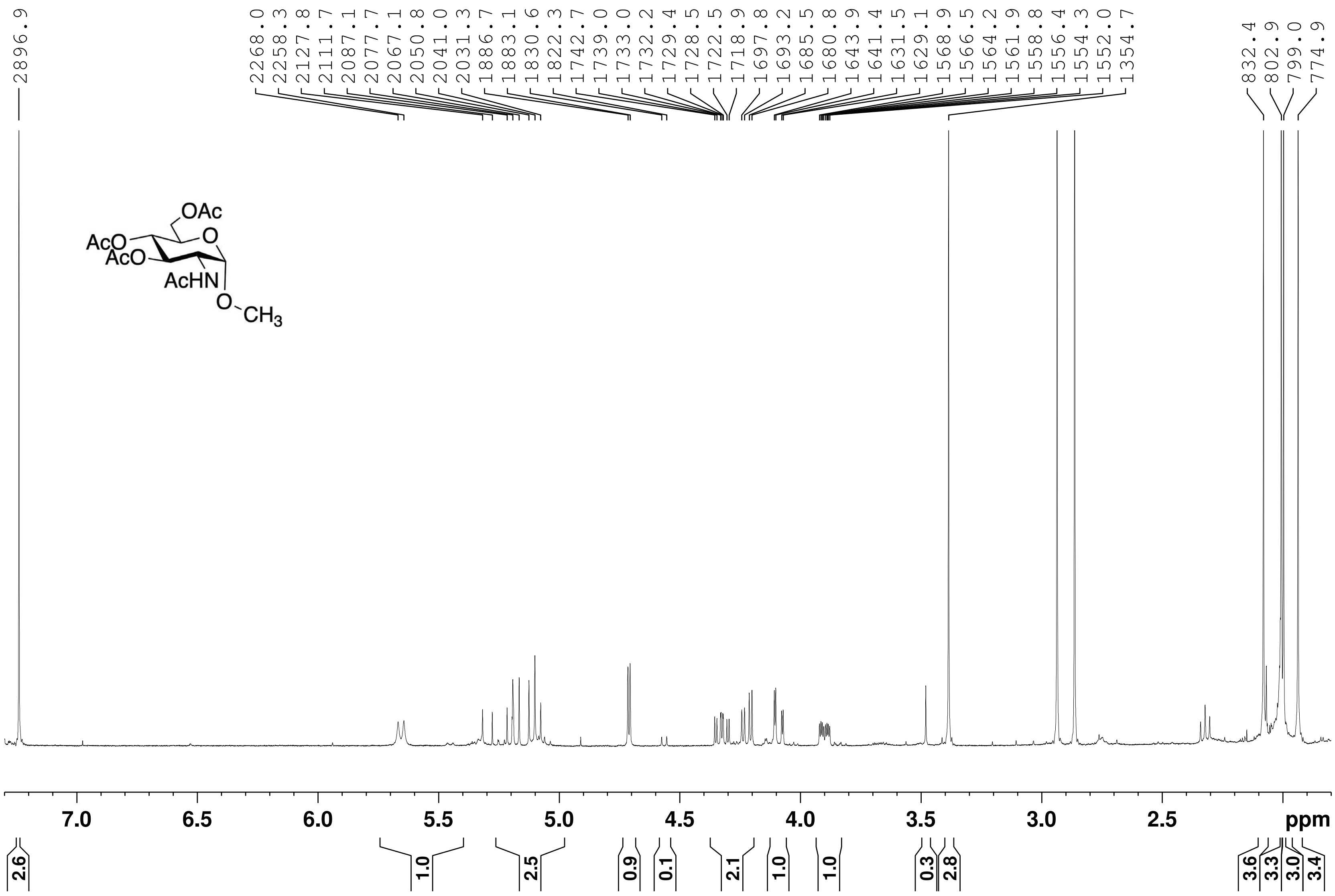

Compound **6α/β** (9:1), $CDCl_3$, 600 MHz

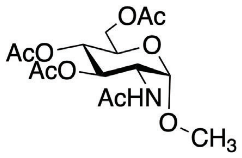

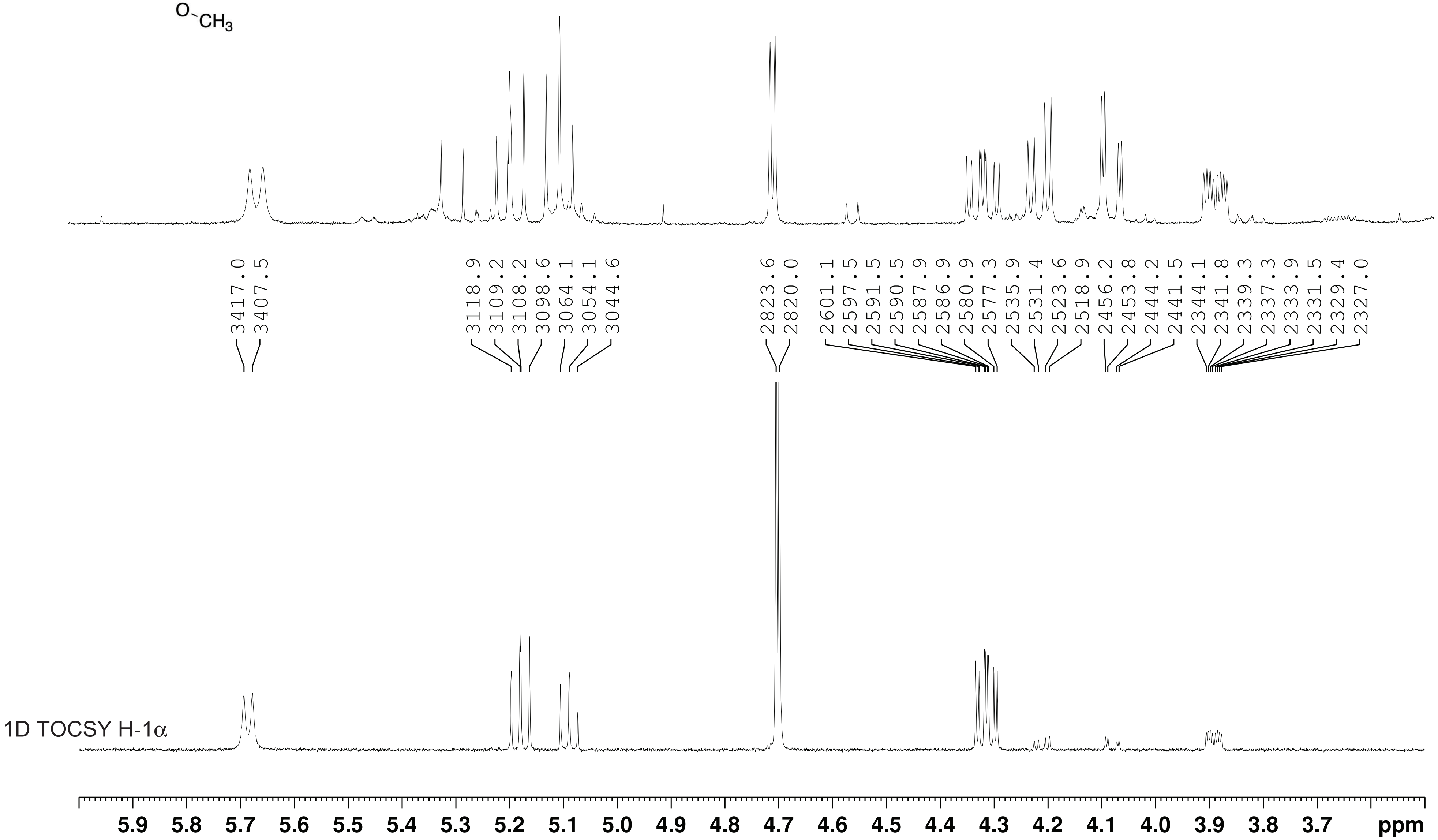

Compound **6α/β** (9:1), $CDCl_3$, 400 MHz

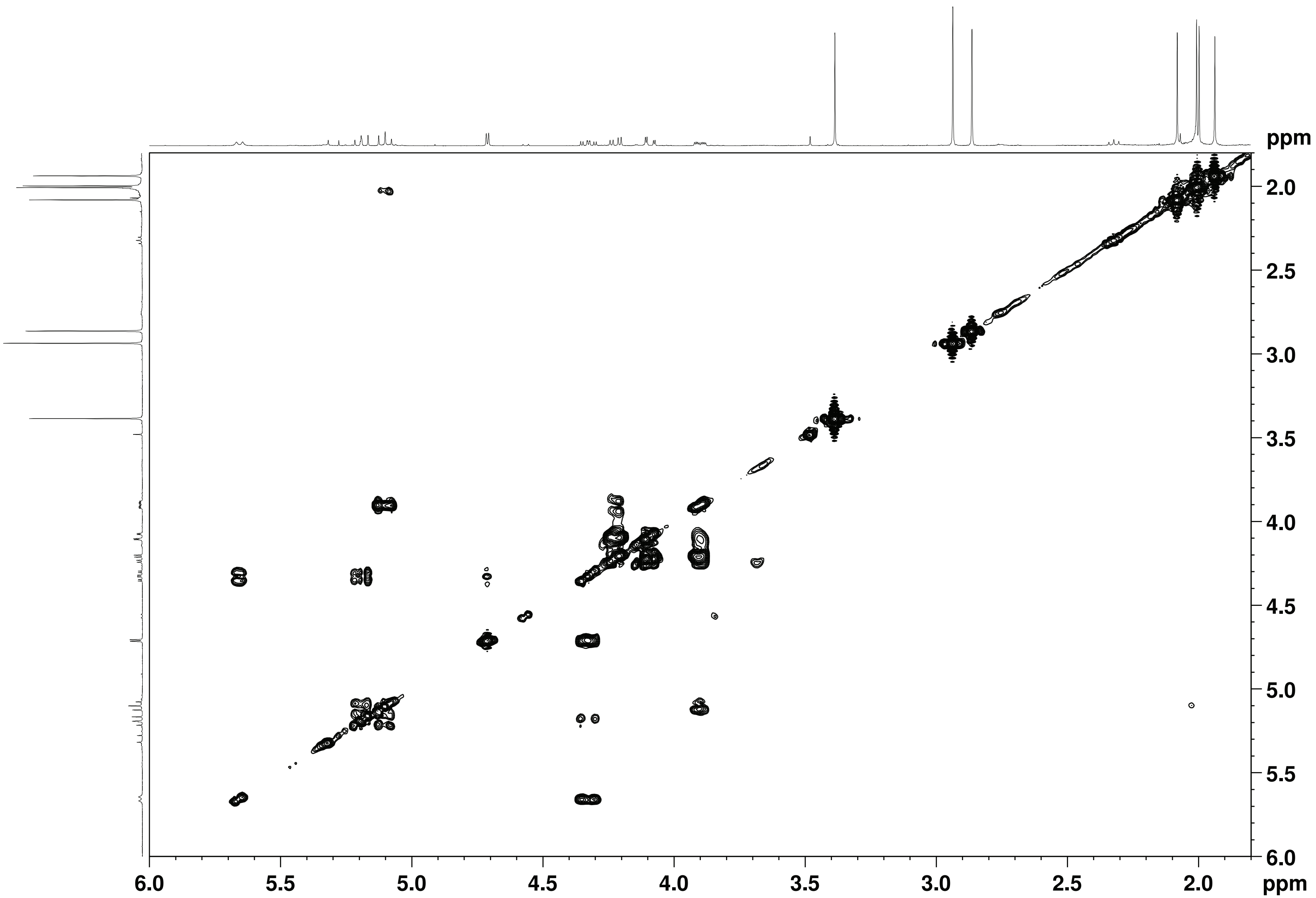

Compound **6**α/β (9:1), $CDCl_3$, 100 MHz

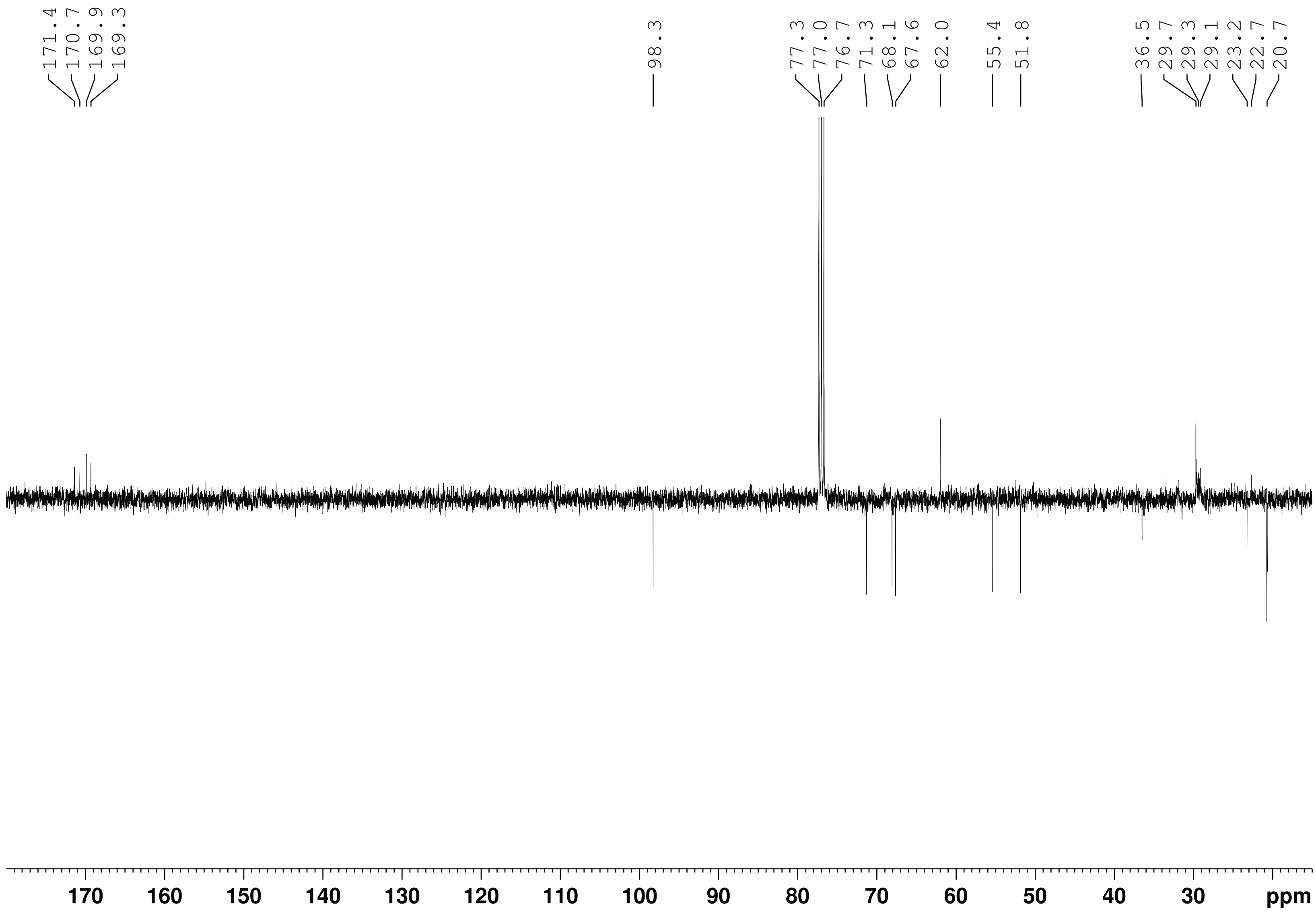

Compound **6**α/β (9:1), $CDCl_3$, 400 MHz

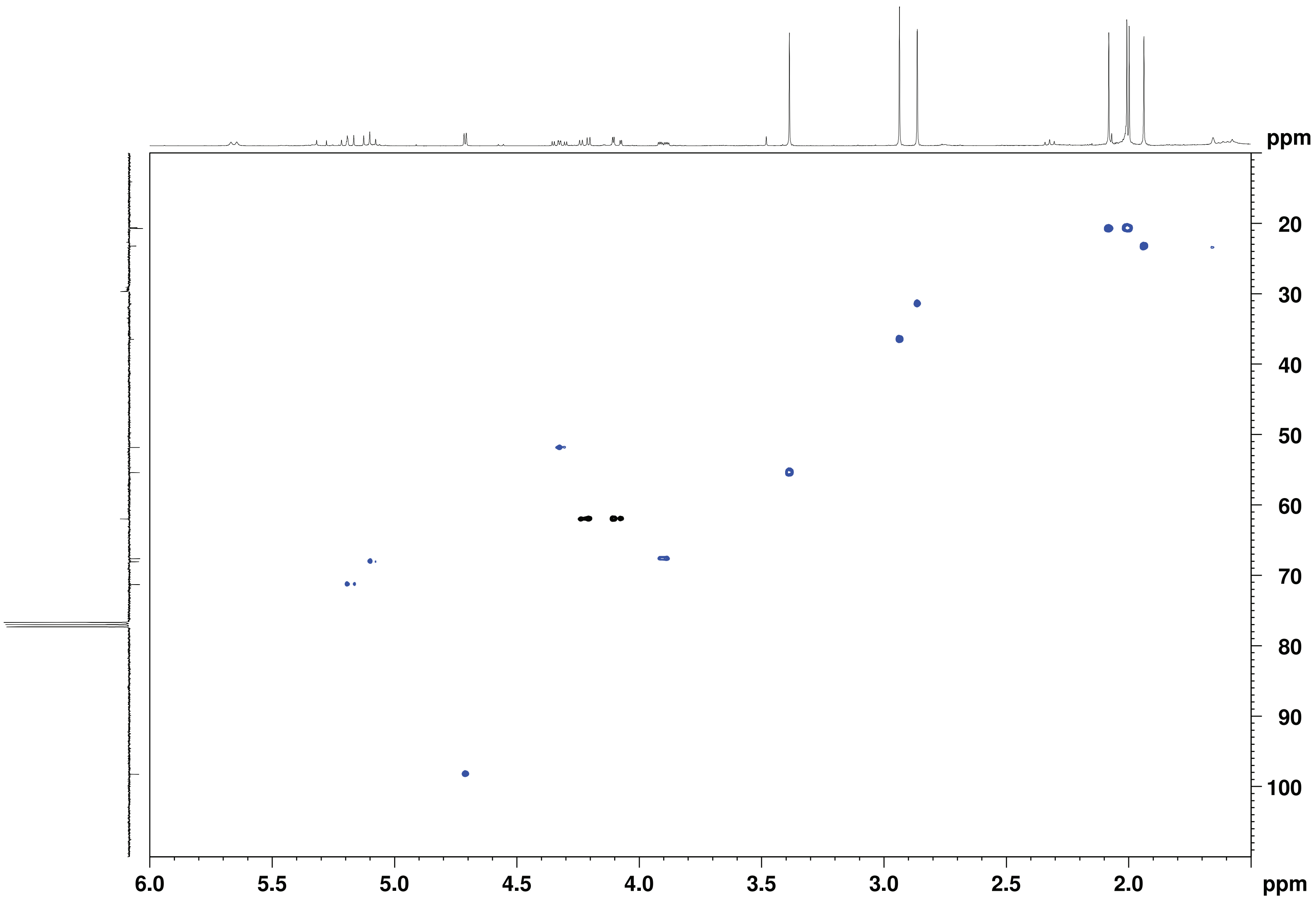

Solids, d_6-DMSO, 400 MHz

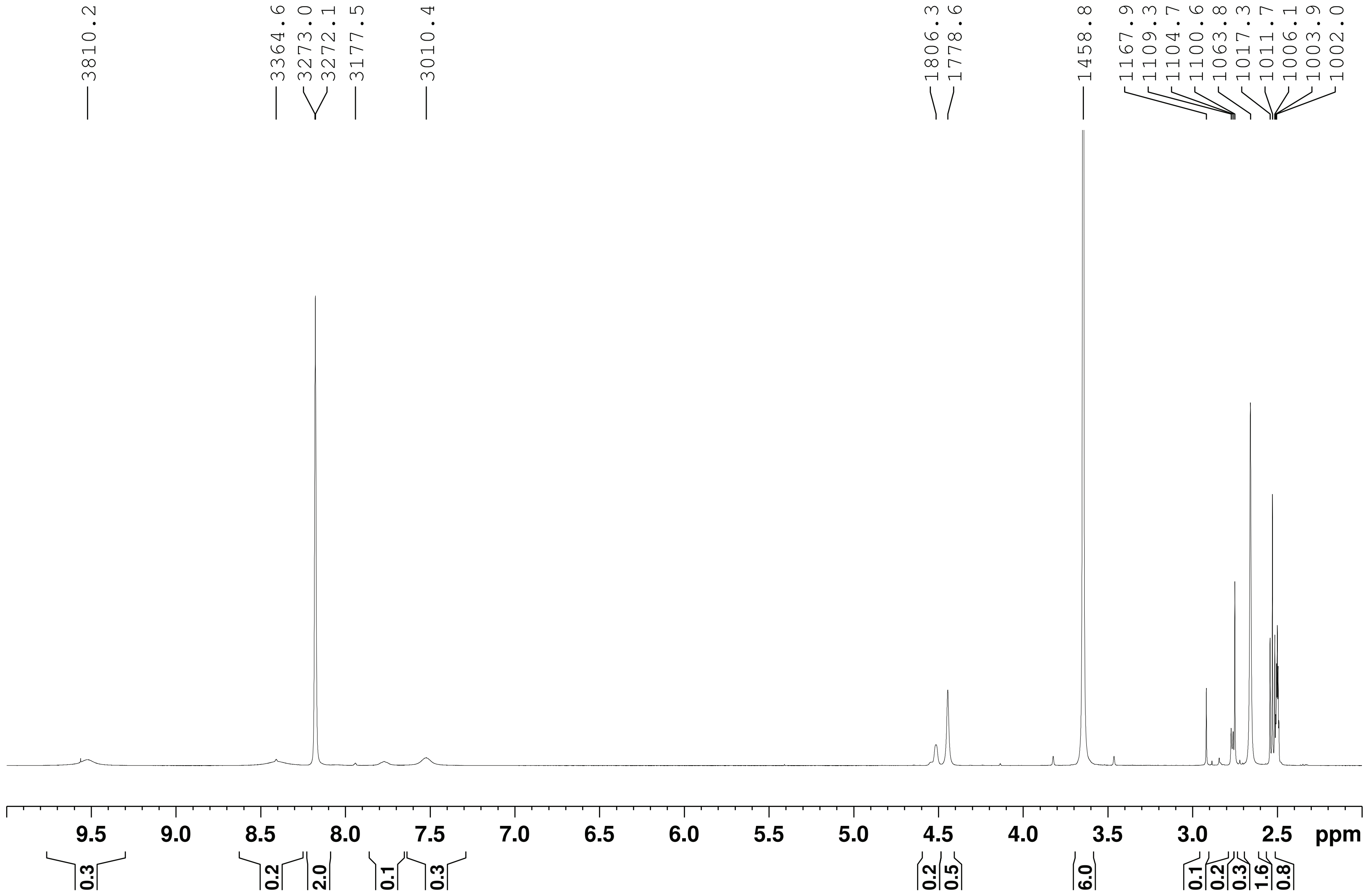

Solids, d_6-DMSO, 400 MHz

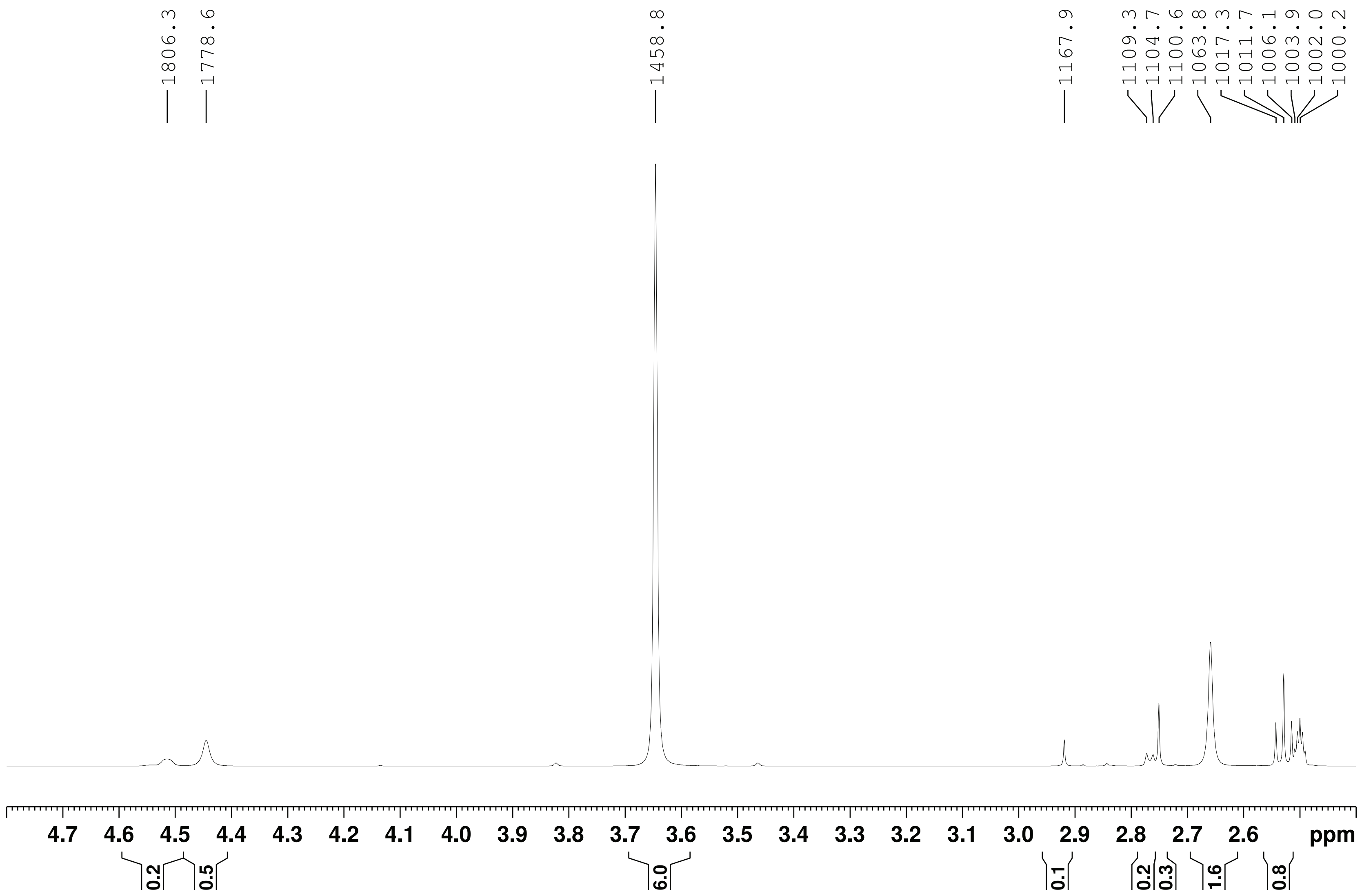

Solids, d_6-DMSO, 400 MHz

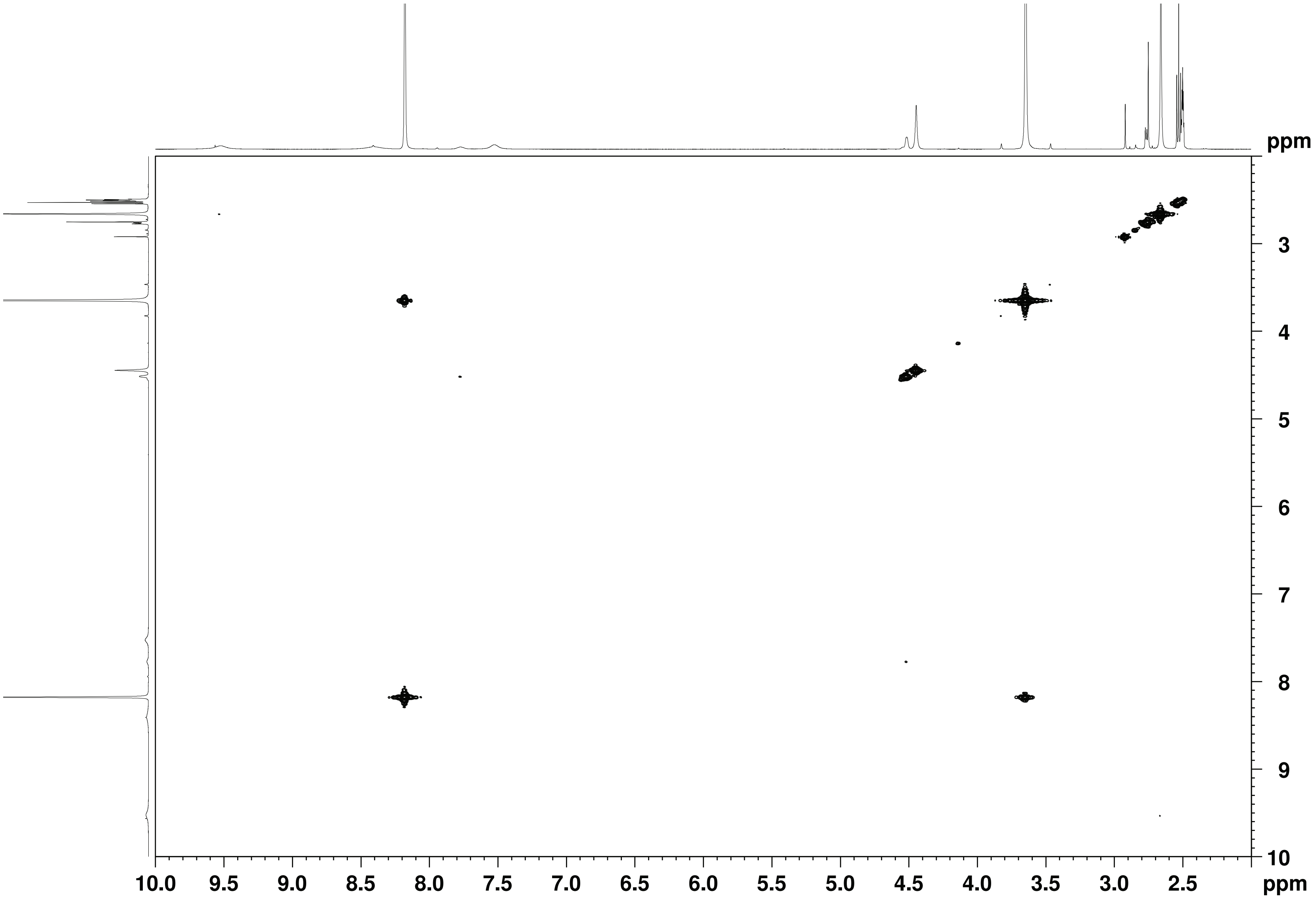

Solids, d_6-DMSO, 100 MHz

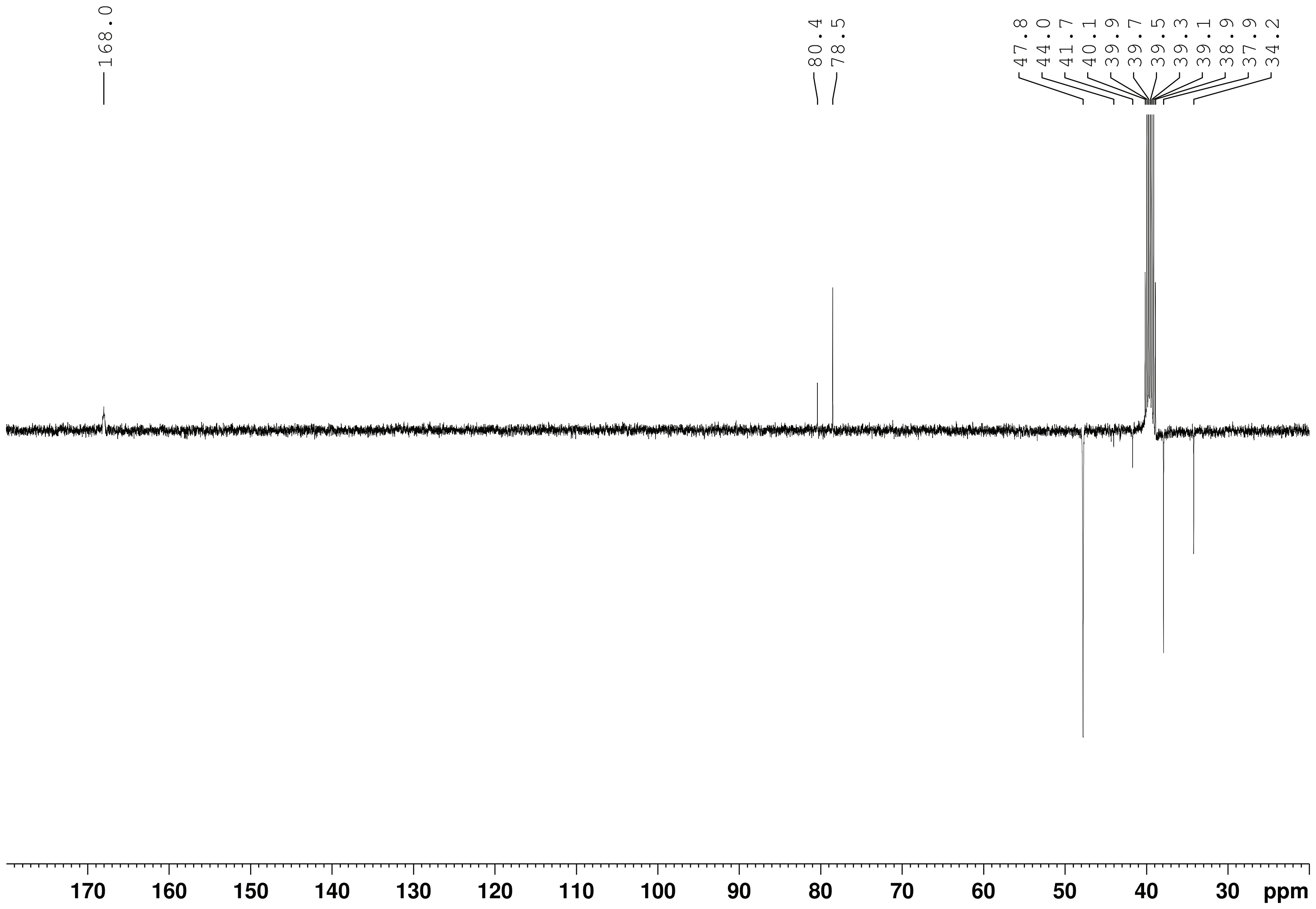

Solids, d_6-DMSO, 100 MHz

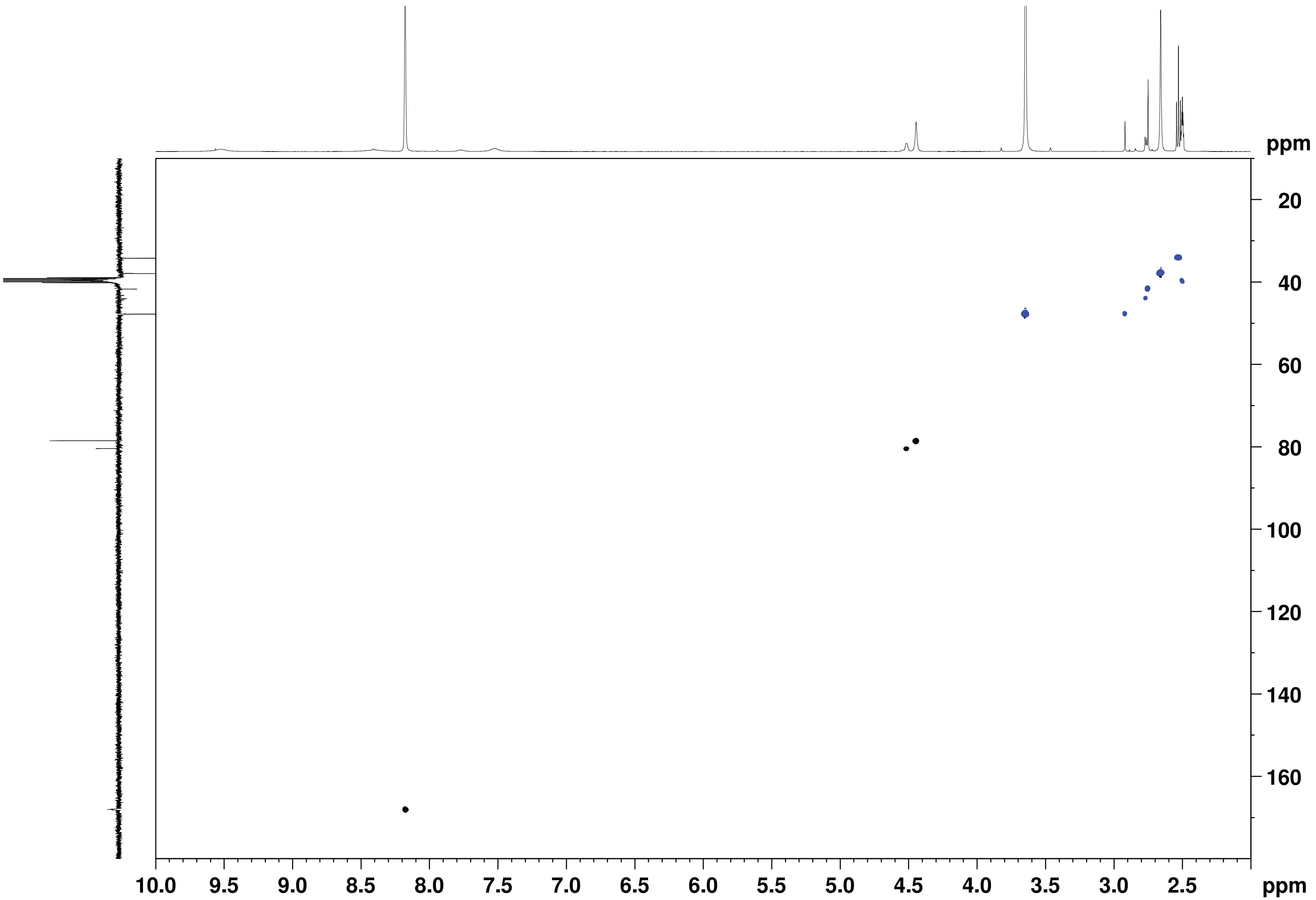

Supernatant, d_6-DMSO, 400 MHz

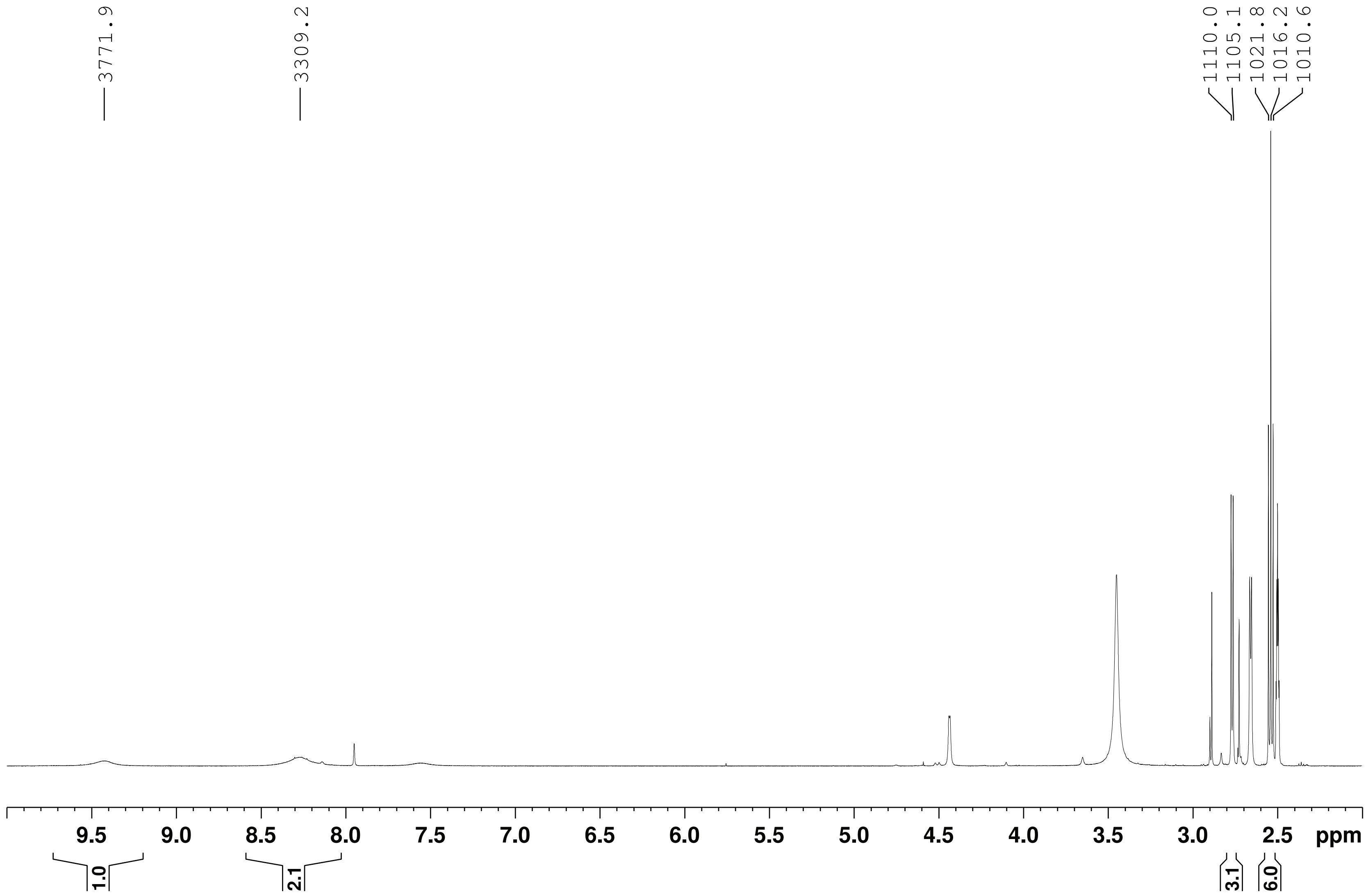

Supernatant, d_6-DMSO, 400 MHz

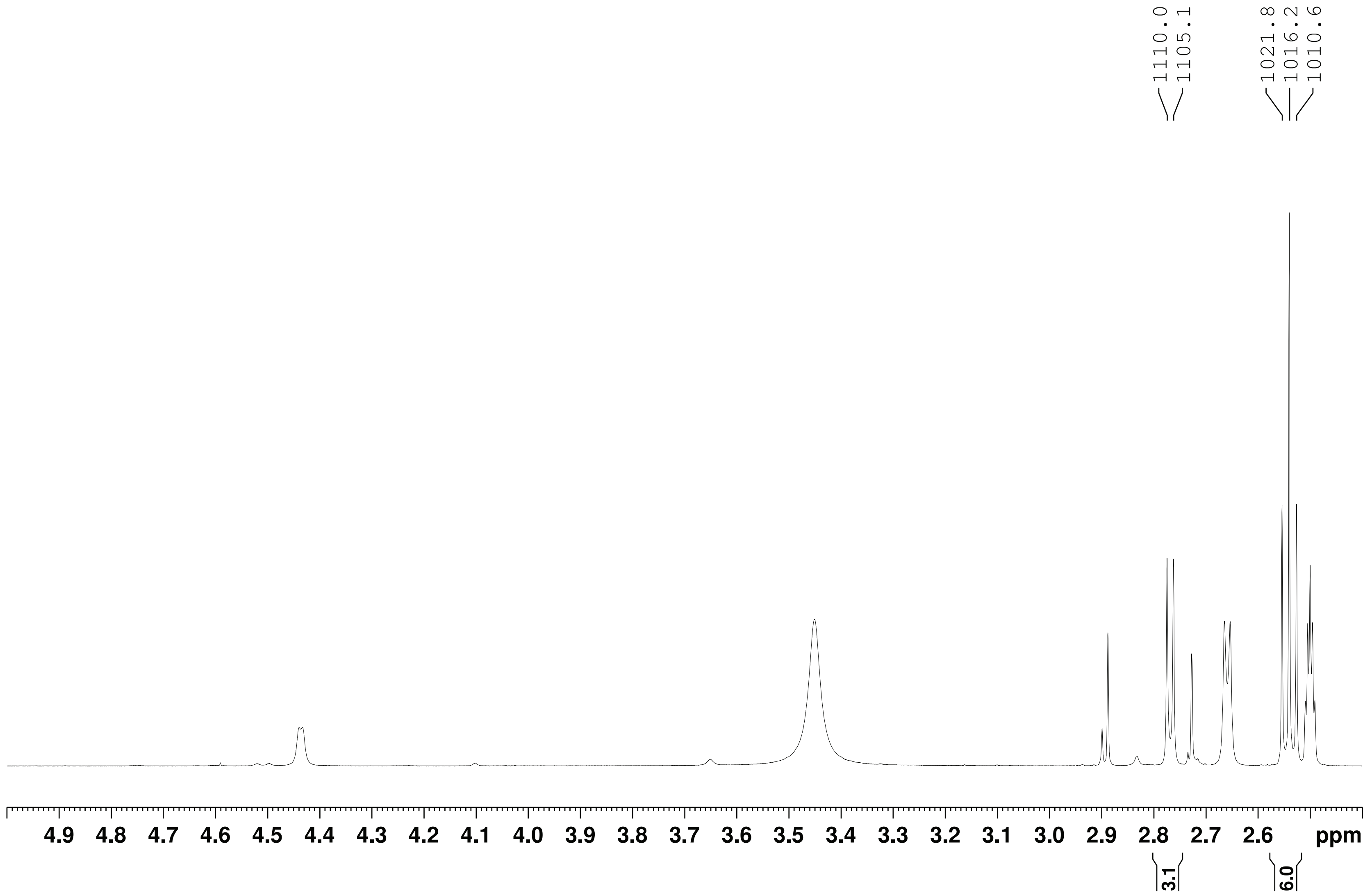

Supernatant, *d*$_6$-DMSO, 400 MHz

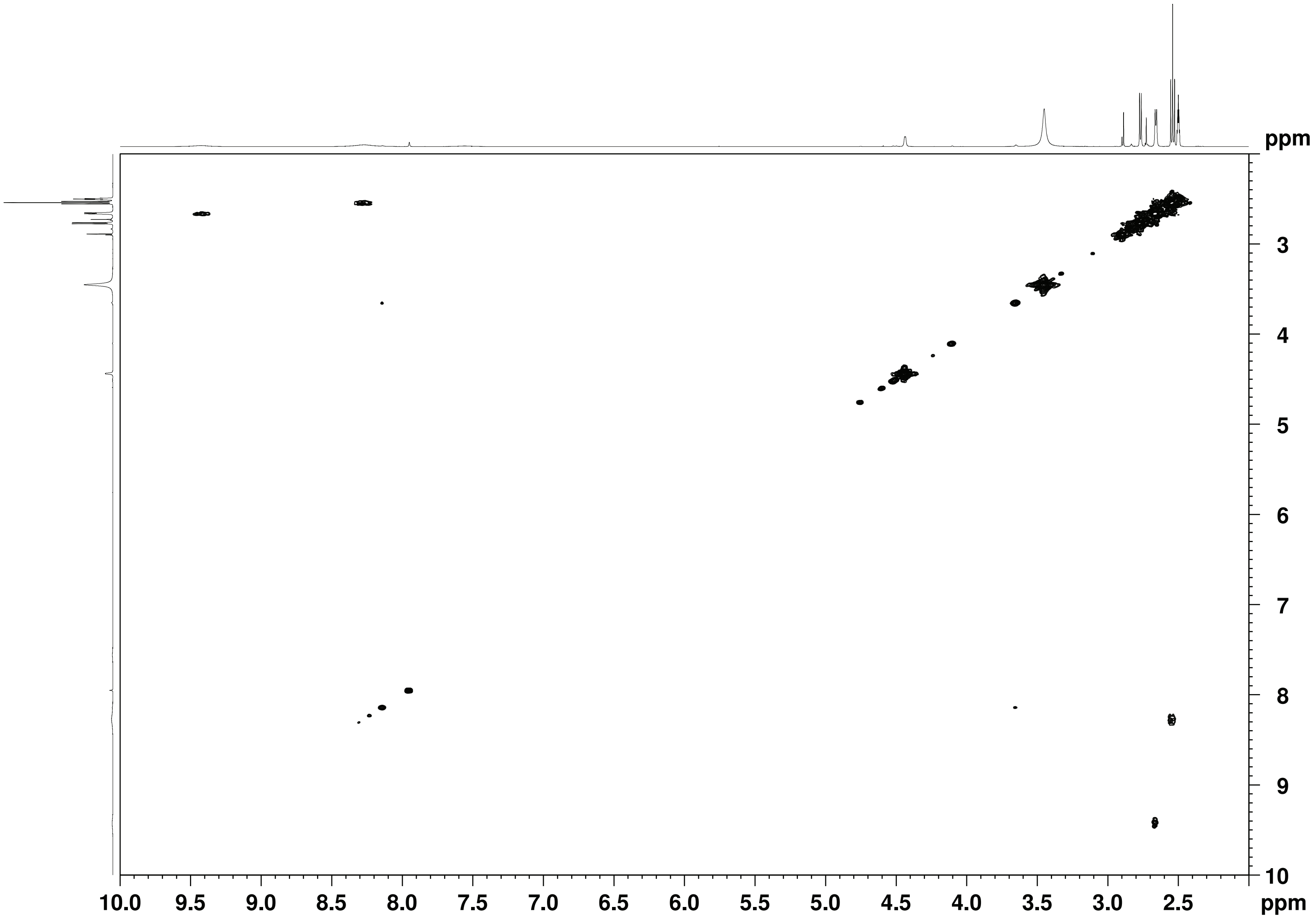

Supernatant, *d*$_6$-DMSO, 100 MHz

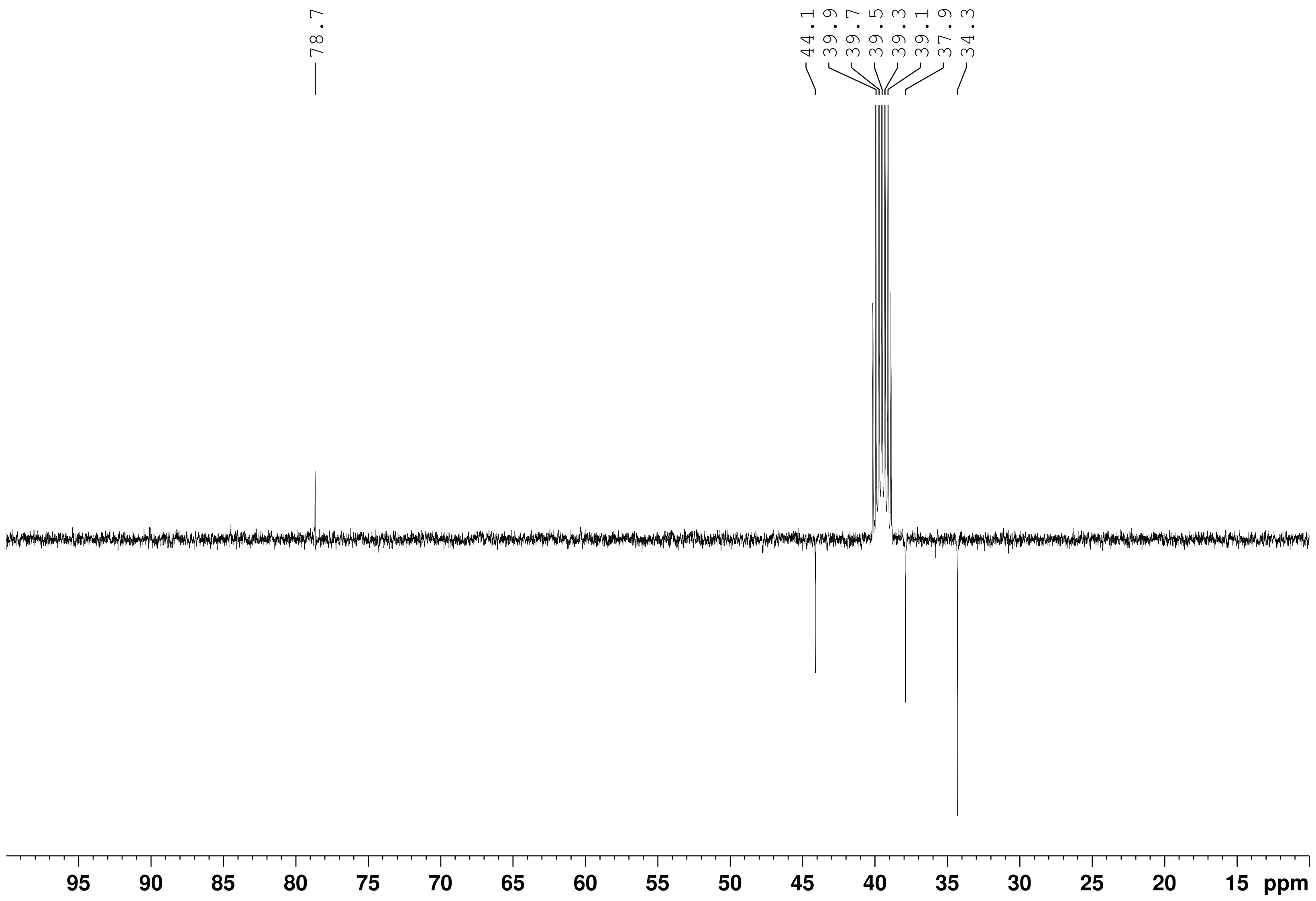

Supernatant, *d*$_6$-DMSO, 400 MHz

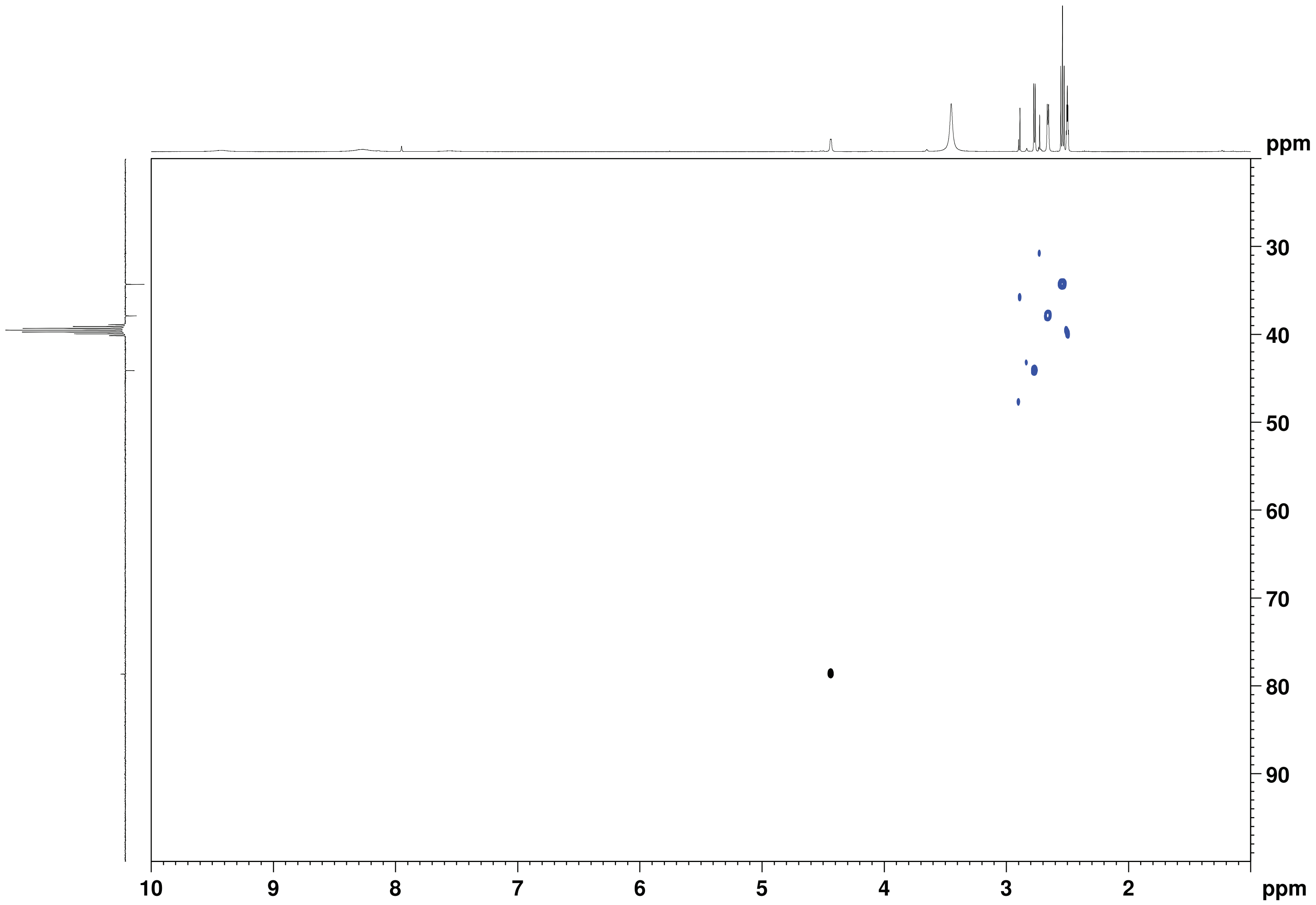

Supplement: Supplementary file 1 [file molecules-30-01483-s001.zip › molecules-3522470-supplementary.pdf]
